# Causes of death identified in neonates enrolled through Child Health and Mortality Prevention Surveillance (CHAMPS), December 2016 –December 2021

**DOI:** 10.1371/journal.pgph.0001612

**Published:** 2023-03-20

**Authors:** Sana Mahtab, Shabir A. Madhi, Vicky L. Baillie, Toyah Els, Bukiwe Nana Thwala, Dickens Onyango, Beth A. Tippet-Barr, Victor Akelo, Kitiezo Aggrey Igunza, Richard Omore, Shams El Arifeen, Emily S. Gurley, Muntasir Alam, Atique Iqbal Chowdhury, Afruna Rahman, Quique Bassat, Inacio Mandomando, Sara Ajanovic, Antonio Sitoe, Rosauro Varo, Samba O. Sow, Karen L. Kotloff, Henry Badji, Milagritos D. Tapia, Cheick B. Traore, Ikechukwu U. Ogbuanu, James Bunn, Ronita Luke, Sulaiman Sannoh, Alim Swarray-Deen, Nega Assefa, J. Anthony G. Scott, Lola Madrid, Dadi Marami, Surafel Fentaw, Maureen H. Diaz, Roosecelis B. Martines, Robert F. Breiman, Zachary J. Madewell, Dianna M. Blau, Cynthia G. Whitney, for the CHAMPS Consortium

**Affiliations:** 1 South African Medical Research Council Vaccines and Infectious Diseases Analytics Research Unit, University of the Witwatersrand, Johannesburg, South Africa; 2 Kenya County Department of Health, Kisumu, Kenya; 3 Centers for Disease Control and Prevention, Kisumu, Kenya; 4 Kenya Medical Research Institute-Center for Global Health Research (KEMRI-CGHR), Kisumu, Kenya; 5 International Center for Diarrhoeal Diseases Research (icddr,b), Dhaka, Bangladesh; 6 Department of Epidemiology, Johns Hopkins Bloomberg School of Public Health, Baltimore, Maryland, United States of America; 7 ISGlobal—Hospital Clínic, Unversitat de Barcelona, Barcelona, Spain; 8 Centro de Investigação em Saúde de Manhiça [CISM], Maputo, Mozambique; 9 Institutó Catalana de Recerca I Estudis Avançats [ICREA], Barcelona, Spain; 10 Hospital Sant Joan de Déu, Universitat de Barcelona, Esplugues, Barcelona, Spain; 11 Consorcio de Investigación Biomédica en Red de Epidemiología y Salud Pública [CIBERESP], Madrid, Spain; 12 Instituto Nacional de Saúde [INS], Maputo, Mozambique; 13 Centre pour le Développement des Vaccins (CVD-Mali), Ministère de la Santé, Bamako, Mali; 14 Department of Pediatrics, Center for Vaccine Development and Global Health, University of Maryland School of Medicine, Baltimore, Maryland, United States of America; 15 Department of Pathological Anatomy and Cytology, University Hospital of Point G, Bamako, Mali; 16 Crown Agents, Freetown, Sierra Leone; 17 World Health Organization–Sierra Leone, Freetown, Sierra Leone; 18 Ola During Children’s Hospital, Freetown, Sierra Leone; 19 St. Luke’s University Health Network, Easton, Pennsylvania, United States of America; 20 University of Ghana Medical School, Accra, Ghana; 21 College of Health and Medical Sciences, Haramaya University, Harar, Ethiopia; 22 Department of Infectious Epidemiology, London School of Hygiene & Tropical Medicine, London, United Kingdom; 23 Bacterial and Mycology Unit, Ethiopian Public Health Institute, Addis Ababa, Ethiopia; 24 Respiratory Diseases Branch, Division of Bacterial Diseases, National Center for Immunization and Respiratory Diseases, US Centers for Disease Control and Prevention, Atlanta, Georgia, United States of America; 25 Infectious Diseases Pathology Branch, Division of High-Consequence Pathogens and Pathology, National Center for Emerging and Zoonotic Infectious Diseases, US Centers for Disease Control and Prevention, Atlanta, Georgia, United States of America; 26 Rollins School of Public Health, Emory University, Atlanta, Georgia, United States of America; 27 Center for Global Health, US Centers for Disease Control and Prevention, Atlanta, Georgia, United States of America; 28 Emory Global Health Institute, Emory University, Atlanta, Georgia, United States of America; Jhpiego, UNITED STATES

## Abstract

Each year, 2.4 million children die within their first month of life. Child Health and Mortality Prevention Surveillance (CHAMPS) established in 7 countries aims to generate accurate data on why such deaths occur and inform prevention strategies. Neonatal deaths that occurred between December 2016 and December 2021 were investigated with MITS within 24–72 hours of death. Testing included blood, cerebrospinal fluid and lung cultures, multi-pathogen PCR on blood, CSF, nasopharyngeal swabs and lung tissue, and histopathology examination of lung, liver and brain. Data collection included clinical record review and family interview using standardized verbal autopsy. The full set of data was reviewed by local experts using a standardized process (Determination of Cause of Death) to identify all relevant conditions leading to death (causal chain), per WHO recommendations. For analysis we stratified neonatal death into 24-hours of birth, early (1-<7 days) and late (7-<28 days) neonatal deaths. We analyzed 1458 deaths, 41% occurring within 24-hours, 41% early and 18% late neonatal deaths. Leading underlying causes of death were complications of intrapartum events (31%), complications of prematurity (28%), infections (17%), respiratory disorders (11%), and congenital malformations (8%). In addition to the underlying cause, 62% of deaths had additional conditions and 14% had ≥3 other conditions in the causal chain. The most common causes considering the whole causal chain were infection (40%), prematurity (32%) and respiratory distress syndrome (28%). Common maternal conditions linked to neonatal death were maternal hypertension (10%), labour and delivery complications (8%), multiple gestation (7%), placental complications (6%) obstructed labour and chorioamnionitis (5%, each). CHAMPS’ findings showing the full causal chain of events that lead to death, in addition to maternal factors, highlights the complexities involved in each death along with the multiple opportunities for prevention. Highlighting improvements to prenatal and obstetric care and infection prevention are urgently needed in high-mortality settings.

## Introduction

The child is highly vulnerable during the neonatal period. In 2019, 2.4 million children globally died in the first month of life, equivalent to 6,700 deaths per day [1]. Of those, about a third of all neonatal deaths occurred within the first day of life, and close to three-quarters occurred within the first week of life [1].

While neonatal death rates have dropped by 52% globally (from 38 to 17 deaths per 1000 live births between 1990–2019), neonatal deaths still comprise 45% of all child deaths under 5 year of age [2]. Marked disparities in neonatal mortality exist between countries and regions. Sub-Saharan Africa (SSA) and South Asia had the highest neonatal mortality, 27 and 25 deaths per 1000 live-births, respectively, in 2019 [1, 3]. A child born in SSA or South Asia was 10 times more likely to die in the first month of life than a child born in a high-income country [1, 4].

Sustainable Development Goal (SDG) 3.2 aims to “end all preventable deaths under 5 years of age” by 2030, with all countries aiming to reduce neonatal mortality to less than 12 deaths per 1000 live births and under-5 deaths to less than 25 deaths per 1000 live births [5]. More specific cause of death (CoD) information could help target prevention measures and achieve these targets. Currently, causes of neonatal deaths in low-and-middle-income countries are mainly inferred from vital registration and limited verbal autopsy data [6]. In 2015, only 3% of under-5 childhood cause-specific mortality fractions (CSMF) were based on adequate vital registration data, primarily from high-income countries [7]. Also, CSMF are usually derived considering only the underlying medical condition that led to death; examining the entire chain of events leading to death, including immediate and antecedent medical events, might identify more opportunities for targeted interventions and thus for preventing deaths.

The gold standard for obtaining accurate causes of death information is a complete diagnostic autopsy, more often, the procedure is not even suggested as it is not typically feasible in LMICs because of the expense, required expertise and, in some settings many families decline the procedure for personal, socio-cultural, and religious reasons [8–11]. During the past decade, use of post-mortem specimens collected through minimally invasive tissue sampling (MITS) has shed light on the sequence of events leading to deaths in children [12, 13]. MITS allows post-mortem examination of critical tissues using histopathology, microbial culture, molecular detection, and other diagnostic testing such as serology and rapid tests [14].

The Child Health and Mortality Prevention Surveillance (CHAMPS) network’s mission is to generate scientific knowledge to save children’s lives by collecting, analyzing, and sharing accurate, timely data about the causes of child mortality in the regions where it is highest. In this manuscript, we describe conditions in the causal chain that led to neonatal deaths in our sites, including assessments of conditions for both newborns and their mothers. In addition, we evaluate whether such deaths were from preventable causes and, if so, how they might be addressed.

## Methods

The analysis included deaths enrolled in CHAMPS sites who had MITS collected and whose death occurred between December 2016 and December 2021. CHAMPS protocol and methods are described elsewhere (www.champshealth.org) [12, 15]. Briefly, enrollment occurred in seven countries: Bangladesh, Ethiopia, Kenya, Mali, Mozambique, Sierra Leone; and, South Africa [15].

Stillbirths and children under five years that were residing in catchment areas at the time of death were eligible for CHAMPS enrollment [16]. Parents or guardians were approached for written consent for the CHAMPS teams to conduct a standard verbal autopsy and clinical chart abstraction; for deaths identified within 24 hours (or 72 hours if refrigerated), consent was also sought for the MITS procedure. If parent were minor assent were approached from the parents and consent were approached from parent’s guardians. The MITS procedure included collection of tissue from liver, lungs, and brain (through posterior and transnasal approaches) and collection of peripheral blood, cerebrospinal fluid (CSF), stool, and oropharyngeal/nasopharyngeal (OP/NP) swabs [14]. Site laboratories tested post-mortem blood samples for Human Immunodeficiency Virus DNA or RNA using polymerase chain reaction (PCR), Tuberculosis using GeneXpert and malaria using thick and thin smears and rapid diagnostic assays. Blood and CSF underwent microbial culture. PCR for screening of up to 116 pathogens was performed at each site using four custom-designed syndromic TaqMan array cards (TAC; ThermoFisher Scientific, Waltham, MA, USA) [17]. Tissue specimens were examined locally using routine histopathological techniques and, if indicated by histopathological examination or TAC results, further diagnostic tests such as special stains, immunohistochemistry, and molecular testing targeting specific microorganisms were performed at the CHAMPS Central Pathology Laboratory at the US Centers for Disease Control and Prevention (CDC) Infectious Diseases Pathology Branch [18].

DeCoDe panels convened at each site and reviewed all post-mortem diagnostic test results, pathology findings, clinical abstraction information from child and maternal health records and VA responses [15, 19] for each death before assigning causes of death, taking into account all information, and using standard case definitions that include levels of diagnostic certainty [19] (available at https://champshealth.org/wp-content/uploads/2021/01/CHAMPS-Diagnosis-Standards.pdf). Level 1 was the most certain level, requiring the most evidence, and Level 3 the least certain. Panels determined each CoD and provided a causal chain [15, 19], shedding light on the sequence of events that led to the fatal outcome, using WHO ICD-10 and WHO application of ICD-10 for perinatal deaths (ICD-PM) guidelines [20, 21]. Panels included a diagnosis as a cause of death if the child may have survived if the condition had not occurred. For deaths in neonates attributed to a single condition, that condition was considered the underlying CoD. For deaths in which more than one condition played a role, underlying, antecedent, and immediate causes were assigned. The underlying cause occurred before immediate or comorbid conditions and may have predisposed the child to an immediate cause or co-morbid illnesses that then led to death; the immediate cause was closest to the death and the comorbid causes were in-between the underlying and immediate causes. We defined the causal chain to include all conditions listed as underlying, antecedent, and immediate causes of death. For neonatal deaths, the main maternal condition that contributed to the main or underlying condition in the neonate was also documented. For each death, the DeCoDe panel through expert consensus determined whether the death was preventable in the local context by considering the clinical, pathological, microbiological, and verbal autopsy information. The definition of preventability captures the conditions immediately surrounding the death of that particular child and not the broader political, financial, and societal influences. If the death was deemed preventable, the panel recommended health system improvements that could have prevented the death [22].

Ethics committees overseeing investigators at each site and at Emory University approved overall and site-specific protocols (Emory IRB#: 00091706). Protocols are available at: https://champshealth.org/resources/protocols.

Data analyses examined neonatal deaths for which CoD determinations were completed by a DeCoDe panel. Cases were stratified by age; death in the first 24-hours of life, early neonatal death (24-hours -<7 days; END), and late neonatal death (7–27 days; LND). Descriptive statistics and comparative chi-squared or fisher exact where appropriate were performed using Stata software version 16 (StataCorp, College Station, Texas).

## Results

**Fig 1:**
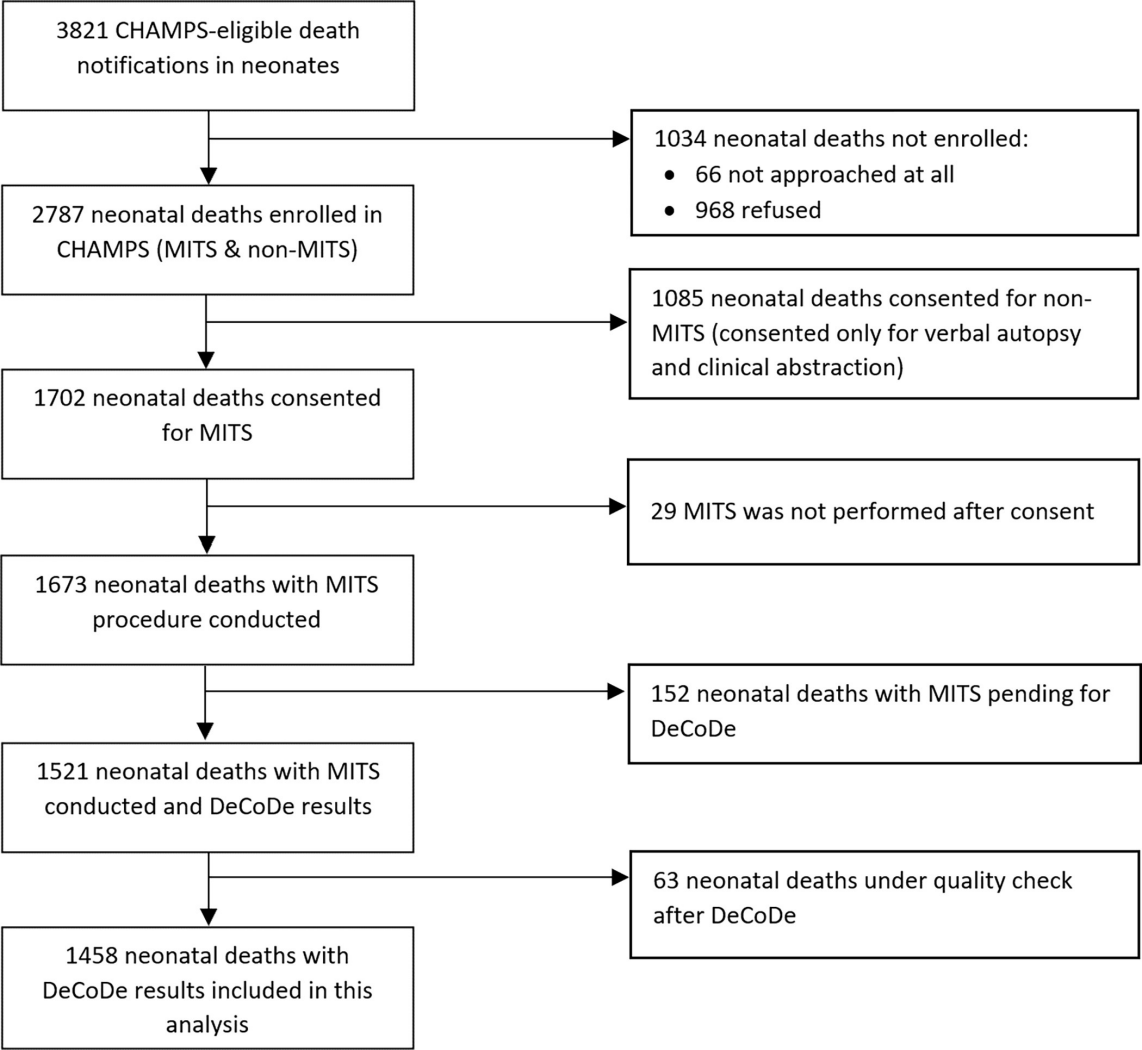
Flowchart of enrolled neonatal deaths from CHAMPS sites between December 2016 –December 2021, that had minimally invasive tissue samples (MITS) and consent only for verbal autopsy and clinical abstraction (Non-MITS) and included in the analysis.

Between December 2016 and December 2021, CHAMPS sites received 3821 CHAMPS eligible neonatal death notifications of which 2787 (73%) were enrolled in CHAMPS; of these, 61% (1702/2787) consented for MITS. MITS was conducted on 1673 (98%); 1458 (87%) had complete DeCoDe results available for this analysis (Fig 1). There is no difference in sex and age group distribution between MITS vs non-MITS cases, however more non-MITS deaths were from community (S1 Table). Of 1458 neonates, 31%, 21%, 12%, 12%, 10%, 8% and 5% were from South Africa, Mozambique, Kenya, Bangladesh, Sierra Leone, Ethiopia, and Mali, respectively (Table 1), with similar characteristics (S2 Table). The proportion of deaths occurring in the first 24-hours, the next 6 days and the subsequent 21 days was 41%, 41% and 18%, respectively (Table 1). Overall, 57% were male; median time between death and MITS collection was 12 hours (IQR 4, 21 hours).

**Table 1:** Characteristics of deaths enrolled in CHAMPS that occurred during the neonatal period, by age at death (2016–2021).

Characteristics	Total (N = 1,458)	Death in first 24 hours (N = 596)	Early Neonate (24 hours—<7 days) (N = 593)	Late Neonate (7–27 days) (N = 269)
Median (range) age at death, in days	2 (0, 5)	0 (0, 1)	3 (2, 4)	11 (9, 17)
Gender, n (%)				
Female	620 (42.5)	240 (40.3)	260 (43.8)	120 (44.6)
Male	835 (57.3)	353 (59.2)	333 (56.2)	149 (55.4)
Indeterminate	3 (0.2)	3 (0.5)	0 (0)	0 (0)
HIV Status, n (%)				
HIV infected	7 (0.5)	1 (0.2)	6 (1.0)	0 (0)
HIV exposed uninfected	285 (19.5)	94 (15.8)	115 (19.4)	76 (28.3)
HIV uninfected	1166 (80.0)	501 (84.1)	472 (79.6)	193 (71.7)
Mode of delivery, n (%)				
Normal vaginal delivery	875 (60.0)	367 (61.6)	346 (58.3)	162 (60.2)
Cesarean section	397 (27.2)	161 (27.0)	174 (29.3)	62 (23.0)
Instrumentation (e.g., forceps)	1 (0.1)	1 (0.2)	0 (0)	0 (0)
Unknown	185 (12.7)	67 (11.2)	73 (12.3)	45 (16.7)
Gestational age, n (%)				
≤ 28 weeks	278 (19.1)	111 (18.6)	108 (18.2)	59 (21.9)
28–33 weeks	225 (15.4)	73 (12.2)	94 (15.9)	58 (21.6)
34–36 weeks	130 (8.9)	43 (7.2)	59 (9.9)	28 (10.4)
37–42 weeks	348 (23.9)	142 (23.8)	150 (25.3)	56 (20.8)
unknown	477 (32.7)	227 (38.1)	182 (30.7)	68 (25.3)
Birth weight, n (%)				
Extremely low birth weight (<1000 gm)	249 (17.1)	109 (18.3)	98 (16.5)	42 (15.6)
Very low birth weight (1000–1499 gm)	248 (17.0)	81 (13.6)	97 (16.4)	70 (26.0)
Low birth weight (1500–2499 gm)	297 (20.4)	112 (18.8)	137 (23.1)	48 (17.8)
Normal weight (2500–4000 gm)	459 (31.5)	204 (34.2)	183 (30.9)	72 (26.8)
Macrosomia (>4000)	12 (0.8)	6 (1.0)	4 (0.7)	2 (0.7)
Missing	193 (13.2)	84 (14.1)	74 (12.5)	35 (13.0)
Median (range) weight at MITS*, in grams	1870 (1100, 2800)	2028 (1070, 2900)	1830 (1140, 2750)	1700 (1139, 2643)
Median (range) of hours between death and MITS done	12 (4, 21)	11 (4, 20)	12 (4, 20)	15 (7, 24)
Location of birth, n (%)				
Hospital	862 (59.1)	339 (56.9)	361 (60.9)	162 (60.2)
Health Centre	165 (11.3)	52 (8.7)	74 (12.5)	39 (14.5)
Health Post	12 (0.8)	8 (1.3)	2 (0.3)	2 (0.7)
Home	54 (3.7)	15 (2.5)	25 (4.2)	14 (5.2)
On the way to health center	13 (0.9)	6 (1.0)	4 (0.7)	3 (1.1)
Other location	10 (0.7)	2 (0.3)	4 (0.7)	4 (1.5)
Not recorded	342 (23.5)	174 (29.2)	123 (20.7)	45 (16.7)
Location of death, n (%)				
Community	77 (5.3)	29 (4.9)	20 (3.4)	28 (10.4)
Health facility	1381 (94.7)	567 (95.1)	573 (96.6)	241 (89.6)
Median (range) of hours in hospital**	34 (11, 101)	9 (4, 20)	54 (33, 96)	219 (157, 316)
CHAMPS sites				
Bangladesh	180 (12.3)	88 (14.8)	76 (12.8)	16 (5.9)
Ethiopia	110 (7.5)	58 (9.7)	41 (6.9)	11 (4.1)
Kenya	181 (12.4)	104 (17.4)	47 (7.9)	30 (11.2)
Mali	74 (5.1)	27 (4.5)	29 (4.9)	18 (6.7)
Mozambique	306 (21.0)	129 (21.6)	132 (22.3)	45 (16.7)
Sierra Leone	144 (9.9)	41 (6.9)	80 (13.5)	23 (8.6)
South Africa	463 (31.8)	149 (25.0)	188 (31.7)	126 (46.8)

*7 missing values

**210 missing values

One in five (20%, n = 290/1458) who were enrolled were born to women living with HIV, including 16% (n = 94) of deaths within 24-hours, 20% (n = 120) END, and 28% (n = 76) LND. Deaths in neonates from HIV-infected mothers were from South Africa (58%, n = 169), Mozambique (29%, n = 83), Kenya (10%, n = 30), Bangladesh (1%, n = 4), and Sierra Leone (1%, n = 4). Only seven neonatal deaths (1% [n = 7/1458] of all, 2% [n = 5/290] of those with known HIV exposure) had acquired HIV from their mother (two of the seven mothers were treated with ART; 5 had data documenting mode of delivery, of which one was born by C-section) (Table 1).

Most (63%, n = 794/1265 with available birth weights) neonatal deaths occurred in babies who weighed <2500 gm at birth, and 39% (n = 497) weighed <1500 gm (37% <1500 gm among deaths in the first 24-hours, 38% END, and 48% LND). Neonatal deaths occurring within the first 24-hours of life (210/512; 41%) were more likely to have a normal birth weight (≥2500 gm) than neonatal deaths occurring later (261/753; 35%), p = 0.024. Nearly all (95%) deaths occurred in health facilities (Table 1).

### Underlying causes of death

The DeCoDe panel assigned an underlying cause for all except 21 (1%) deaths in which a CoD could not be determined (Table 2). Overall, 11 different WHO ICD-PM categories were assigned for the underlying cause of the death (Table 2 and S3 and S5 Tables). The level of data supporting each underlying CoD diagnosis was strong; 82.3% (1161/1410) had level 1 evidence, 13.0% (183/1410) had level 2 evidence, and 4.7% (66/1410) had level 3 evidence. Strength of evidence was not provided for underlying CoD for 3.4% (48/1410) of deaths. The most common underlying CoD categories assigned were complications of intrapartum events (446/1458 [31%]), low birth weight (LBW)/prematurity complications (404/1458 [28%]), infections (254/1458 [17%]), respiratory disorders (159/1458 [11%]), and congenital malformations (118/1458 [8%]; [Table 2]). Diagnoses in the complications of intrapartum events group were intrauterine hypoxia (233/446, 52%) and birth asphyxia (207/446, 46%). Among underlying causes of deaths in the congenital malformation group, 18% (n = 21/118) had congenital malformations of the nervous system, including 17 with neural tube defects, and among those with respiratory disorders, 79% (125/159) had respiratory distress syndrome of the newborn and 16% (25/159) had neonatal aspiration of meconium (Table 2).

**Table 2:** Underlying causes of death for newborn deaths enrolled in CHAMPS, by age at death and WHO ICD 10 Perinatal Mortality (PM) category (2016–2021). Criteria used to define each cause available at: https://champshealth.org/wp-content/uploads/2021/01/CHAMPS-Diagnosis-Standards.pdf.

Underlying Cause of Death	ICD-10	Total (N = 1,458) n (%)	Death in first 24 hours (N = 596) n (%)	Early Neonate (24 hours—<7 days) (N = 593) n (%)	Late Neonate (7–27 days) (N = 269) n (%)
**Congenital malformations, deformations & chromosomal abnormalities N1**	**Q00-Q99**	**118 (8.1)**	**46 (7.7)**	**41 (6.9)**	**31 (11.5)**
Congenital malformation of nervous system	Q00-Q07	21 (1.4)	13 (2.2)	6 (1.0)	2 (0.7)
Congenital malformations of circulatory system	Q20-Q28	12 (0.8)	3 (0.5)	5 (0.8)	4 (1.5)
Congenital malformation of digestive system	Q38-Q45	5 (0.3)	0 (0)	2 (0.3)	3 (1.1)
Congenital malformation of urinary system	Q60-Q64	7 (0.5)	4 (0.7)	1 (0.2)	2 (0.7)
Congenital malformations & deformations of musculoskeletal system	Q65-Q79	29 (2.0)	9 (1.5)	13 (2.2)	7 (2.6)
Other congenital malformations	Q18, Q33, Q80-Q89, D48	24 (1.6)	12 (2.0)	7 (1.2)	5 (1.9)
Chromosomal abnormalities, not elsewhere classified	Q90-Q99	20 (1.4)	6 (1.0)	7 (1.2)	7 (2.6)
Congenital myopathies	G71.2	1 (0.1)	0 (0)	0 (0)	1 (0.4)
**Disorders related to fetal growth N2**	**P05**	**11 (0.8)**	**4 (0.7)**	**4 (0.7)**	**3 (1.1)**
**Birth trauma N3**	**P15**	**1 (0.1)**	**0 (0)**	**1 (0.2)**	**0 (0)**
**Complications of intrapartum events N4**	**P20, P21, P02, O14.2**	**446 (30.6)**	**252 (42.3)**	**176 (29.7)**	**18 (6.7)**
Intrauterine hypoxia	P20	233 (16.0)	143 (24.0)	81 (13.7)	9 (3.3)
Birth asphyxia	P21	207 (14.2)	104 (17.4)	94 (15.9)	9 (3.3)
Complications of placenta, cord and membranes	P02	5 (0.3)	5 (0.8)	0 (0)	0 (0)
Hemolysis, Elevated Liver enzymes and Low Platelets (HELLP) syndrome	O14.2	1 (0.1)	0 (0)	1 (0.2)	0 (0)
**Convulsion and disorder of cerebral status N5**	**P90-P96**	**16 (1.1)**	**6 (1.0)**	**8 (1.3)**	**2 (0.7)**
**Infections N6**	** **	**254 (17.4)**	**75 (12.6)**	**100 (16.9)**	**79 (29.4)**
Other gastroenteritis and colitis of infectious and unspecified origin	A09	2 (0.1)	0 (0)	1 (0.2)	1 (0.4)
Other bacterial diseases	A40, A41	6 (0.4)	2 (0.3)	1 (0.2)	3 (1.1)
Congenital syphilis	A50	7 (0.5)	3 (0.5)	4 (0.7)	0 (0)
Viral infection of central nervous system	A86	1 (0.1)	0 (0)	0 (0)	1 (0.4)
Viral infections characterized by skin and mucous membrane lesions	B00-B09	1 (0.1)	0 (0)	0 (0)	1 (0.4)
Human immunodeficiency virus	B20-B24	1 (0.1)	0 (0)	1 (0.2)	0 (0)
Other viral diseases	B33.8	1 (0.1)	0 (0)	0 (0)	1 (0.4)
Bacterial meningitis	G00, G03.9	5 (0.3)	1 (0.2)	1 (0.2)	3 (1.1)
Bacterial & viral pneumonia	J12, J15, J16, J18	28 (1.9)	3 (0.5)	9 (1.5)	16 (5.9)
Congenital pneumonia	P23	33 (2.3)	22 (3.7)	11 (1.9)	0 (0)
Infection related to perinatal period	P35-P39	166 (11.4)	43 (7.2)	72 (12.1)	51 (19.0)
COVID-19	U07.1	2 (0.1)	0 (0)	0 (0)	2 (0.7)
Other infectious or parasitic diseases	P00.2	1 (0.1)	1 (0.2)	0 (0)	0 (0)
**Respiratory and cardiovascular disorders N7**	**P20-P29**	**159 (10.9)**	**80 (13.4)**	**66 (11.1)**	**13 (4.8)**
Respiratory distress syndrome of newborn	P22	125 (8.6)	62 (10.4)	55 (9.3)	8 (3.0)
Neonatal aspiration syndromes	P24	30 (2.1)	16 (2.7)	10 (1.7)	4 (1.5)
Pulmonary haemorrhage originating in the perinatal period	P26	3 (0.2)	1 (0.2)	1 (0.2)	1 (0.4)
Other respiratory conditions originating in the perinatal period	P28	1 (0.1)	1 (0.2)	0 (0)	0 (0)
**Other neonatal conditions N8**	**E41,E87.0, E88.9, K76.9,P03.4,P51.0,P52.4,P55.1,P55.9,P56.9,P57.9,P59.0,P59.2,P70.1,P76.9,P77,P83.2,P83.3,R10,R62.8**	**25 (1.7)**	**8 (1.3)**	**7 (1.2)**	**10 (3.7)**
**Low birth weight/prematurity complications N9**	**P07**	**404 (27.7)**	**116 (19.5)**	**178 (30.0)**	**110 (40.9)**
Low birth weight	P07.0-P07.1	353 (24.2)	97 (16.3)	152 (25.6)	104 (38.7)
Prematurity	P07.2-P07.3	51 (3.5)	19 (3.2)	26 (4.4)	6 (2.2)
**Miscellaneous N10**	**T71, W75, G93.4**	**3 (0.2)**	**0 (0)**	**2 (0.3)**	**1 (0.4)**
**Unspecified condition N11**	**R99**	**21 (1.4)**	**9 (1.5)**	**10 (1.7)**	**2 (0.7)**

**Fig 2:**
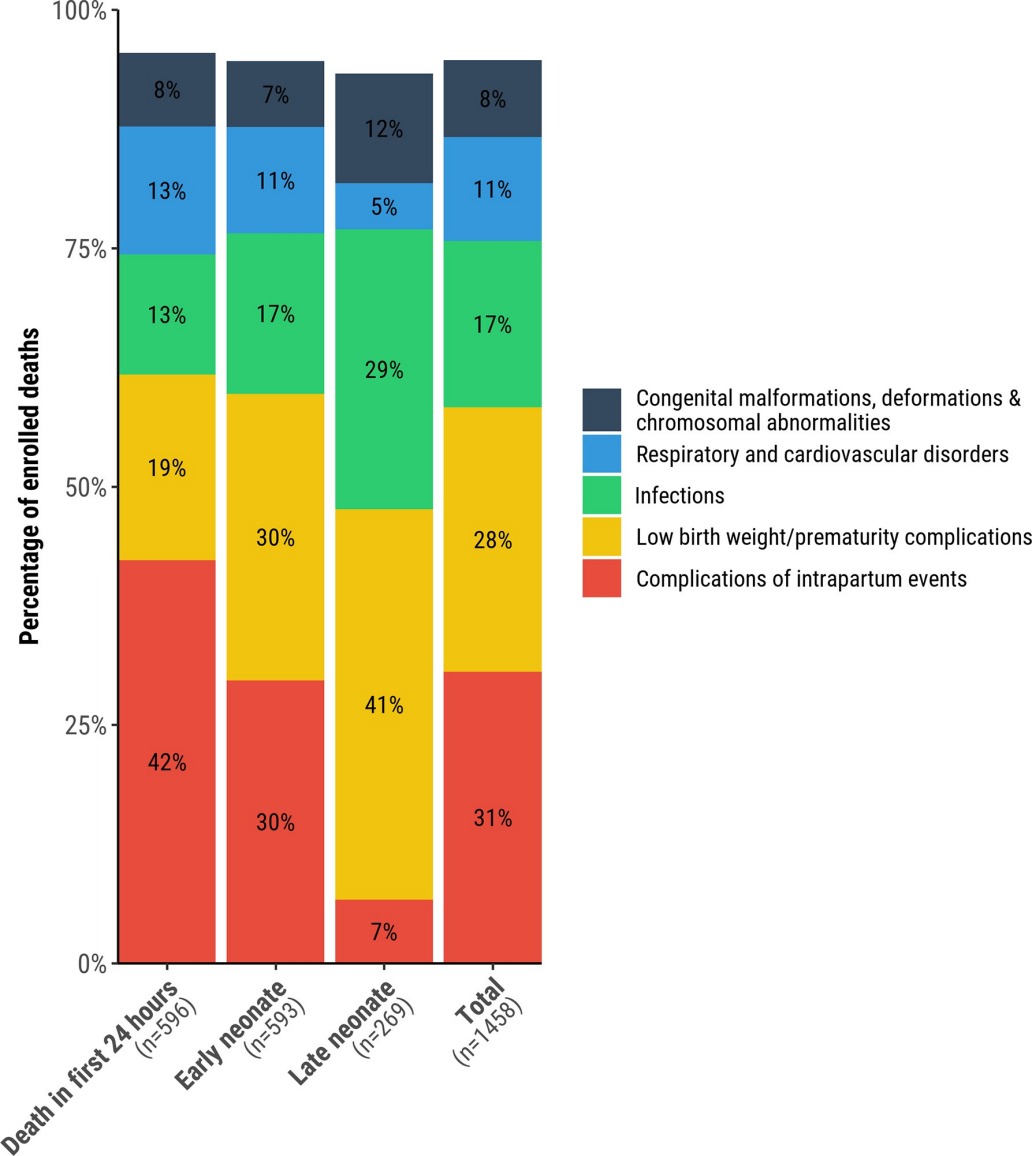
Relative proportion of neonatal deaths enrolled in CHAMPS, 2017–2021, attributed to the 5 most common categories of underlying causes (n = 826), overall and for deaths in the first 24 hours of life, early neonatal period (1–6 days), and late neonatal period (7–27 days).

LBW/prematurity complications were more common as an underlying cause in END (178/593 [30%]) and LND (110/269 [41%]) than in deaths in the first 24-hours (116/596 [20%]); infections and congenital malformations were found more often among underlying causes of death in LND (79/269 [29%] and 31/269 [12%]) compared to END (100/593 [17%] and 41/593 [7%]) and deaths in the first 24-hours of life (75/596 [13%] and 46/596 [8%]), respectively. In contrast, complications of intrapartum events were more common as underlying causes among deaths in the first 24-hours of life (252/596 [42%] compared to END (176/593 [30%] and LND (18/269 [7%]), respectively (Table 2, Fig 2).

Among HIV exposed neonate prematurity (39.3%, 114/290) and infection (19.3%, 56/290) was more prevalent underlying CoD as compared HIV unexposed neonates 24.8% (290/1168) and 17.0% (198/1168), respectively (S4 Table).

### Maternal conditions

Among all neonatal deaths, 60% (873/1458) were determined to be related to a maternal condition (Table 3). The category of ICD-PM classification of maternal conditions most identified was maternal medical and surgical conditions (M4) (233/1458, 16%; S6A Table). The most common specific conditions looking across the M1-M4 categories were maternal hypertension (10%, 149/1458), labour/delivery complications (8%, 112/1458), multiple gestation (7%, 99/1458), placental complications (6%, 84/1458), obstructed labour and fetal malpresentation (5%, 67/1458) and chorioamnionitis (3%, 48/1458) (S7 Table).

**Table 3:** Maternal conditions identified for CHAMPS deaths that occurred in the neonatal period, by WHO ICD 10 PM underlying cause of the death for the newborn (2016–2021).

Underlying cause of the death for the neonate	Number of neonatal deaths	Maternal condition					
		M1: Complications of Placenta, cord, and membranes	M2: Maternal complications of pregnancy	M3: Other complications of labour and delivery	M4: Maternal medical and surgical conditions	M5: No maternal condition	Other
	N = 1,458	N = 157 n (%)	N = 202 n (%)	N = 236 n (%)	N = 233 n (%)	N = 585 n (%)	N = 45 n (%)
N1—Congenital malformations, deformations, and chromosomal abnormalities	N = 118	2 (1.3)	2 (1.0)	6 (2.5)	5 (2.1)	99 (16.9)	4 (8.9)
N2—Disorders related to fetal growth	N = 11	1 (0.6)	2 (1.0)	1 (0.4)	5 (2.1)	2 (0.3)	0 (0)
N3—Birth trauma	N = 1	0 (0)	0 (0)	0 (0)	0 (0)	1 (0.2)	0 (0)
N4—Complications of intrapartum events	N = 446	52 (33.1)	38 (18.8)	143 (60.6)	61 (26.2)	146 (25.0)	6 (13.3)
N5—Convulsions & disorders of cerebral status	N = 16	1 (0.6)	2 (1.0)	4 (1.7)	0 (0)	8 (1.4)	1 (2.2)
N6—Infection	N = 254	28 (17.8)	30 (14.9)	20 (8.5)	35 (15.0)	140 (23.9)	1 (2.2)
N7—Respiratory and cardiovascular disorders	N = 159	14 (8.9)	32 (15.8)	31 (13.1)	20 (8.6)	61 (10.4)	1 (2.2)
N8—Other neonatal conditions	N = 25	1 (0.6)	1 (0.5)	0 (0)	2 (0.9)	21 (3.6)	0 (0)
N9—Low birth weight and prematurity	N = 404	57 (36.3)	95 (47.0)	31 (13.1)	104 (44.6)	85 (14.5)	32 (71.1)
N10—Miscellaneous	N = 3	0 (0)	0 (0)	0 (0)	0 (0)	3 (0.5)	0 (0)
N11—Neonatal death of unspecified cause	N = 21	1 (0.6)	0 (0)	0 (0)	1 (0.4)	19 (3.2)	0 (0)

Among neonates who had LBW/prematurity as their underlying cause of the death, 93% (378/404) had a maternal condition identified; most common were preterm labor or delivery (62%, 252/404), HIV (27%, 110/404), and maternal hypertension (22%, 89/404). Among neonates who had an intrapartum event as their underlying CoD, common maternal conditions were labour/delivery complications (23%, 102/446) and obstructed labour (16%, 71/446). Conversely, maternal conditions were only noted in 66% (170/254) of neonates who had an infection as underlying cause of the death, most common was preterm labor or delivery (17%, 44/254; Table 3A and S6B Table). Neonatal deaths that occurred close to birth more frequently had a maternal condition identified as contributing to the death. Among neonatal deaths in the first 24-hours of life, 72% (431/596) had a maternal condition determined to cause the death; most common were labour/delivery complication (12%, 73/596) and maternal hypertension (8%, 49/596). Among END and LND, 61% (359/593) and 43% (117/269) had a maternal condition listed, respectively. In both age groups, the common was maternal hypertension, 13% (76/593) and 9% (24/269), respectively (S7 Table and S1 Fig).

### Number and types of antecedent and immediate causes of death

DeCoDe panels determined that at least one other (antecedent/immediate) neonatal condition was responsible in addition to the underlying cause for 62% of deaths; 14% had 3 or more other conditions in the causal chain (Table 4). When the underlying cause was LBW/prematurity, 95% of neonatal deaths had at least one antecedent/immediate condition in addition to the underlying cause in the causal chain, and 33% had 3 or more other conditions. When the underlying cause of the death was infection, 53% of deaths had at least one other cause of the death. In contrast, for neonates with complications of an intrapartum event as the underlying cause of the death, little more than half (52%) did not have other causes of death, and only 4% had 3 or more other (antecedent/immediate) causes. Multiple conditions in the causal chain were more common among LND (77%) and END (65%) compared to deaths in the first 24-hours (45%, p<0.001 vs late and END combined; S2 Fig). When we excluded deaths from South Africa, the site with most capacity to provide neonatal intensive care, multiple conditions remained more common among deaths after the first 24-hours (59% among LND and 52% among END versus 36% among deaths in the first 24-hours, *P*<0.001).

**Table 4:** Number of immediate and antecedent conditions in the causal chain for CHAMPS deaths that occurred in the neonatal period, by each WHO ICD 10 PM underlying cause of the death (2016–2021).

Underlying cause of death	Number of immediate & antecedent causes of death in causal chain				
	N = 1,458	0	1	2	3+
		553 (37.9)	442 (30.3)	255 (17.5)	208 (14.3)
N1—Congenital malformations, deformations, and chromosomal abnormalities	N = 118	30 (25.4)	54 (45.8)	18 (15.3)	16 (13.6)
N2—Disorders related to fetal growth	N = 11	0 (0)	6 (54.5)	3 (27.3)	2 (18.2)
N3—Birth trauma	N = 1	0 (0)	0 (0)	1 (100.0)	0 (0)
N4—Complications of intrapartum events	N = 446	233 (52.2)	137 (30.7)	57 (12.8)	19 (4.3)
N5—Convulsions & disorders of cerebral status	N = 16	11 (68.8)	4 (25.0)	1 (6.2)	0 (0)
N6—Infection	N = 254	119 (46.9)	73 (28.7)	36 (14.2)	26 (10.2)
N7—Respiratory and cardiovascular disorders	N = 159	110 (69.2)	27 (17.0)	12 (7.5)	10 (6.3)
N8—Other neonatal conditions	N = 25	8 (32.0)	11 (44.0)	5 (20.0)	1 (4.0)
N9—Low birth weight and prematurity	N = 404	19 (4.7)	129 (31.9)	122 (30.2)	134 (33.2)
N10—Miscellaneous	N = 3	2 (66.7)	1 (33.3)	0 (0)	0 (0)
N11—Neonatal death of unspecified cause	N = 21	21 (100.0)	0 (0)	0 (0)	0 (0)

**Fig 3:**
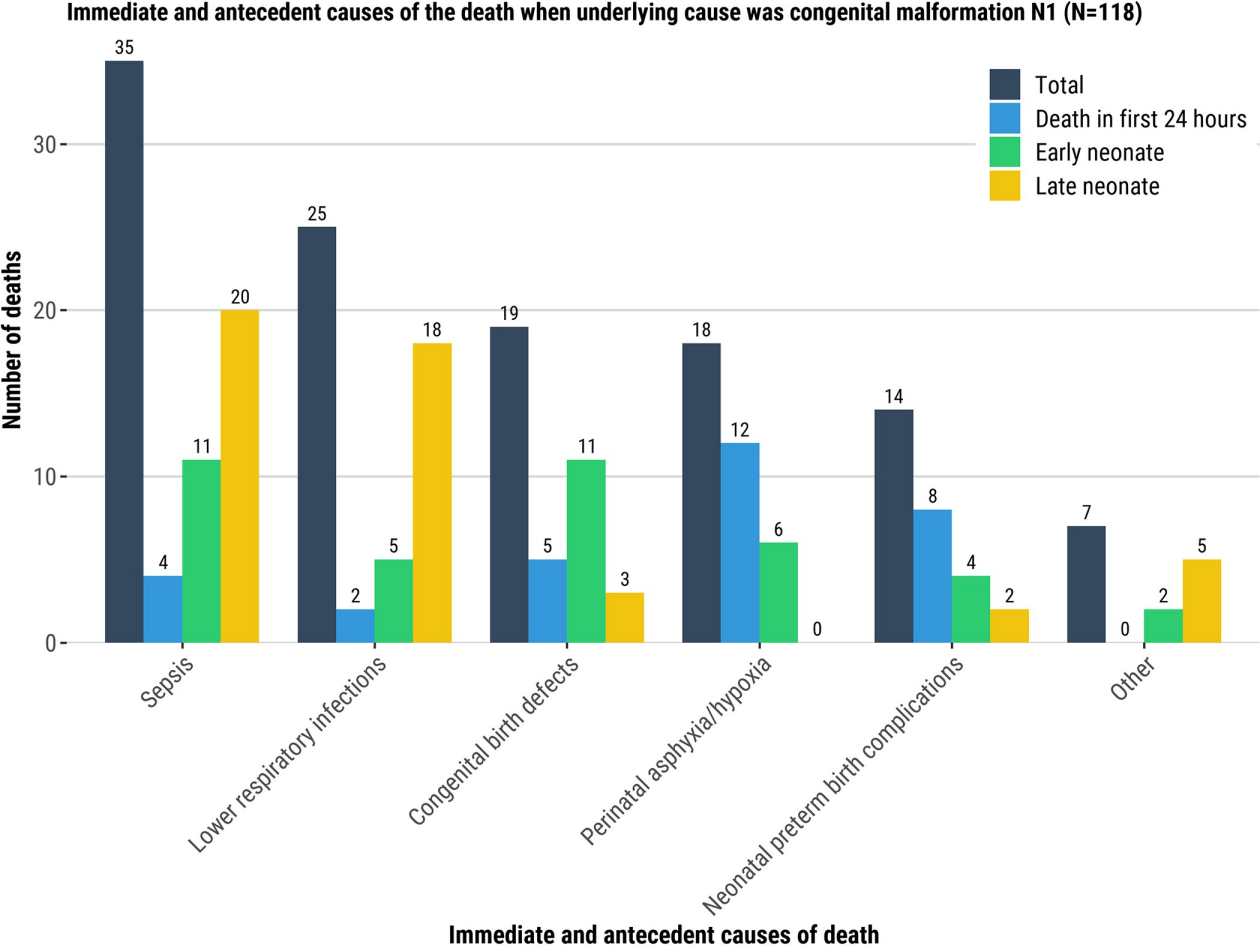
Immediate and antecedent causes of the death when the underlying cause of the death was: Congenital malformation (N1, Fig 3), complication of intrapartum event (N4, Fig 4), infection (N6, Fig 5), respiratory and cardiovascular disorder (N7, Fig 6), and low birth weight and prematurity (N9, Fig 7). There may be multiple immediate and antecedent causes of death for any individual, so the total number of causes may be greater than is listed in the figure titles.

**Fig 4:**
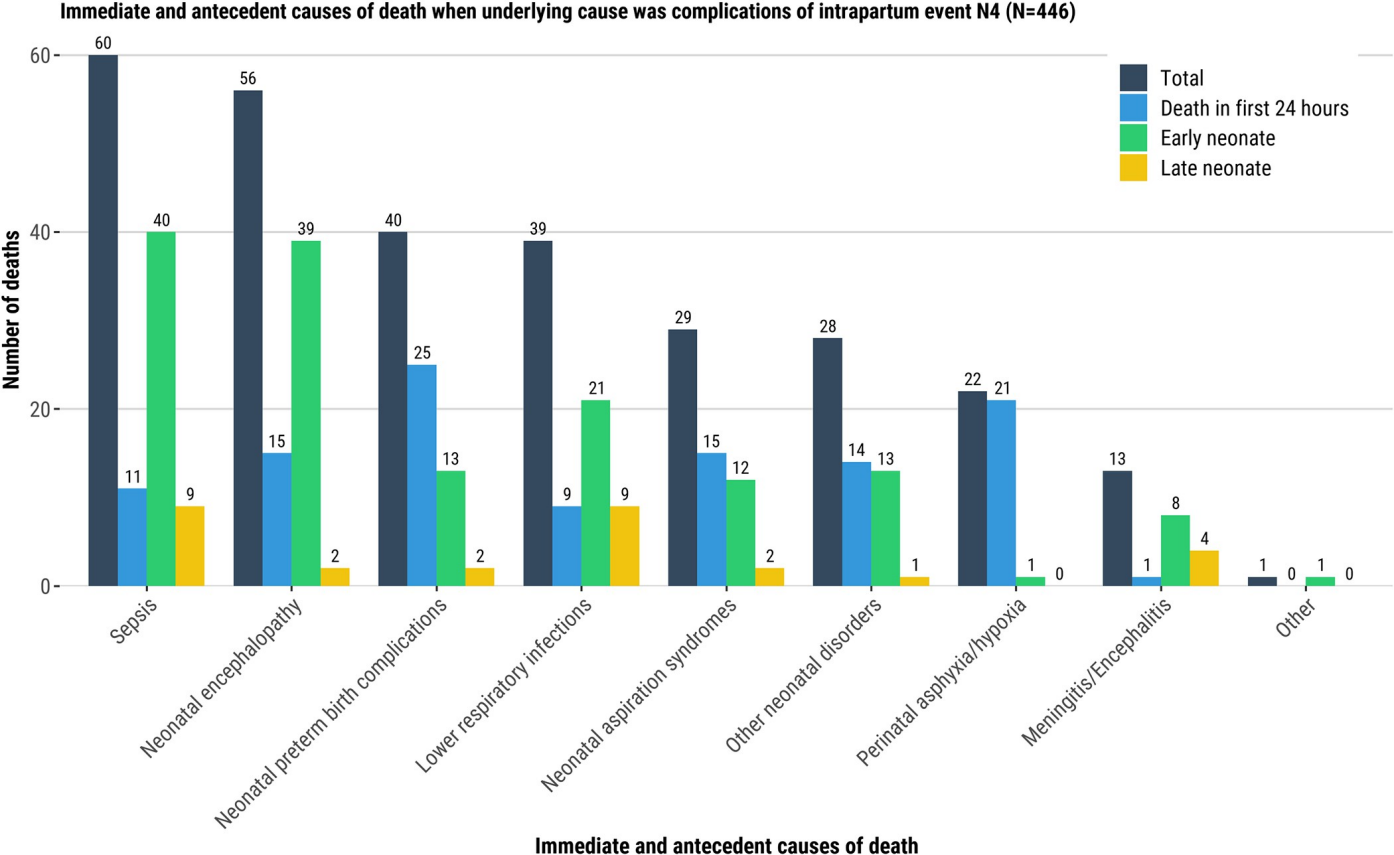
Immediate and antecedent causes of death when underlying cause was complications of intrapartum event N4 (N = 446). Immediate and antecedent causes of death.

Among neonatal deaths attributed to congenital malformation as the underlying cause of the death, neonatal sepsis and lower respiratory infection were the most common immediate and antecedent conditions in the causal chain, 30% (35/118) and 21% (25/118), respectively. Among deaths in the first 24-hours of life who had congenital abnormality as the underlying cause of death, perinatal hypoxia (26%, 12/46) and neonatal preterm birth complications (17%, 8/46) were the most common immediate and antecedent conditions (Fig 3). Infections (sepsis [20/31, 65%], lower respiratory infection [18/31, 58%], and meningitis [7/31, 23%]) were common among LND who had congenital abnormality as underlying cause of the death (Fig 3, S9 Table). When complications of an intrapartum event were the underlying cause of the death, the most common immediate and antecedent condition was neonatal encephalopathy (56/446, 13%), which was more predominant among END (39/176, 22%) than in the other age groups who had complication of intrapartum event as underlying cause of the death (Fig 4, S9 Table).

**Fig 5:**
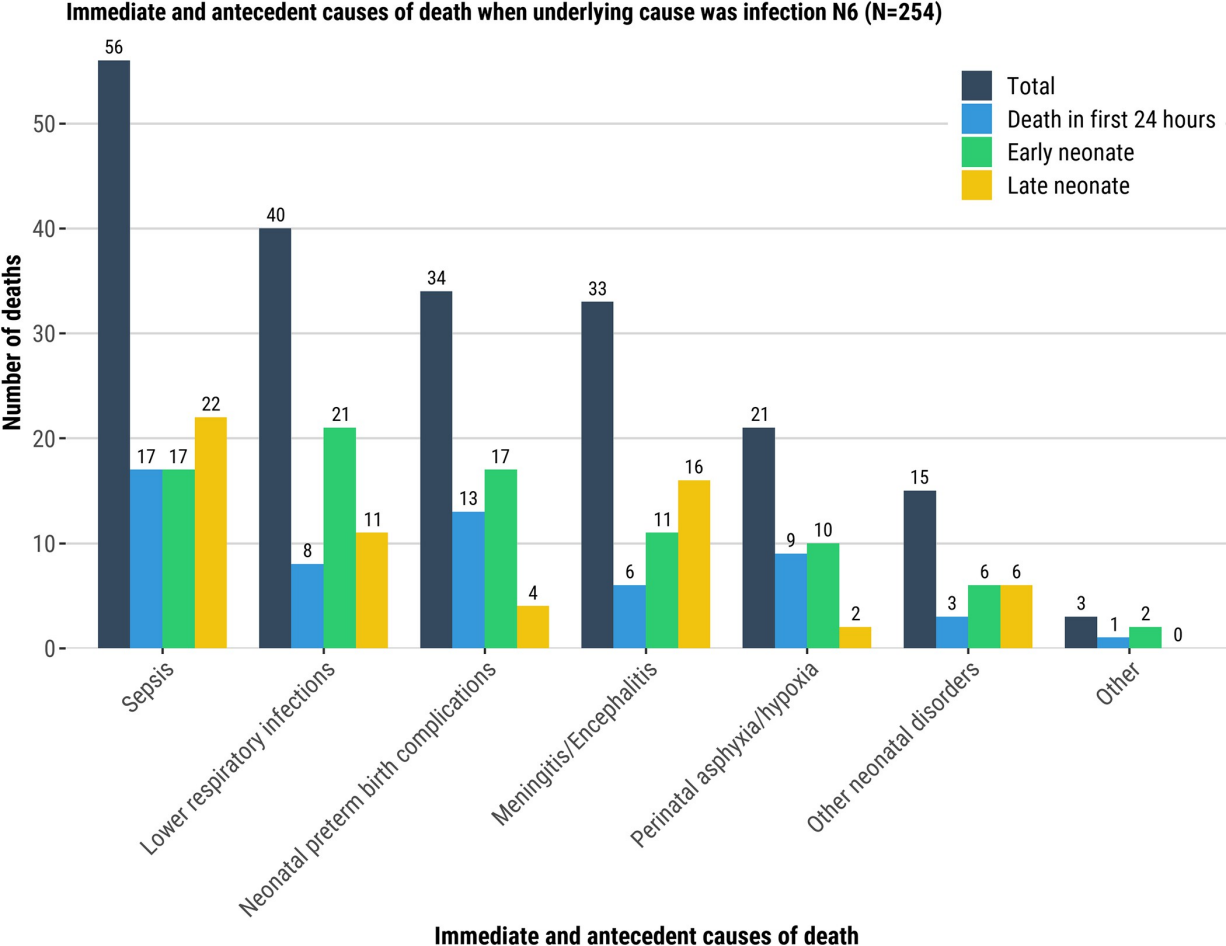
Immediate and antecedent causes of death when underlying cause was infection N6 (N = 254). Immediate and antecedent causes of death.

**Fig 6:**
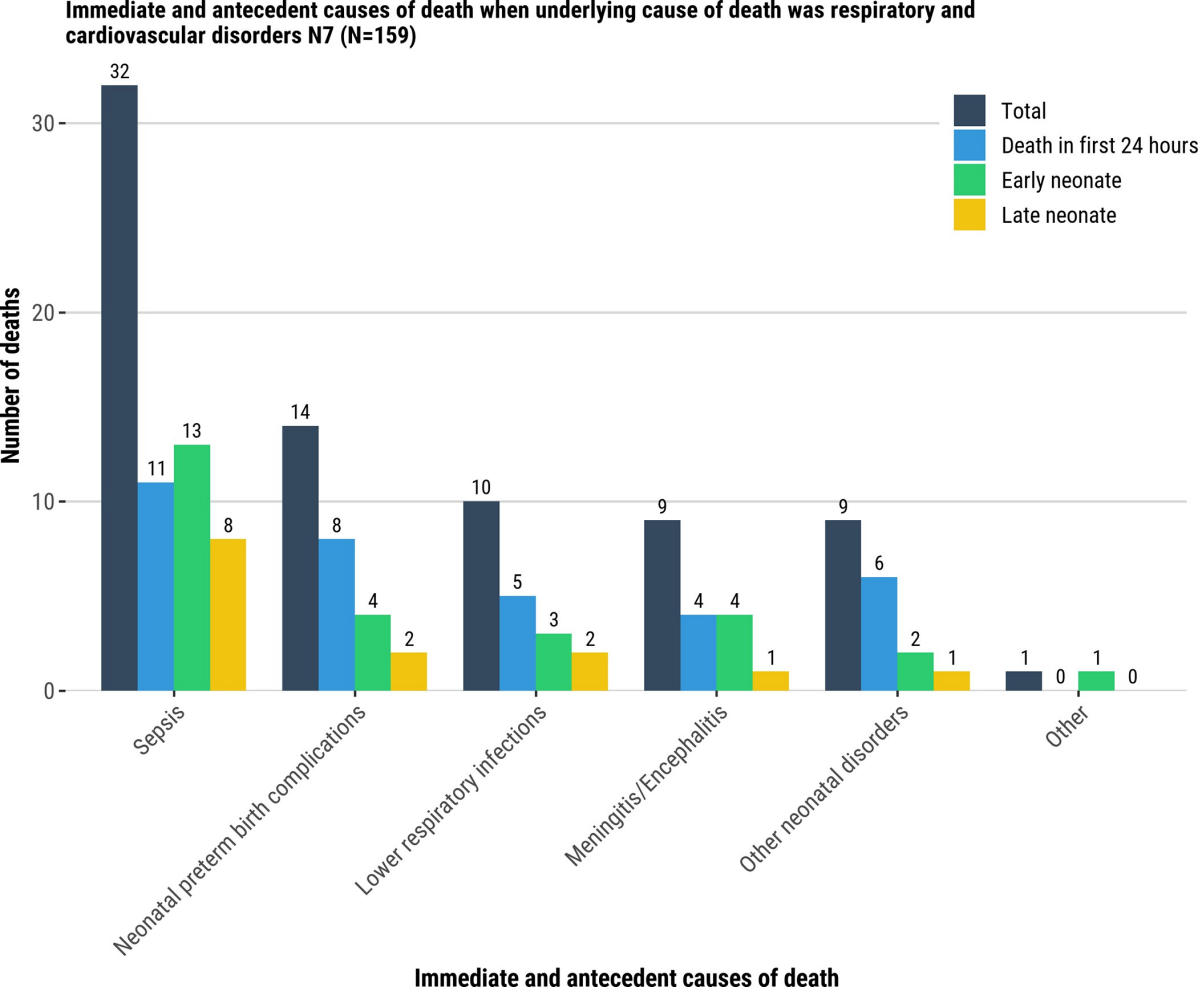
Immediate and antecedent causes of death when underlying cause of death was respiratory and cardiovascular disorders N7 (N = 159). Immediate and antecedent causes of death.

**Fig 7:**
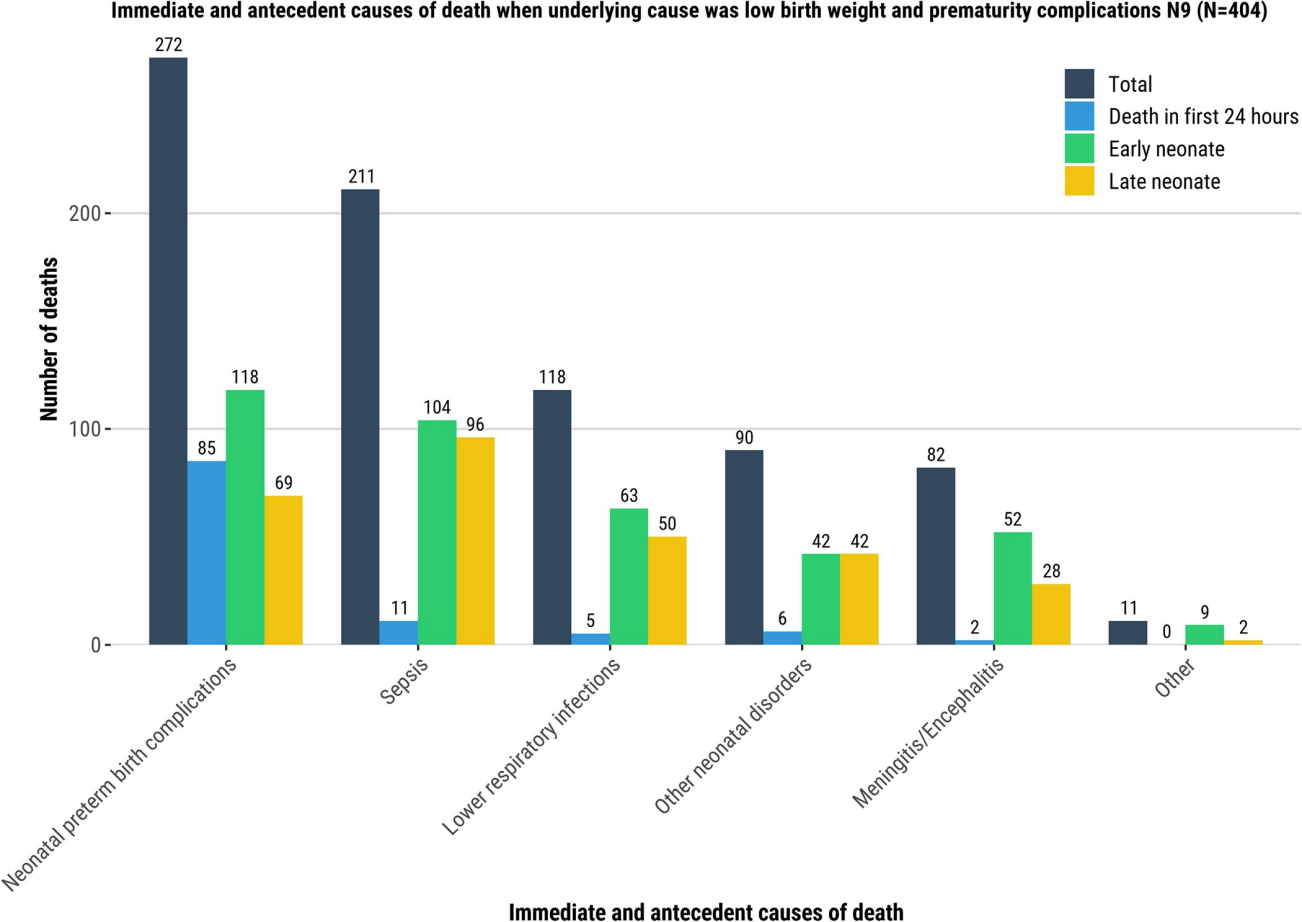
Immediate and antecedent causes of death when underlying cause was low birth weight and prematurity complications N9 (N = 404). Immediate and antecedent causes of death.

Among deaths attributed to an infection as the underlying cause, neonatal sepsis and lower respiratory infection were most common among immediate and antecedent conditions (56/254 [22%] and 40/254, [16%], respectively; [Fig 5, S9 Table]). When respiratory disorder was the underlying cause of the death, neonatal sepsis was most common immediate and antecedent condition (32/159 [20%]; Fig 6, S9 Table). Among deaths attributed to LBW/prematurity complications as the underlying cause, neonatal preterm birth complications were the most common immediate and antecedent condition in the causal chain (272/404, 67%) overall; among LND with LBW/prematurity complications as the underlying cause, sepsis (96/110, 87%) was an immediate/antecedent condition for nearly all cases (Fig 7, S9 Table).

The most common causes of death, including underlying, antecedent, and immediate causes, were LBW (473 deaths, 32%; S8 Table) and respiratory distress syndrome (412 deaths, 28%); 273 (19%) deaths had both LBW and respiratory distress in the causal chain. Deaths attributed to LBW were more common among END (205/593, 35%) and LND (118/269, 44%) as compared to deaths in the first 24-hours (150/596, 25%), *p*<0.001 vs LND and END combined. Among neonatal deaths that did not have LBW in the causal chain (n = 985), the most common underlying causes of death were intrapartum events (n = 426, 43%), infection (n = 225, 23%), and congenital malformation (n = 111, 11%). Intraventricular hemorrhage was found in 103 deaths; for 60 of these deaths, intraventricular hemorrhage was determined to be a cause of death.

**Fig 8:**
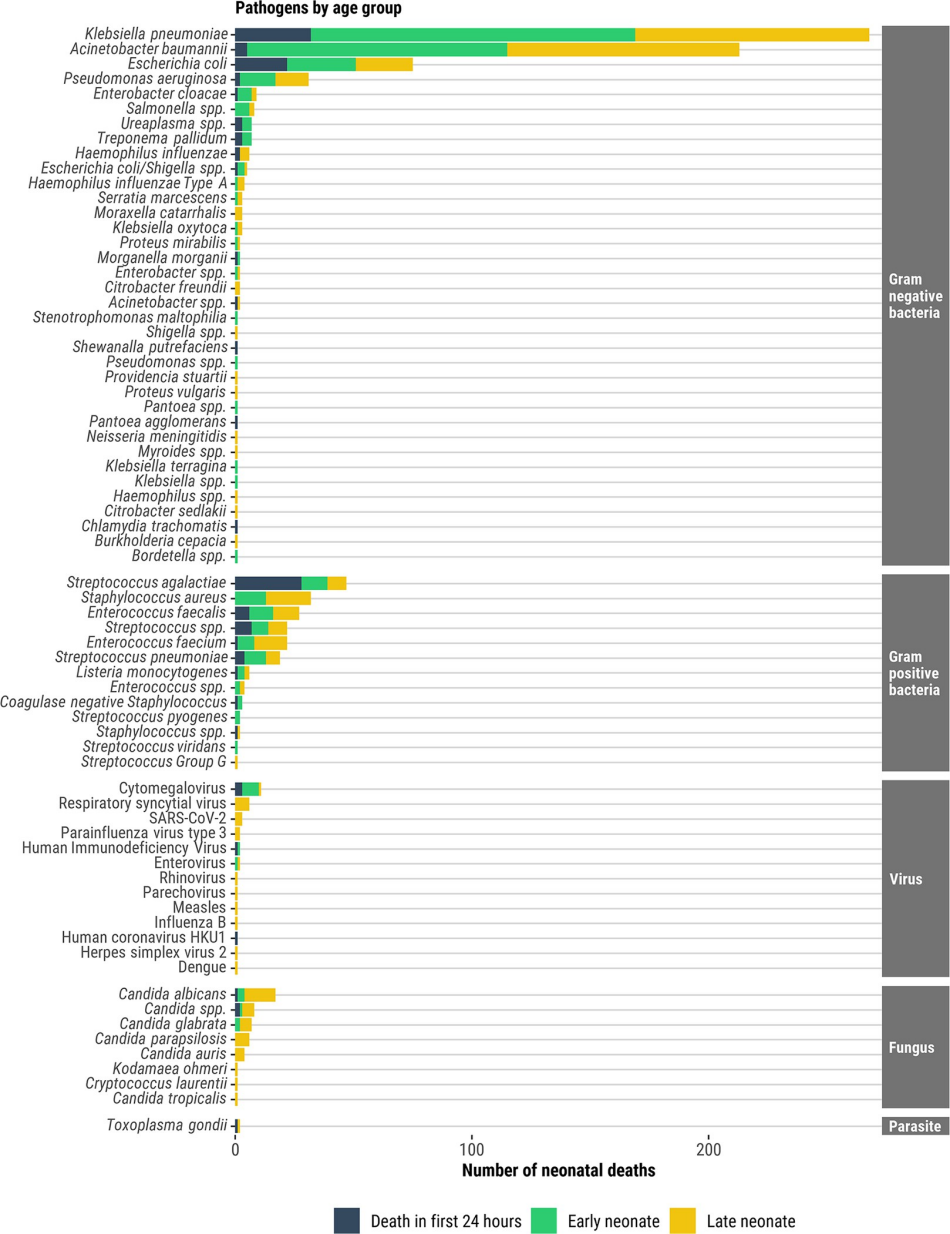
Pathogens identified in neonatal deaths in which infection was determined in the causal pathway, by age at death.

Just under half (590/1458, 40%) of the neonatal deaths had an infectious syndrome in the causal chain, either sepsis (504/590, 85%), pneumonia (254/590, 43%), meningitis (143/590, 24%) and /or other infections (33/590, 6%); note that the sum of infectious syndromes is >590 because the majority of deaths had >1 infectious syndrome in the causal pathway (S10 Table). The most common Gram-negative bacterial pathogens were *Klebsiella pneumoniae* (*K*. *pneumoniae*; 45% (268/590) of all infectious deaths), *Acinetobacter baumannii* (*A*. *baumannii*; 36%, 213/590), and *Escherichia coli* (*E*. *coli*; 13%, 75/590). *K*. *pneumoniae* and *A*. *baumannii* were more common among END (50% [137/272] and 40% [110/272]; respectively) and LND (46% [99/214] and 46% [98/214], respectively), than among deaths in the first 24-hours (Fig 4 and S10 Table). Most (79%, 168/213) *A*. *baumannii* infections were identified in the South Africa site, mainly driven by hospital-acquired infections. *E*. *coli* infections were more common among infectious deaths in the first 24-hours compared to END and LND, 21% (22/104) vs 11% (29/272) and 11% (24/214), respectively. *Streptococcus agalactiae* (Group B streptococcus) and *Staphylococcus aureus* were most common among Gram-positive bacteria, accounting for 8% (47/590) and 5% (32/590), respectively, of all infectious deaths. *Streptococcus agalactiae* was more common among infectious deaths in the first 24-hours compared to END and LND, 27% (28/104) vs 4% (11/272) and 4% (8/214), respectively. *Staphylococcus aureus* was only found among END (5%, 13/272) and LND (9%, 19/214). Only 33 (6%) deaths had viruses and 43 (7%) had fungi noted as causing death among those who had infectious conditions in the causal chain (Fig 8 and S10 Table).

### Assessment of preventability

**Fig 9:**
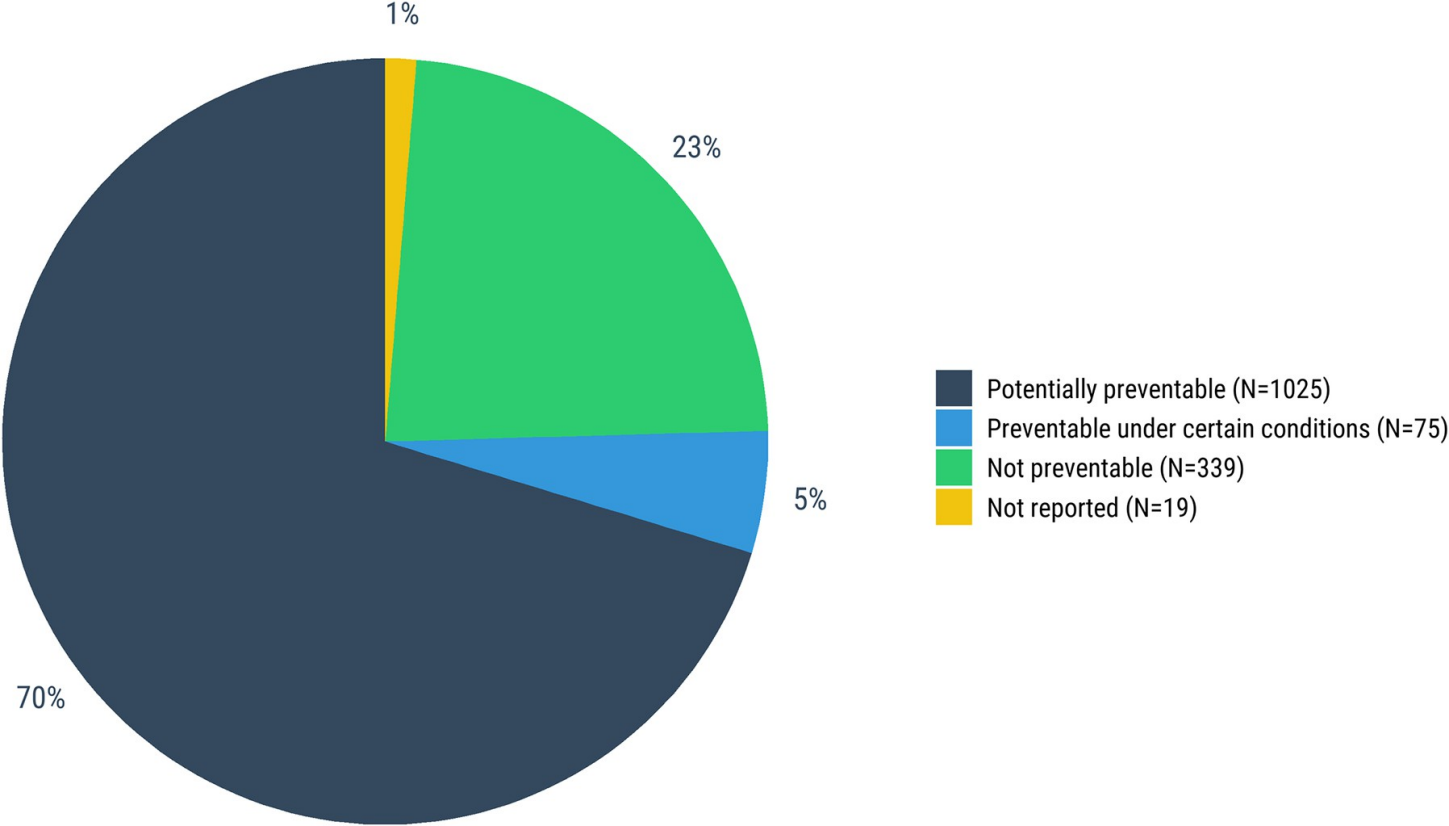
Expert (DeCoDe) panel determination of whether neonatal deaths were preventable (Fig 9) and recommended improvements that could prevent such deaths (Fig 10).

**Fig 10:**
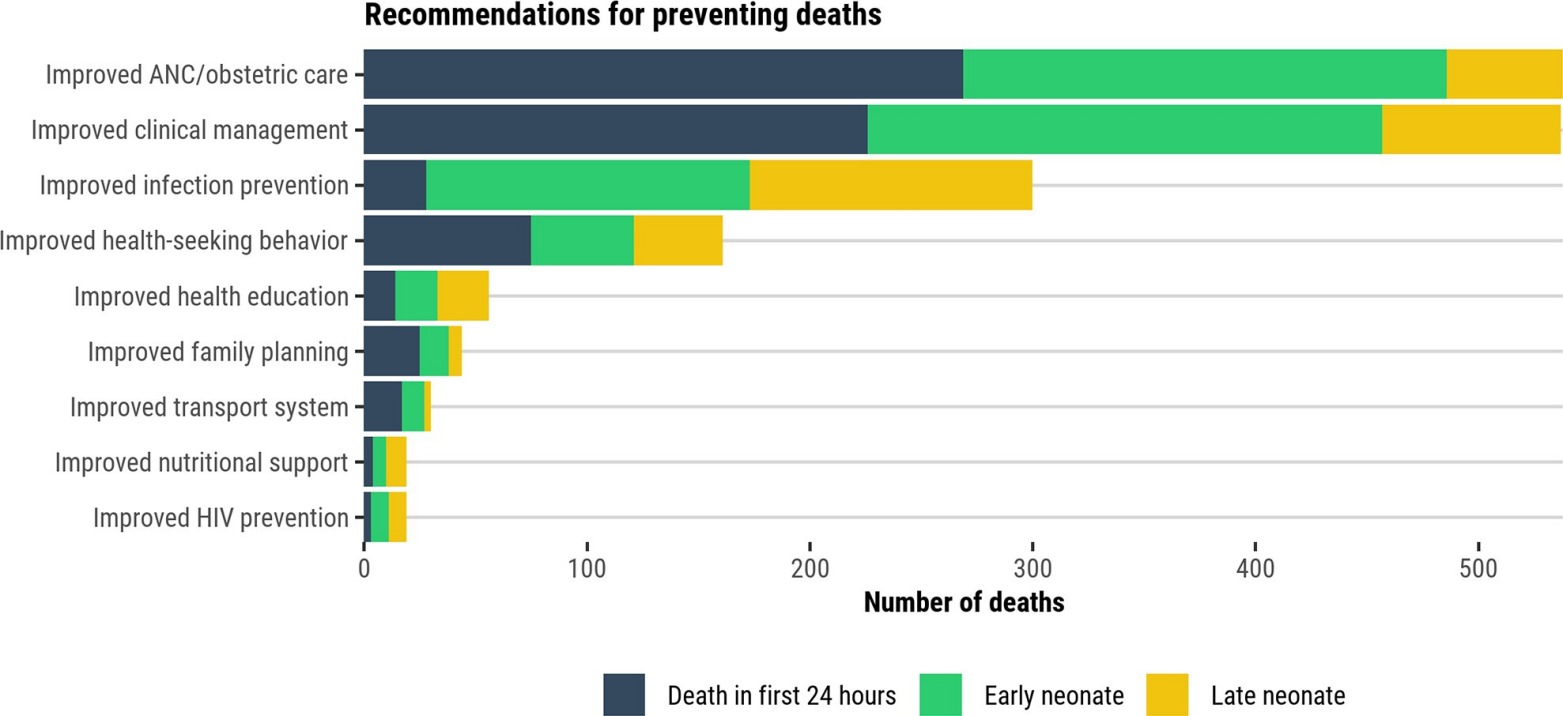
Recommendations for preventing deaths.

DeCoDe panels determined that three-fourths (75%, 1100/1458) of all neonatal deaths were potentially preventable or preventable under certain conditions; 72% (428/596) of deaths in the first 24-hours, 75% (447/593) of END, and 84% (225/269) of LND (Fig 9, S11A Table). Recommendations for how to prevent similar deaths in the future were available for 91% (997/1100) of all preventable deaths. These recommendations most often included improved clinical management and quality of care for neonates (49%, 537/1100), improved antenatal and obstetric care and management (49%, 538/1100), and improved infection prevention and control (27%, 300/1100; Fig 10, S11B Table). Improved antenatal and obstetric care and management were more relevant to deaths in the first 24-hours (63%, 269/428) and improved clinical management and quality of care to END (52%, 231/447). Infection prevention and control were more relevant for LND (56%, 127/225). Among the 339 deaths that were deemed unpreventable, the most common causes were preterm birth complications (n = 165), sepsis (n = 93), congenital birth defects (n = 84), and perinatal asphyxia/hypoxia (n = 62).

## Discussion

Our investigation of causes of death from 1458 neonates in high child mortality settings found that 5 main groups of causes (LBW, complications of intrapartum events, infections, congenital malformations, and respiratory disorders) accounted for 95% of underlying causes of death in this age group. Similar to CHAMPS findings, WHO figures state that 75% of neonatal deaths occur in the first-week of life (82% in CHAMPS), and preterm births, perinatal asphyxia, infections, and congenital defects are recognized as being the leading causes of neonatal deaths [1, 2]. Likewise, modeling by IHME estimates that preterm birth, encephalopathy due to birth asphyxia and trauma, and sepsis and other infections caused about three-fourths of disability adjusted life years lost among neonatal deaths in 2019 [23] and in recent publication it has been mentioned that birth asphyxia is under rated in LMICs [24]. CHAMPS methods add to existing reports by showing the full causal chain of events in newborns that lead to death, in addition to maternal factors, providing a clearer picture of the complexities involved in these deaths than studies or estimates that apply a single, underlying cause. Among those newborns who survive their first 24-hours, a large majority had more than one condition in the casual chain leading to death, which implies more opportunities and complexities to prevent such deaths [25]. In addition, we found that the most prevalent underlying causes of death shifted with the timing of the death; intrapartum events caused 42% of deaths in the first day of life but only 7% of neonatal deaths after the first week of life. Among those neonates who survived their first week but who died before reaching a month of life, most were LBW/preterm and many succumbed to infections that may have been linked to their hospital stays.

The young median age at death (2 days) among those who underwent MITS shows the disproportionate risk in the period immediately after birth, as has been reported in previous studies [26, 27]. During the first 24-hours of life, intrapartum complications that might have been preventable with better fetal monitoring and prompt intervention—such as birth asphyxia [26, 28, 29] and intrauterine hypoxia [30]—caused 42% of deaths [4]. Most newborns whose deaths were attributed to intrapartum complications had no other cause of death identified and most were normal birth weight; in other words, these babies would have been healthy babies if not for delivery complications. Three of four neonatal deaths occurring during the first 24-hours of life had a maternal condition noted, similar to findings from a study in Jordan [25] and further highlighting opportunities for identifying conditions that, had they been addressed, could have prevented deaths. Better delivery and availability of prenatal, intrapartum, and early post-partum care could prevent most of these deaths in the first day of life [22, 31]. Newborns who were determined to have multiple conditions in the causal pathway leading to death commonly had neonatal encephalopathy, a condition linked to delivery complications [32], among their causes of death.

Infections played an important role in the neonatal deaths that we examined in preterm/LBW newborns [33]. *Group B Streptococcus* and *E*. *coli* are known to be important causes of sepsis in the first days of life, and those pathogens were most common among deaths in the first 24-hours of life, consistent with *in utero* infection through vertical transmission. Our findings differ in some ways from those in the 3-country Aetiology of Neonatal Infections in South Asia (ANISA) study, which focused on community-acquired infections, used modelling to attribute causes of sepsis, and largely included newborns who survived their illnesses. In ANISA, neonatal sepsis was more often attributed to RSV and *Ureaplasma*, but they also found *K*. *pneumoniae*, *E*. *coli*, and other pathogens noted by CHAMPS. The CHAMPS study used much broader diagnostics and focused on neonates who died, resulting in a far higher proportion of subjects with identified infection by recognized sepsis pathogens. Gram-negative pathogens that are often resistant to first line antibiotics such as *K*. *pneumoniae* and *A*. *baumannii* were most common overall in CHAMPS and particularly among deaths after the first 24-hours. Most newborns who died from *K*. *pneumoniae* and *A*. *baumannii* likely acquired their infections after birth from the healthcare setting, given their prevalence among LNDs. Nearly 80% of *A*. *baumannii* infections were identified in the South Africa site and were mainly hospital-acquired infections. However, some of these infections caused deaths in the early neonatal period. The ANISA study also identified community-acquired, early onset infections attributed to *K*. *pneumoniae* and *A*. *baumannii*, suggesting the route of acquisition may not always be through a healthcare setting [34]. Preventing neonatal deaths through better infection prevention and control will be a challenge in facilities in LMICs as they gain greater capacity to provide supportive care to LBW newborns [33, 35]. In addition to improved clinical management and quality of care, healthcare systems in LMIC will need to address the lack of equipment and reduce the number of patients each provider must manage to improve survival of LBW newborns [22].

CHAMPS’ methods enable a better understanding of events that led to newborn deaths in high mortality areas, with causes of deaths confirmed by MITS [12]. Nonetheless, our methods do have some limitations. First, our surveillance teams are required to identify deaths within 24-hours (72-hours if refrigerated) so that MITS can be collected before burial or tissues start to degrade, 66 neonatal deaths that were not approached at all (shown in Fig 1), 28 (42%) were deaths in the first 24 hours, 28 (42%) were END, and 10 (15%) were LND. Such requirements mean that the deaths that are easiest to enroll, such as those that occur in health facilities, were overrepresented compared to deaths occurring in the community. This creates a bias by including only the deaths which could have a MITS completed and limits the generalizability, however the cause of death for MITS vs non-MITS is quite comparable (S3 Fig). Second, our study included broad health system improvement categories as recommendations for preventing deaths (e.g., improved clinical management and quality of care). The categories were developed from recommendations from the first DeCoDe panels and are being refined to be more specific. “Lastly, diversity among our study populations make our aggregate data more generalizable to high-mortality settings as a whole, although population characteristics might be different in each site. In particular, the main hospital in South Africa had more resources than facilities in other site, resulting in longer stays for low birth-weight newborns and more opportunity for exposure to hospital pathogens. In addition, each site’s catchment areas might not be the exact representative of the whole country.” How and whether the distribution of causes differs based on the location of death is unclear, although delivery complications might be more common among home deliveries and low and very LBW newborns are unlikely to survive if not brought to a healthcare setting [36]. Next, data on gestational age were not available for all deaths in our dataset; even when available the information may be inaccurate, as many pregnancies in CHAMPS sites are not evaluated with early ultrasound or other reliable dating methods. Designing interventions to prevent neonatal deaths will require more thorough examination of the specific challenges observed in each setting.

Our findings highlight the complexities and remaining opportunities for prevention of neonatal deaths. WHO through the World Health Assembly has sought commitment from countries to implement a global strategy for women’s, children’s and adolescent’s health, including an updated report on progress in 2021 [37]. The WHO/UNICEF *Every Newborn Action Plan* has four components designed to end all preventable stillbirths and neonatal deaths by 2030: 1) at least four prenatal care visits for pregnant women, 2) births attended by skilled health personnel, 3) early routine postnatal care, and 4) functional level 2 inpatient units for small and ill newborns [38]. Better implementation of these components may have prevented most of the deaths we identified, as most neonatal deaths were related to pregnancy or childbirth complications and occurred in the first 2 days of life, and many had documented maternal conditions contributing to death. As most of the neonatal deaths we enrolled occurred in health facilities, health personnel should have been available; whether personnel lacked equipment they needed or require more training are questions that need more investigation. Our findings also highlight the need for better infection prevention and control for low-birth-weight newborns as more level 1 and level 2 inpatient units become available.

## Supporting information

S1 Text Membership of CHAMPS Consortium. (DOCX)

S1 Table Characteristics of CHAMPS neonatal cases for whom MITS was performed versus all eligible neonatal deaths never enrolled in CHAMPS or consented to MITS (2017–2020). (DOCX)

S2 Table Characteristics of deaths enrolled in CHAMPS that occurred during the neonatal period, by age at death (2016–2021). (DOCX)

S3 Table WHO ICD10 Perinatal Mortality underlying cause of death categories & specific underlying cause of death by age group. (DOCX)

S4 Table Underlying cause of the death, stratified by HIV exposure status. (DOCX)

S5 Table Underlying causes of death for newborn deaths enrolled in CHAMPS, by site and WHO ICD 10 Perinatal Mortality (PM) category (2016–2021). (DOCX)

S6 Table a Main maternal condition attributed for neonatal deaths. b Maternal conditions identified for CHAMPS deaths that occurred in the neonatal period, by WHO ICD 10 PM underlying cause of the death for the newborn, 2016–2021. Bold text is groupings for underlying causes of death in neonates according to WHO ICD PM and Italic text shows the associated maternal conditions found in those deaths.
(ZIP)

S7 Table Main maternal condition attributed for neonatal deaths by age group. (DOCX)

S8 Table Neonatal deaths who had low birth weight in the causal pathway, by age at death. (DOCX)

S9 Table WHO ICD10 PM underlying cause of death and specific immediate or antecedent causes of death, overall and by age group. (DOCX)

S10 Table Pathogens identified in neonatal deaths in which infection was determined in the causal pathway, by age at death. (DOCX)

S11 Table a. Expert (DeCoDe) panel determination if neonatal deaths were preventable, overall and by age group. b. Recommended improvements that could prevent preventable deaths, overall and by age group.
(ZIP)

S1 Fig Main maternal condition attributed to neonatal death, by age at death (first 24 hours, early neonatal death [1–6 days], late neonatal death [7–27 days]). (DOCX)

S2 Fig Number of other conditions in the causal chain for CHAMPS deaths that occurred in the neonatal period, by age group. (DOCX)

S3 Fig Comparison of MITS vs non- MITS cause of deaths. (DOCX)

## References

[1] Organization WH. Newborns: improving survival and well-being Geniva: World Health Organization; 2020 [updated 18 Sep 2020]. Available from: https://www.who.int/news-room/fact-sheets/detail/newborns-reducing-mortality.

[2] Organization WH. Children: improving survival and well-being Geneva: World health organisation; 2020 [updated 8 Sep 2020; cited 2021]. Available from: https://www.who.int/news-room/fact-sheets/detail/children-reducing-mortality.

[3] Hug L, Alexander M, You D, Alkema L, for Child UI-aG. National, regional, and global levels and trends in neonatal mortality between 1990 and 2017, with scenario-based projections to 2030: a systematic analysis. The Lancet Global Health. 2019;7(6):e710–e20. 31097275 10.1016/S2214-109X(19)30163-9 PMC6527519

[4] EvaluatioN IfHMa. Infant and Child Mortality 15th Ave. NE, Seattle: Institute for Health Metrics and Evaluation. Available from: https://ourworldindata.org/child-mortality.

[5] Programme UND. sustainable develpment goals New York: United Nations Development Programme; [cited 2021 22 Apr 2021]. Available from: http://www.undp.org/content/undp/en/home/sustainable-development-goals/goal-3-good-health-and-well-being.html.

[6] Basera TJ, Schmitz K, Price J, Willcox M, Bosire EN, Ajuwon A, et al. Community surveillance and response to maternal and child deaths in low-and middle-income countries: A scoping review. PloS one. 2021;16(3):e0248143. doi: 10.1371/journal.pone.0248143 33725013 PMC7963102

[7] Liu L, Oza S, Hogan D, Chu Y, Perin J, Zhu J, et al. Global, regional, and national causes of under-5 mortality in 2000–15: an updated systematic analysis with implications for the Sustainable Development Goals. The Lancet. 2016;388(10063):3027–35. doi: 10.1016/S0140-6736(16)31593-8 27839855 PMC5161777

[8] Bunei M, Muturi P, Otiato F, Njuguna HN, Emukule GO, Otieno NA, et al. Factors Influencing Acceptance of Post-Mortem Examination of Children at a Tertiary Care Hospital in Nairobi, Kenya. Ann Glob Health. 2019;85(1). Epub 2019/07/06. doi: 10.5334/aogh.2504 ; PubMed Central PMCID: PMC6634467. 31276331 PMC6634467

[9] Blum LS, Karia FP, Msoka EF, Mwanga MO, Crump JA, Rubach MP. An In-Depth Examination of Reasons for Autopsy Acceptance and Refusal in Northern Tanzania. The American Journal of Tropical Medicine and Hygiene. 2020;103(4):1670–80. doi: 10.4269/ajtmh.20-0029 32748779 PMC7543794

[10] Maixenchs M, Anselmo R, Zielinski-Gutiérrez E, Odhiambo FO, Akello C, Ondire M, et al. Willingness to know the cause of death and hypothetical acceptability of the minimally invasive autopsy in six diverse African and Asian settings: a mixed methods socio-behavioural study. PLoS medicine. 2016;13(11):e1002172. doi: 10.1371/journal.pmed.1002172 27875532 PMC5119724

[11] Lewis C, Hutchinson JC, Riddington M, Hill M, Arthurs OJ, Fisher J, et al. Evidence synthesis: a systematic review of factors affecting uptake of autopsy examination. Minimally invasive autopsy for fetuses and children based on a combination of post-mortem MRI and endoscopic examination: a feasibility study. 2019. 10.3310/hta23460 PMC6732714 31461397

[12] Taylor AW, Blau DM, Bassat Q, Onyango D, Kotloff KL, Arifeen SE, et al. Initial findings from a novel population-based child mortality surveillance approach: a descriptive study. Lancet Glob Health. 2020;8(7):e909–e19. Epub 2020/06/21. doi: 10.1016/S2214-109X(20)30205-9 ; PubMed Central PMCID: PMC7303945. 32562647 PMC7303945

[13] Madhi SA, Pathirana J, Baillie V, Izu A, Bassat Q, Blau DM, et al. Unraveling specific causes of neonatal mortality using minimally invasive tissue sampling: an observational study. Clinical Infectious Diseases. 2019;69(Supplement_4):S351–S60. doi: 10.1093/cid/ciz574 31598660 PMC6785687

[14] Rakislova N, Fernandes F, Lovane L, Jamisse L, Castillo P, Sanz A, et al. Standardization of Minimally Invasive Tissue Sampling Specimen Collection and Pathology Training for the Child Health and Mortality Prevention Surveillance Network. Clinical Infectious Diseases. 2019;69(Supplement_4):S302–S10. doi: 10.1093/cid/ciz565 31598667 PMC6785668

[15] Salzberg NT, Sivalogan K, Bassat Q, Taylor AW, Adedini S, El Arifeen S, et al. Mortality Surveillance Methods to Identify and Characterize Deaths in Child Health and Mortality Prevention Surveillance Network Sites. Clin Infect Dis. 2019;69(Suppl 4):S262–s73. Epub 2019/10/11. doi: 10.1093/cid/ciz599 ; PubMed Central PMCID: PMC6785672. 31598664 PMC6785672

[16] Cunningham SA, Shaikh NI, Nhacolo A, Raghunathan PL, Kotloff K, Naser AM, et al. Health and demographic surveillance systems within the child health and mortality prevention surveillance network. Clinical Infectious Diseases. 2019;69(Supplement_4):S274–S9. doi: 10.1093/cid/ciz609 31598663 PMC6785673

[17] Diaz MH, Waller JL, Theodore MJ, Patel N, Wolff BJ, Benitez AJ, et al. Development and Implementation of Multiplex TaqMan Array Cards for Specimen Testing at Child Health and Mortality Prevention Surveillance Site Laboratories Clin Infect Dis. 2019;69:S311–S21. doi: 10.1093/cid/ciz571 31598666 PMC7108207

[18] Martines RB, Ritter JM, Gary J, et al. Pathology and telepathology methods in the Child Health and Mortality Prevention Surveillance Network. Clin Infect Dis. 2019;69:S322–S32. doi: 10.1093/cid/ciz579 31598668

[19] Blau DM, Caneer JP, Philipsborn RP, Madhi SA, Bassat Q, Varo R, et al. Overview and Development of the Child Health and Mortality Prevention Surveillance Determination of Cause of Death (DeCoDe) Process and DeCoDe Diagnosis Standards. Clin Infect Dis. 2019;69(Suppl 4):S333–s41. Epub 2019/10/11. doi: 10.1093/cid/ciz572 ; PubMed Central PMCID: PMC6785670. 31598661 PMC6785670

[20] World Health Organization. ICD-10: international statistical classification of diseases and related health problems, tenth revision, 2nd edn. Geneva: World Health Organization; 2004.

[21] World Health Organization. The WHO application of ICD-10 to deaths during the perinatal period: ICD-PM. Geneva: World Health Organization; 2016. p. https://apps.who.int/iris/bitstream/handle/10665/249515/9789241549752-eng.pdf.

[22] Madewell ZJ, Whitney CG, Velaphi S, Mutevedzi P, Mahtab S, Madhi SA, et al. Prioritizing Health Care Strategies to Reduce Childhood Mortality. JAMA Network Open. 2022;5(10):e2237689–e. doi: 10.1001/jamanetworkopen.2022.37689 36269354 PMC9587481

[23] Evaluation IIfHMa. Neonatal disorders—Level 3 cause [cited 2021 8 Dec]. Available from: https://www.healthdata.org/results/gbd_summaries/2019/neonatal-disorders-level-3-cause.

[24] Goldenberg RL, Dhaded S, Saleem S, Goudar SS, Tikmani SS, Trotta M, et al. Birth asphyxia is under‐rated as a cause of preterm neonatal mortality in low‐and middle‐income countries: A prospective, observational study from PURPOSe. BJOG: An International Journal of Obstetrics & Gynaecology. 2022. doi: 10.1111/1471-0528.17220 35593030

[25] Al-Sheyab NA, Khader YS, Shattnawi KK, Alyahya MS, Batieha A. Rate, risk factors, and causes of neonatal deaths in Jordan: analysis of data from Jordan stillbirth and neonatal surveillance system (JSANDS). Frontiers in Public Health. 2020;8. doi: 10.3389/fpubh.2020.595379 33194998 PMC7661434

[26] Ahmed I, Ali SM, Amenga-Etego S, Ariff S, Bahl R, Baqui AH, et al. Population-based rates, timing, and causes of maternal deaths, stillbirths, and neonatal deaths in south Asia and sub-Saharan Africa: a multi-country prospective cohort study. The Lancet Global Health. 2018;6(12):e1297–e308. doi: 10.1016/S2214-109X(18)30385-1 30361107 PMC6227247

[27] St Children. Surviving the First Day. London: Save the Children International; 2013.

[28] Chandraharan E, Arulkumaran S. Prevention of birth asphyxia: responding appropriately to cardiotocograph (CTG) traces. Best Pract Res Clin Obstet Gynaecol. 2007;21(4):609–24. Epub 2007/04/03. doi: 10.1016/j.bpobgyn.2007.02.008 . 17400026

[29] Buchmann EJ, Pattinson RC, Nyathikazi N. Intrapartum-related birth asphyxia in South Africa—lessons from the first national perinatal care survey. S Afr Med J. 2002;92(11):897–901. Epub 2003/01/01. . 12506592

[30] Thompson L, Crimmins S, Telugu B, Turan S. Intrauterine hypoxia: clinical consequences and therapeutic perspectives. Research and Reports in Neonatology. 2015;2015:79. doi: 10.2147/RRN.S57990

[31] Koshida S, Yanagi T, Ono T, Tsuji S, Takahashi K. Possible Prevention of Neonatal Death: A Regional Population-Based Study in Japan. Yonsei Med J. 2016;57(2):426–9. Epub 2016/02/06. doi: 10.3349/ymj.2016.57.2.426 ; PubMed Central PMCID: PMC4740536. 26847296 PMC4740536

[32] Lee AC, Kozuki N, Blencowe H, Vos T, Bahalim A, Darmstadt GL, et al. Intrapartum-related neonatal encephalopathy incidence and impairment at regional and global levels for 2010 with trends from 1990. Pediatr Res. 2013;74 Suppl 1(Suppl 1):50–72. Epub 2013/12/25. doi: 10.1038/pr.2013.206 ; PubMed Central PMCID: PMC3873711. 24366463 PMC3873711

[33] Dramowski A, Velaphi S, Reubenson G, Bekker A, Perovic O, Finlayson H, et al. National Neonatal Sepsis Task Force launch: Supporting infection prevention and surveillance, outbreak investigation and antimicrobial stewardship in neonatal units in South Africa. SAMJ: South African Medical Journal. 2020;110(5):360–3. 32657717 10.7196/SAMJ.2020.v110i5.14564

[34] Saha SK, Schrag SJ, El Arifeen S, Mullany LC, Islam MS, Shang N, et al. Causes and incidence of community-acquired serious infections among young children in south Asia (ANISA): an observational cohort study. The Lancet. 2018;392(10142):145–59. doi: 10.1016/S0140-6736(18)31127-9 30025808 PMC6053599

[35] Rhoda N, Velaphi S, Gebhardt G, Kauchali S, Barron P. Reducing neonatal deaths in South Africa: Progress and challenges. South African Medical Journal. 2018;108(3):9–16.

[36] Organization WH. WHO recommendations on health promotion interventions for maternal and newborn health Geneva: WHO; [cited 2021 12 Dec]. Available from: http://apps.who.int/iris/bitstream/handle/10665/172427/9789241508742_report_eng.pdf;jsessionid=B98F09AC9B4E12778CB2EF2605D390B9?sequence=1. 26180864

[37] Organization WH. Committing to implementation of the Global Strategy for Women’s, Children’s and Adolescents’ Health (2016–2030) Geneva: WHO; 2021 [cited 2021 8 Dec]. Available from: https://apps.who.int/gb/ebwha/pdf_files/WHA74/A74_14-en.pdf.

[38] Organization WH, UNICEF. Ending Preventable Newborn Deaths and Stillbirths by 2030. WHO, UNICEF; 2020.

