# Supplementary material for: Causes of death identified in neonates enrolled through Child Health and Mortality Prevention Surveillance (CHAMPS), December 2016 –December 2021
Source: PLOS Glob Public Health. 2023 Mar 20;3(3):e0001612. doi: 10.1371/journal.pgph.0001612 (PMC10027211; doi:10.1371/journal.pgph.0001612)
Supplement: S1 Text — (DOCX) [file pgph.0001612.s001.docx]

**Supplemental Text 1: Membership of CHAMPS Consortium**

For CHAMPS Bangladesh - A.S.M. Nawshad Uddin Ahmed and Mahbubul Hoque of Bangladesh Institute of Child Health at Dhaka University and Dhaka Shishu Children’s Hospital; Mohammed Kamal, Mohammed Mosiur, and Ferdousi Begum of Bangabandhu Sheikh Mujib Medical University (BSMMU); Saria Tasnim of Dhaka Community Medical College and Hospital; Meerjady Sabrina Flora of the Directorate General of Health Services in Bangladesh; Farida Ariuman of Institute of Cancer Research and Hospital, Iqbal Ansary Khan, Tahmina Shirin, and Mahbubur Rahman of Institute of Epidemiology, Disease Control and Research (IEDCR); Sanwarul Bari, Shahana Parveen, Farzana Islam, Mohammed Zahid Hossain, Kazi Munisul Islam, Mohammed Sabbir Ahmed, K. Zaman, Mustafizur Rahman, Dilruba Ahmed of International Centre for Diarrhoeal Disease Research, Bangladesh (icddr,b); Kyu Han Lee of the Department of Epidemiology, Johns Hopkins Bloomberg School of Public Health; and Ferdousi Islam of Popular Medical College and Hospital in Dhaka, Bangladesh

For CHAMPS Ethiopia – Joseph O. Oundo and Stian Orlien of London School of Hygiene & Tropical Medicine; Fikremelekot Temesgen of Addis Ababa University; Melisachew Mulatu Yeshi of Ayder Specialized Comprehensive Hospital at Mekelle University; Alexander M. Ibrahim, Tadesse Gure, and Addisu Alemu of the College of Health and Medical Sciences at Haramaya University; Yunus Edrid and Solomon Ali of the National Data Management Centre at the Ethiopian Public Health Institute; and Mahlet Abayneh Gizaw of St. Paul’s Hospital Millennium Medical College in Addis Ababa, Ethiopia

For CHAMPS Kenya -- Emily Rogena of Jomo Kenyatta University of Agriculture and Technology (JKUAT); Florence Murila of University of Nairobi; Gunturu Revathi of University of Aga Khan Medical College; Paul K. Mitei and Magdalene Kuria of Kisumu County Department of Health; and Jennifer R. Verani of the National Center for Immunization and Respiratory Diseases Branch at the Centers for Disease Control and Prevention

For CHAMPS Mali – Karen D. Fairchild of University of Virginia; Carol L. Greene, Rima Koka, Sharon M. Tennant, Ashka Mehta and J. Kristie Johnson of University of Maryland School of Medicine; Tatiana Keita of Clinique Pasteur in Bamako Mali; Adama Mamby Keita, Nana Kourouma, Uma U. Onwuchekwu, Doh Sanogo, Diakaridia Sidibe and Seydou Sissoko of Centre pour le Développement des Vaccins (CVD-Mali); and Diakaridia Kone of CSRef Commune I in Bamako, Mali

For CHAMPS Mozambique – Milton Kindcardett, Khátia Munguambe, Ariel Nhacolo, Tacilta Nhampossa, Pio Vitorino, Elisio Xerinda, and Justina Bramugy of Centro de Investigação em Saúde de Manhiça (CISM) in Maputo; Celso Monjane and Sheila Nhachungue of Instituto Nacional de Saúde (INS) in Maputo; Juan Carlos Hurtado, Maria Maixenchs, Clara Menéndez, Jaume Ordi, Natalia Rakislova, and Marta Valente of ISGlobal Hospital Clinic at Universitat de Barcelona; Dercio Chitungo and Zara Manhique of Quelimane Central Hospital; Sibone Mocumbi, Fabiola Fernandes, and Carla Carrilho of Eduarado Mondlane University and Maputo Central Hospital

From the CHAMPS Program Office – Rebecca Pass Philipsborn of Emory University and Children’s Healthcare of Atlanta; Jeffrey P. Koplan, Mischka Garel, and Betsy Dewey of Emory Global Health Institute; Shailesh Nair, Navit T. Salzberg, and Lucy Liu of the Public Health Informatics Institute at the Task Force for Global Health in Atlanta, Georgia, USA

From the CDC Central Pathology Lab – Rebecca Alkis-Ramirez of Center for Global Health at the US Centers for Disease Control and Prevention; Jana M. Ritter, Sherif R. Zaki, and Joy Gary of Infectious Diseases Pathology Branch, National Center for Emerging and Zoonotic Diseases at the US Centers for Disease Control and Prevention

For the CDC TaqMan Array (TAC) Team – Jonas M. Winchell, Jacob Witherbee, and Jessica L. Waller of the National Center for Immunization and Respiratory Diseases at the Centers for US Disease Control and Prevention

For CHAMPS Sierra Leone – Ruby Fayorsey of ICAP at Columbia University and Harlem Hospital Centers; Sandra Lako of Aberdeen Women’s Centre in Freetown; Erick Kaluma of Crown Agents; Foday Sesay of Sierra Leone’s Ministry of Health & Sanitation; Baindu Kosia and Samuel Pratt of FOCUS 1000

For CHAMPS South Africa – Portia Mutevedzi, Fatima Solomon, Ashleigh Fritz, Noluthando Dludlu, Constance Ntuli, and Richard Chawana of South African Council Vaccines and Infectious Diseases Analytics Research Unit, University of Witwatersrand; Karen Petersen, Sanjay G. Lala, Sithembiso Velaphi, and Yasmin Adam of Chris Hani Baragwanath Academic Hospital and University of Witwatersrand; Jeannette Wadula, Martin Hale and Peter J. Swart of National Health for Laboratory Service in South Africa; Hennie Lombaard of Rahima Moosa Mother and Child Hospital; and Gillian Sorour of Wits Health Consortium
