# Supplementary material for: Causes of death identified in neonates enrolled through Child Health and Mortality Prevention Surveillance (CHAMPS), December 2016 –December 2021
Source: PLOS Glob Public Health. 2023 Mar 20;3(3):e0001612. doi: 10.1371/journal.pgph.0001612 (PMC10027211; doi:10.1371/journal.pgph.0001612)
Supplement: S1 Table — (DOCX) [file pgph.0001612.s002.docx]

| **Supplemental Table 1. Characteristics of CHAMPS neonatal cases for whom MITS was performed versus all eligible neonatal deaths never enrolled in CHAMPS or consented to MITS (2017-2020).** | | | |
| --- | --- | --- | --- |
|  | **Total** | | |
|  | MITS | Non-MITS | P-value |
|  |  | (+ DSS only) |  |
|  | n = 631 | n = 699 |  |
|  | n (%)^1^ | n (%)^1^ |  |
| **Case characteristics** |  |  |  |
| **Sex^1^** |  |  | 0.156 |
| Female | 267 (42) | 285 (41) |  |
| Male | 362 (58) | 411 (59) |  |
| **Location of death^1^** |  |  | <0.001 |
| Community | 44 (7) | 229 (33) |  |
| Facility | 587 (93) | 466 (67) |  |
| **Age group** |  |  | 0.248 |
| Death in first 24 hours | 284 (45) | 284 (40) |  |
| Early neonate (1 to 6 days) | 226 (36) | 264 (38) |  |
| Late neonate (7 to 27 days) | 121 (19) | 151 (22) |  |
| CHAMPS, Child Health and Mortality Prevention Surveillance Network; DSS, Demographic Surveillance System; MITS, minimally invasive tissue sampling; VA, verbal autopsy. | | | |
| ^1^Missing: sex (Kenya=4, South Africa=1); location of death (Bangladesh=4). | |  |  |
