# Supplementary material for: Causes of death identified in neonates enrolled through Child Health and Mortality Prevention Surveillance (CHAMPS), December 2016 –December 2021
Source: PLOS Glob Public Health. 2023 Mar 20;3(3):e0001612. doi: 10.1371/journal.pgph.0001612 (PMC10027211; doi:10.1371/journal.pgph.0001612)
Supplement: S2 Table — (DOCX) [file pgph.0001612.s003.docx]

| **Supplemental Table 2: Characteristics of deaths enrolled in CHAMPS that occurred during the neonatal period, by age at death (2016-2021)** | | | | | | | | |
| --- | --- | --- | --- | --- | --- | --- | --- | --- |
| Characteristics | All | Bangladesh | Ethiopia | Kenya | Mali | Mozambique | Sierra Leone | South Africa |
|  | (N=1458) | (N=180) | (N=110) | (N=181) | (N=74) | (N=306) | (N=144) | (N=463) |
|  | n (%) | n (%) | n (%) | n (%) | n (%) | n (%) | n (%) | n (%) |
| Median (range) age at death, in days | 2 (0, 5) | 1 (0, 2) | 1 (0, 4) | 1 (0, 2) | 2 (0, 6) | 1 (0, 4) | 2 (1, 5) | 3 (1, 7) |
| Gender, n (%) |  |  |  |  |  |  |  |  |
| Female | 620 (42.5) | 106 (58.9) | 66 (60.0) | 98 (54.1) | 40 (54.1) | 180 (58.8) | 79 (54.9) | 266 (57.5) |
| Male | 835 (57.3) | 74 (41.1) | 44 (40.0) | 82 (45.3) | 34 (45.9) | 126 (41.2) | 65 (45.1) | 195 (42.1) |
| Indeterminate | 3 (0.2) | 0 (0) | 0 (0) | 1 (0.6) | 0 (0) | 0 (0) | 0 (0) | 2 (0.4) |
| HIV Status, n (%) |  |  |  |  |  |  |  |  |
| HIV infected | 7 (0.5) | 1 (0.6) | 0 (0) | 2 (1.1) | 0 (0) | 2 (0.7) | 1 (0.7) | 1 (0.2) |
| HIV exposed uninfected | 285 (19.5) | 0 (0) | 4 (3.6) | 28 (15.5) | 0 (0) | 81 (26.5) | 4 (2.8) | 168 (36.3) |
| HIV uninfected | 1166 (80.0) | 179 (99.4) | 106 (96.4) | 151 (83.4) | 74 (100.0) | 223 (72.9) | 139 (96.5) | 294 (63.5) |
| Mode of delivery, n (%) |  |  |  |  |  |  |  |  |
| Normal vaginal delivery | 875 (60.0) | 95 (52.8) | 55 (50.0) | 143 (79.0) | 64 (86.5) | 199 (65.0) | 92 (63.9) | 227 (49.0) |
| Cesarean section | 397 (27.2) | 79 (43.9) | 26 (23.6) | 34 (18.8) | 8 (10.8) | 47 (15.4) | 39 (27.1) | 164 (35.4) |
| Instrumentation (e.g., forceps) | 1 (0.1) | 0 (0) | 1 (0.9) | 0 (0) | 0 (0) | 0 (0) | 0 (0) | 0 (0) |
| Unknown | 185 (12.7) | 6 (3.3) | 28 (25.5) | 4 (2.2) | 2 (2.7) | 60 (19.6) | 13 (9.0) | 72 (15.6) |
| Gestational age, n (%) |  |  |  |  |  |  |  |  |
| ≤28 weeks | 278 (19.1) | 23 (12.8) | 5 (4.5) | 21 (11.6) | 4 (5.4) | 26 (8.5) | 6 (4.2) | 193 (41.7) |
| 28-33 weeks | 225 (15.4) | 32 (17.8) | 15 (13.6) | 28 (15.5) | 11 (14.9) | 38 (12.4) | 10 (6.9) | 91 (19.7) |
| 34-36 weeks | 130 (8.9) | 20 (11.1) | 13 (11.8) | 13 (7.2) | 5 (6.8) | 27 (8.8) | 18 (12.5) | 34 (7.3) |
| 37-42 weeks | 348 (23.9) | 44 (24.4) | 12 (10.9) | 72 (39.8) | 26 (35.1) | 81 (26.5) | 47 (32.6) | 66 (14.3) |
| unknown | 477 (32.7) | 61 (33.9) | 65 (59.1) | 47 (26.0) | 28 (37.8) | 134 (43.8) | 63 (43.8) | 79 (17.1) |
| Birth weight, n (%) |  |  |  |  |  |  |  |  |
| Extremely low birth weight (<1000 gm) | 249 (17.1) | 31 (17.2) | 2 (1.8) | 21 (11.6) | 1 (1.4) | 15 (4.9) | 4 (2.8) | 175 (37.8) |
| Very low birth weight (1000-1499 gm) | 248 (17.0) | 31 (17.2) | 20 (18.2) | 26 (14.4) | 15 (20.3) | 38 (12.4) | 17 (11.8) | 101 (21.8) |
| Low birth weight (1500-2499 gm) | 297 (20.4) | 54 (30.0) | 27 (24.5) | 37 (20.4) | 17 (23.0) | 67 (21.9) | 27 (18.8) | 68 (14.7) |
| Normal weight (2500- 4000 gm) | 459 (31.5) | 43 (23.9) | 38 (34.5) | 84 (46.4) | 37 (50.0) | 120 (39.2) | 74 (51.4) | 63 (13.6) |
| Macrosomia (>4000) | 12 (0.8) | 2 (1.1) | NA | 1 (0.6) | 1 (1.4) | 2 (0.7) | 3 (2.1) | 3 (0.6) |
| Missing | 193 (13.2) | 19 (10.6) | 23 (20.9) | 12 (6.6) | 3 (4.1) | 64 (20.9) | 19 (13.2) | 53 (11.4) |
| Median (range) weight at MITS**, in grams | 1870 (1100, 2800) | 1655 (1104, 2436) | 2035 (1462, 2838) | 2200 (1308, 3050) | 2330 (1478, 3100) | 2400 (1500, 3000) | 2650 (1798, 3125) | 1170 (725, 2145) |
| Median (range) of hours between death and MITS done | 12 (4, 21) | 1 (1, 2) | 3 (1, 8) | 16 (10, 21) | 7 (4, 13) | 10 (5, 17) | 6.5 (4, 13) | 26 (17, 35) |
| Location of birth, n (%) |  |  |  |  |  |  |  |  |
| Hospital | 862 (59.1) | 120 (66.7) | 52 (47.3) | 120 (66.3) | 3 (4.1) | 131 (42.8) | 80 (55.6) | 356 (76.9) |
| Health Centre | 165 (11.3) | 13 (7.2) | 3 (2.7) | 16 (8.8) | 58 (78.4) | 47 (15.4) | 4 (2.8) | 24 (5.2) |
| Health Post | 12 (0.8) | 0 (0) | 0 (0) | 4 (2.2) | 0 (0) | 2 (0.7) | 6 (4.2) | 0 (0) |
| Home | 54 (3.7) | 19 (10.6) | 1 (0.9) | 10 (5.5) | 2 (2.7) | 6 (2.0) | 3 (2.1) | 13 (2.8) |
| On the way to health center | 13 (0.9) | 4 (2.2) | 0 (0) | 1 (0.6) | 0 (0) | 3 (1.0) | 1 (0.7) | 4 (0.9) |
| Other location | 10 (0.7) | 0 (0) | 0 (0) | 0 (0) | 4 (5.4) | 1 (0.3) | 0 (0) | 5 (1.1) |
| Not recorded | 342 (23.5) | 24 (13.3) | 54 (49.1) | 30 (16.6) | 7 (9.5) | 116 (37.9) | 50 (34.7) | 61 (13.2) |
| Location of death, n (%) |  |  |  |  |  |  |  |  |
| Community | 77 (5.3) | 9 (5.0) | 6 (5.5) | 31 (17.1) | 14 (18.9) | 10 (3.3) | 3 (2.1) | 4 (0.9) |
| Health facility | 1381 (94.7) | 171 (95.0) | 104 (94.5) | 150 (82.9) | 60 (81.1) | 296 (96.7) | 141 (97.9) | 459 (99.1) |
| Median (range) of hours in hospital*** | 34 (11, 101) | 20 (6, 41) | 36 (12, 106) | 23 (7, 32) | 35 (7, 121) | 29 (10, 72) | 44 (18, 82) | 71 (20, 162) |
