# Supplementary material for: Causes of death identified in neonates enrolled through Child Health and Mortality Prevention Surveillance (CHAMPS), December 2016 –December 2021
Source: PLOS Glob Public Health. 2023 Mar 20;3(3):e0001612. doi: 10.1371/journal.pgph.0001612 (PMC10027211; doi:10.1371/journal.pgph.0001612)
Supplement: S3 Table — (DOCX) [file pgph.0001612.s004.docx]

|  | **Supplemental Table 3: WHO ICD10 Perinatal Mortality underlying cause of death categories & specific underlying cause of death by age group** | | | | | | |
| --- | --- | --- | --- | --- | --- | --- | --- |
|  |  |  | All | Death in first 24 hours | Early Neonate death | Late Neonate Death |  |
|  |  |  | (N=1458) | (N=596) | (N=593) | (N=269) |  |
|  | **Congenital malformation N1 (N=118)** |  |  |  |  |  |  |
|  | *Congenital malformation of nervous system* |  |  |  |  |  |  |
|  | Anencephaly | Q00.0 | 7 (0.5) | 6 (1.0) | 1 (0.2) | 0 (0) |  |
|  | Occipital encephalocele | Q01.2 | 1 (0.1) | 0 (0) | 1 (0.2) | 0 (0) |  |
|  | Atresia of foramina of Magendie and Luschka | Q03.1 | 1 (0.1) | 0 (0) | 1 (0.2) | 0 (0) |  |
|  | Congenital hydrocephalus, unspecified | Q03.9 | 1 (0.1) | 1 (0.2) | 0 (0) | 0 (0) |  |
|  | Holoprosencephaly | Q04.2 | 2 (0.1) | 1 (0.2) | 1 (0.2) | 0 (0) |  |
|  | Other reduction deformities of brain | Q04.3 | 1 (0.1) | 1 (0.2) | 0 (0) | 0 (0) |  |
|  | Congenital malformation of brain, unspecified | Q04.9 | 1 (0.1) | 0 (0) | 1 (0.2) | 0 (0) |  |
|  | Spina bifida | Q05 | 1 (0.1) | 1 (0.2) | 0 (0) | 0 (0) |  |
|  | Thoracic spina bifida with hydrocephalus | Q05.1 | 1 (0.1) | 1 (0.2) | 0 (0) | 0 (0) |  |
|  | Lumbar spina bifida with hydrocephalus | Q05.2 | 1 (0.1) | 1 (0.2) | 0 (0) | 0 (0) |  |
|  | Lumbar spina bifida without hydrocephalus | Q05.7 | 2 (0.1) | 1 (0.2) | 0 (0) | 1 (0.4) |  |
|  | Spina bifida, unspecified | Q05.9 | 2 (0.1) | 0 (0) | 1 (0.2) | 1 (0.4) |  |
|  | *Congenital malformations of circulatory system* |  |  |  |  |  |  |
|  | Hypoplastic left heart syndrome | Q23.4 | 5 (0.3) | 0 (0) | 3 (0.5) | 2 (0.7) |  |
|  | Other specified congenital malformations of heart | Q24.8 | 1 (0.1) | 1 (0.2) | 0 (0) | 0 (0) |  |
|  | Congenital malformation of heart, unspecified | Q24.9 | 5 (0.3) | 1 (0.2) | 2 (0.3) | 2 (0.7) |  |
|  | *Congenital malformation of digestive system* |  |  |  |  |  |  |
|  | Congenital malformations of lips, not elsewhere classified | Q38.0 | 1 (0.1) | 0 (0) | 0 (0) | 1 (0.4) |  |
|  | Congenital tracheo-oesophageal fistula without atresia | Q39.2 | 1 (0.1) | 0 (0) | 0 (0) | 1 (0.4) |  |
|  | Congenital absence, atresia and stenosis of jejunum | Q41.1 | 1 (0.1) | 0 (0) | 0 (0) | 1 (0.4) |  |
|  | Congenital absence, atresia and stenosis of anus with fistula | Q42.2 | 1 (0.1) | 0 (0) | 1 (0.2) | 0 (0) |  |
|  | Congenital absence, atresia and stenosis of anus without fistula | Q42.3 | 1 (0.1) | 0 (0) | 1 (0.2) | 0 (0) |  |
|  | *Congenital malformation of urinary system* |  |  |  |  |  |  |
|  | Polycystic kidney, unspecified | Q61.3 | 1 (0.1) | 0 (0) | 1 (0.2) | 0 (0) |  |
|  | Cystic kidney disease, unspecified | Q61.9 | 1 (0.1) | 1 (0.2) | 0 (0) | 0 (0) |  |
|  | Other congenital malformations of urinary system | Q64 | 1 (0.1) | 1 (0.2) | 0 (0) | 0 (0) |  |
|  | Congenital posterior urethral valves | Q64.2 | 2 (0.1) | 1 (0.2) | 0 (0) | 1 (0.4) |  |
|  | Congenital malformation of urinary system, unspecified | Q64.9 | 2 (0.1) | 1 (0.2) | 0 (0) | 1 (0.4) |  |
|  | *Congenital malformations & deformations of musculoskeletal system* |  |  |  |  |  |  |
|  | Congenital absence of unspecified limb(s) | Q73.0 | 1 (0.1) | 1 (0.2) | 0 (0) | 0 (0) |  |
|  | Macrocephaly | Q75.3 | 2 (0.1) | 2 (0.3) | 0 (0) | 0 (0) |  |
|  | Osteochondrodysplasia with defects of growth of tubular bones and spine | Q77 | 1 (0.1) | 0 (0) | 1 (0.2) | 0 (0) |  |
|  | Thanatophoric short stature | Q77.1 | 2 (0.1) | 2 (0.3) | 0 (0) | 0 (0) |  |
|  | Osteogenesis imperfecta | Q78.0 | 1 (0.1) | 0 (0) | 1 (0.2) | 0 (0) |  |
|  | Osteochondrodysplasia, unspecified | Q78.9 | 1 (0.1) | 0 (0) | 1 (0.2) | 0 (0) |  |
|  | Congenital malformations of the musculoskeletal system, not elsewhere classified | Q79 | 1 (0.1) | 1 (0.2) | 0 (0) | 0 (0) |  |
|  | Congenital diaphragmatic hernia | Q79.0 | 4 (0.3) | 2 (0.3) | 1 (0.2) | 1 (0.4) |  |
|  | Exomphalos | Q79.2 | 2 (0.1) | 0 (0) | 1 (0.2) | 1 (0.4) |  |
|  | Gastroschisis | Q79.3 | 14 (1.0) | 1 (0.2) | 8 (1.3) | 5 (1.9) |  |
|  | *Other congenital malformations* |  |  |  |  |  |  |
|  | Other congenital malformations of face and neck | Q18 | 1 (0.1) | 1 (0.2) | 0 (0) | 0 (0) |  |
|  | Congenital cystic lung | Q33.0 | 1 (0.1) | 1 (0.2) | 0 (0) | 0 (0) |  |
|  | Epidermolysis bullosa, unspecified | Q81.9 | 2 (0.1) | 0 (0) | 0 (0) | 2 (0.7) |  |
|  | Other specified congenital malformation syndromes affecting multiple systems | Q87 | 4 (0.3) | 2 (0.3) | 2 (0.3) | 0 (0) |  |
|  | Congenital malformation syndromes predominantly involving limbs | Q87.2 | 3 (0.2) | 1 (0.2) | 1 (0.2) | 1 (0.4) |  |
|  | Other specified congenital malformation syndromes, not elsewhere classified | Q87.8 | 1 (0.1) | 0 (0) | 1 (0.2) | 0 (0) |  |
|  | Other congenital malformations, not elsewhere classified | Q89 | 1 (0.1) | 0 (0) | 1 (0.2) | 0 (0) |  |
|  | Multiple congenital malformations, not elsewhere classified | Q89.7 | 1 (0.1) | 1 (0.2) | 0 (0) | 0 (0) |  |
|  | Congenital malformation, unspecified | Q89.9 | 9 (0.6) | 5 (0.8) | 2 (0.3) | 2 (0.7) |  |
|  | Neoplasm of uncertain or unknown behaviour: Bone and articular cartilage | D48.0 | 1 (0.1) | 1 (0.2) | 0 (0) | 0 (0) |  |
|  | *Chromosomal abnormalities, not elsewhere classified* |  |  |  |  |  |  |
|  | Down syndrome | Q90 | 1 (0.1) | 1 (0.2) | 0 (0) | 0 (0) |  |
|  | Down syndrome, unspecified | Q90.9 | 2 (0.1) | 0 (0) | 1 (0.2) | 1 (0.4) |  |
|  | Edwards syndrome, unspecified | Q91.3 | 4 (0.3) | 1 (0.2) | 1 (0.2) | 2 (0.7) |  |
|  | Patau syndrome, unspecified | Q91.7 | 5 (0.3) | 1 (0.2) | 3 (0.5) | 1 (0.4) |  |
|  | Chromosome replaced with ring or dicentric | Q93.2 | 1 (0.1) | 0 (0) | 1 (0.2) | 0 (0) |  |
|  | Chromosomal abnormality, unspecified | Q99.9 | 7 (0.5) | 3 (0.5) | 1 (0.2) | 3 (1.1) |  |
|  | *Congenital myopathies* | G71.2 | 1 (0.1) | 0 (0) | 0 (0) | 1 (0.4) |  |
|  | **Disorders related to fetal growth N2 (N=11)** |  |  |  |  |  |  |
|  | Light for gestational age | P05.0 | 6 (0.4) | 3 (0.5) | 2 (0.3) | 1 (0.4) |  |
|  | Small for gestational age | P05.1 | 1 (0.1) | 0 (0) | 0 (0) | 1 (0.4) |  |
|  | Slow fetal growth, unspecified | P05.9 | 4 (0.3) | 1 (0.2) | 2 (0.3) | 1 (0.4) |  |
|  | **Birth trauma N3 (N=1)** |  |  |  |  |  |  |
|  | Other birth injuries | P15 | 1 (0.1) | 0 (0) | 1 (0.2) | 0 (0) |  |
|  | **Complications of intrapartum events N4 (N=446)** |  |  |  |  |  |  |
|  | *Intrauterine hypoxia* |  |  |  |  |  |  |
|  | Intrauterine hypoxia | P20 | 6 (0.4) | 4 (0.7) | 2 (0.3) | 0 (0) |  |
|  | Intrauterine hypoxia first noted before onset of labour | P20.0 | 7 (0.5) | 7 (1.2) | 0 (0) | 0 (0) |  |
|  | Intrauterine hypoxia first noted during labour and delivery | P20.1 | 109 (7.5) | 71 (11.9) | 32 (5.4) | 6 (2.2) |  |
|  | Intrauterine hypoxia, unspecified | P20.9 | 111 (7.6) | 61 (10.2) | 47 (7.9) | 3 (1.1) |  |
|  | *Birth asphyxia* |  |  |  |  |  |  |
|  | Birth asphyxia | P21 | 5 (0.3) | 2 (0.3) | 2 (0.3) | 1 (0.4) |  |
|  | Severe birth asphyxia | P21.0 | 131 (9.0) | 70 (11.7) | 56 (9.4) | 5 (1.9) |  |
|  | Mild and moderate birth asphyxia | P21.1 | 37 (2.5) | 17 (2.9) | 20 (3.4) | 0 (0) |  |
|  | Birth asphyxia, unspecified | P21.9 | 34 (2.3) | 15 (2.5) | 16 (2.7) | 3 (1.1) |  |
|  | *Complications of placenta, cord and membranes* |  |  |  |  |  |  |
|  | Fetus and newborn affected by placental transfusion syndromes | P02.3 | 1 (0.1) | 1 (0.2) | 0 (0) | 0 (0) |  |
|  | Fetus and newborn affected by other compression of umbilical cord | P02.5 | 1 (0.1) | 1 (0.2) | 0 (0) | 0 (0) |  |
|  | Fetus and newborn affected by chorioamnionitis | P02.7 | 3 (0.2) | 3 (0.5) | 0 (0) | 0 (0) |  |
|  | HELLP syndrome | O14.2 | 1 (0.1) | 0 (0) | 1 (0.2) | 0 (0) |  |
|  | **Convulsion and disorder of cerebral status N5 (N=16)** |  |  |  |  |  |  |
|  | Other disturbances of cerebral status of newborn | P91 | 2 (0.1) | 2 (0.3) | 0 (0) | 0 (0) |  |
|  | Neonatal cerebral ischaemia | P91.0 | 2 (0.1) | 0 (0) | 2 (0.3) | 0 (0) |  |
|  | Hypoxic ischaemic encephalopathy of newborn | P91.6 | 10 (0.7) | 3 (0.5) | 5 (0.8) | 2 (0.7) |  |
|  | Other specified disturbances of cerebral status of newborn | P91.8 | 2 (0.1) | 1 (0.2) | 1 (0.2) | 0 (0) |  |
|  | **Infections N6 (N=254)** |  |  |  |  |  |  |
|  | *Other gastroenteritis and colitis of infectious and unspecified origin* |  |  |  |  |  |  |
|  | Other gastroenteritis and colitis of infectious and unspecified origin | A09 | 1 (0.1) | 0 (0) | 1 (0.2) | 0 (0) |  |
|  | Gastroenteritis and colitis of unspecified origin | A09.9 | 1 (0.1) | 0 (0) | 0 (0) | 1 (0.4) |  |
|  | *Other bacterial diseases* |  |  |  |  |  |  |
|  | Sepsis due to streptococcus, group B | A40.1 | 1 (0.1) | 1 (0.2) | 0 (0) | 0 (0) |  |
|  | Sepsis due to Streptococcus pneumoniae | A40.3 | 1 (0.1) | 0 (0) | 0 (0) | 1 (0.4) |  |
|  | Sepsis due to other Gram-negative organisms | A41.5 | 2 (0.1) | 1 (0.2) | 1 (0.2) | 0 (0) |  |
|  | Other specified sepsis | A41.8 | 2 (0.1) | 0 (0) | 0 (0) | 2 (0.7) |  |
|  | *Congenital syphilis* |  |  |  |  |  |  |
|  | Congenital syphilis | A50 | 4 (0.3) | 3 (0.5) | 1 (0.2) | 0 (0) |  |
|  | Early congenital syphilis, unspecified | A50.2 | 1 (0.1) | 0 (0) | 1 (0.2) | 0 (0) |  |
|  | Congenital syphilis, unspecified | A50.9 | 2 (0.1) | 0 (0) | 2 (0.3) | 0 (0) |  |
|  | *Viral infection of central nervous system* |  |  |  |  |  |  |
|  | Unspecified viral encephalitis | A86 | 1 (0.1) | 0 (0) | 0 (0) | 1 (0.4) |  |
|  | *Viral infections characterized by skin and mucous membrane lesions* |  |  |  |  |  |  |
|  | Measles | B05 | 1 (0.1) | 0 (0) | 0 (0) | 1 (0.4) |  |
|  | *Human immunodeficiency virus* |  |  |  |  |  |  |
|  | Human immunodeficiency virus [HIV] disease resulting in other conditions | B23 | 1 (0.1) | 0 (0) | 1 (0.2) | 0 (0) |  |
|  | *Other viral diseases* |  |  |  |  |  |  |
|  | Other specified viral diseases | B33.8 | 1 (0.1) | 0 (0) | 0 (0) | 1 (0.4) |  |
|  | *Bacterial meningitis* |  |  |  |  |  |  |
|  | Streptococcal meningitis | G00.2 | 1 (0.1) | 1 (0.2) | 0 (0) | 0 (0) |  |
|  | Other bacterial meningitis | G00.8 | 2 (0.1) | 0 (0) | 0 (0) | 2 (0.7) |  |
|  | Bacterial meningitis, unspecified | G00.9 | 1 (0.1) | 0 (0) | 0 (0) | 1 (0.4) |  |
|  | Meningitis, unspecified | G03.9 | 1 (0.1) | 0 (0) | 1 (0.2) | 0 (0) |  |
|  | *Bacterial & viral pneumonia* |  |  |  |  |  |  |
|  | Respiratory syncytial virus pneumonia | J12.1 | 1 (0.1) | 0 (0) | 0 (0) | 1 (0.4) |  |
|  | Pneumonia due to Klebsiella pneumoniae | J15.0 | 5 (0.3) | 0 (0) | 3 (0.5) | 2 (0.7) |  |
|  | Pneumonia due to Pseudomonas | J15.1 | 1 (0.1) | 0 (0) | 1 (0.2) | 0 (0) |  |
|  | Pneumonia due to streptococcus, group B | J15.3 | 1 (0.1) | 1 (0.2) | 0 (0) | 0 (0) |  |
|  | Pneumonia due to Escherichia coli | J15.5 | 1 (0.1) | 0 (0) | 1 (0.2) | 0 (0) |  |
|  | Other bacterial pneumonia | J15.8 | 7 (0.5) | 0 (0) | 3 (0.5) | 4 (1.5) |  |
|  | Bacterial pneumonia, unspecified | J15.9 | 1 (0.1) | 0 (0) | 0 (0) | 1 (0.4) |  |
|  | Pneumonia due to other specified infectious organisms | J16.8 | 3 (0.2) | 0 (0) | 0 (0) | 3 (1.1) |  |
|  | Pneumonia, organism unspecified | J18 | 2 (0.1) | 0 (0) | 0 (0) | 2 (0.7) |  |
|  | Bronchopneumonia, unspecified | J18.0 | 4 (0.3) | 2 (0.3) | 1 (0.2) | 1 (0.4) |  |
|  | Pneumonia, unspecified | J18.9 | 2 (0.1) | 0 (0) | 0 (0) | 2 (0.7) |  |
|  | *Congenital pneumonia* |  |  |  |  |  |  |
|  | Congenital pneumonia | P23 | 1 (0.1) | 1 (0.2) | 0 (0) | 0 (0) |  |
|  | Congenital pneumonia due to viral agent | P23.0 | 1 (0.1) | 1 (0.2) | 0 (0) | 0 (0) |  |
|  | Congenital pneumonia due to streptococcus, group B | P23.3 | 2 (0.1) | 2 (0.3) | 0 (0) | 0 (0) |  |
|  | Congenital pneumonia due to Escherichia coli | P23.4 | 3 (0.2) | 2 (0.3) | 1 (0.2) | 0 (0) |  |
|  | Congenital pneumonia due to other bacterial agents | P23.6 | 10 (0.7) | 4 (0.7) | 6 (1.0) | 0 (0) |  |
|  | Congenital pneumonia, unspecified | P23.9 | 16 (1.1) | 12 (2.0) | 4 (0.7) | 0 (0) |  |
|  | *Infection related to perinatal period* |  |  |  |  |  |  |
|  | Congenital cytomegalovirus infection | P35.1 | 7 (0.5) | 1 (0.2) | 5 (0.8) | 1 (0.4) |  |
|  | Congenital herpesviral [herpes simplex] infection | P35.2 | 1 (0.1) | 0 (0) | 0 (0) | 1 (0.4) |  |
|  | Other congenital viral diseases | P35.8 | 1 (0.1) | 0 (0) | 0 (0) | 1 (0.4) |  |
|  | Sepsis of newborn due to streptococcus, group B | P36.0 | 22 (1.5) | 15 (2.5) | 4 (0.7) | 3 (1.1) |  |
|  | Sepsis of newborn due to other and unspecified streptococci | P36.1 | 5 (0.3) | 1 (0.2) | 3 (0.5) | 1 (0.4) |  |
|  | Sepsis of newborn due to Staphylococcus aureus | P36.2 | 3 (0.2) | 0 (0) | 1 (0.2) | 2 (0.7) |  |
|  | Sepsis of newborn due to Escherichia coli | P36.4 | 16 (1.1) | 6 (1.0) | 5 (0.8) | 5 (1.9) |  |
|  | Sepsis of newborn due to anaerobes | P36.5 | 2 (0.1) | 0 (0) | 0 (0) | 2 (0.7) |  |
|  | Other bacterial sepsis of newborn | P36.8 | 75 (5.1) | 14 (2.3) | 37 (6.2) | 24 (8.9) |  |
|  | Bacterial sepsis of newborn, unspecified | P36.9 | 23 (1.6) | 3 (0.5) | 14 (2.4) | 6 (2.2) |  |
|  | Congenital toxoplasmosis | P37.1 | 2 (0.1) | 1 (0.2) | 0 (0) | 1 (0.4) |  |
|  | Neonatal (disseminated) listeriosis | P37.2 | 6 (0.4) | 1 (0.2) | 3 (0.5) | 2 (0.7) |  |
|  | Neonatal candidiasis | P37.5 | 1 (0.1) | 1 (0.2) | 0 (0) | 0 (0) |  |
|  | Other specified congenital infectious and parasitic diseases | P37.8 | 1 (0.1) | 0 (0) | 0 (0) | 1 (0.4) |  |
|  | Omphalitis of newborn with or without mild haemorrhage | P38 | 1 (0.1) | 0 (0) | 0 (0) | 1 (0.4) |  |
|  | *COVID-19* | U07.1 | 2 (0.1) | 0 (0) | 0 (0) | 2 (0.7) |  |
|  | *Other infectious or parasitic diseases* | P00.2 | 1 (0.1) | 1 (0.2) | 0 (0) | 0 (0) |  |
|  | **Respiratory and cardiovascular disorders N7 (N=159)** |  |  |  |  |  |  |
|  | *Respiratory distress syndrome of newborn* | P22.0 | 125 (8.5) | 62 (10.4) | 55 (9.3) | 8 (3.0) |  |
|  | *Neonatal aspiration syndromes* |  |  |  |  |  |  |
|  | Neonatal aspiration syndromes | P24 | 1 (0.1) | 1 (0.2) | 0 (0) | 0 (0) |  |
|  | Neonatal aspiration of meconium | P24.0 | 25 (1.7) | 14 (2.3) | 9 (1.5) | 2 (0.7) |  |
|  | Neonatal aspiration of amniotic fluid and mucus | P24.1 | 1 (0.1) | 1 (0.2) | 0 (0) | 0 (0) |  |
|  | Neonatal aspiration syndrome, unspecified | P24.9 | 3 (0.2) | 0 (0) | 1 (0.2) | 2 (0.7) |  |
|  | *Pulmonary haemorrhage originating in the perinatal period* |  |  |  |  |  |  |
|  | Pulmonary haemorrhage originating in the perinatal period | P26 | 1 (0.1) | 0 (0) | 1 (0.2) | 0 (0) |  |
|  | Unspecified pulmonary haemorrhage originating in the perinatal period | P26.9 | 2 (0.1) | 1 (0.2) | 0 (0) | 1 (0.4) |  |
|  | *Other respiratory conditions originating in the perinatal period* |  |  |  |  |  |  |
|  | Primary atelectasis of newborn | P28.0 | 1 (0.1) | 1 (0.2) | 0 (0) | 0 (0) |  |
|  | **Other neonatal conditions N8 (N=25)** |  |  |  |  |  |  |
|  | Nutritional marasmus | E41 | 2 (0.1) | 0 (0) | 0 (0) | 2 (0.7) |  |
|  | Hyperosmolality and hypernatraemia | E87.0 | 1 (0.1) | 0 (0) | 0 (0) | 1 (0.4) |  |
|  | Metabolic disorder, unspecified | E88.9 | 1 (0.1) | 0 (0) | 1 (0.2) | 0 (0) |  |
|  | Liver disease, unspecified | K76.9 | 1 (0.1) | 0 (0) | 0 (0) | 1 (0.4) |  |
|  | Fetus and newborn affected by caesarean delivery | P03.4 | 1 (0.1) | 0 (0) | 0 (0) | 1 (0.4) |  |
|  | Massive umbilical haemorrhage of newborn | P51.0 | 1 (0.1) | 1 (0.2) | 0 (0) | 0 (0) |  |
|  | Umbilical haemorrhage of newborn, unspecified | P51.9 | 1 (0.1) | 1 (0.2) | 0 (0) | 0 (0) |  |
|  | Intracerebral (nontraumatic) haemorrhage of fetus and newborn | P52.4 | 3 (0.2) | 3 (0.5) | 0 (0) | 0 (0) |  |
|  | Rh isoimmunization of fetus and newborn | P55.0 | 1 (0.1) | 0 (0) | 1 (0.2) | 0 (0) |  |
|  | ABO isoimmunization of fetus and newborn | P55.1 | 1 (0.1) | 0 (0) | 0 (0) | 1 (0.4) |  |
|  | Haemolytic disease of fetus and newborn, unspecified | P55.9 | 1 (0.1) | 0 (0) | 1 (0.2) | 0 (0) |  |
|  | Hydrops fetalis due to other and unspecified haemolytic disease | P56.9 | 1 (0.1) | 1 (0.2) | 0 (0) | 0 (0) |  |
|  | Kernicterus, unspecified | P57.9 | 1 (0.1) | 0 (0) | 1 (0.2) | 0 (0) |  |
|  | Neonatal jaundice associated with preterm delivery | P59.0 | 1 (0.1) | 0 (0) | 1 (0.2) | 0 (0) |  |
|  | Neonatal jaundice from other and unspecified hepatocellular damage | P59.2 | 1 (0.1) | 0 (0) | 0 (0) | 1 (0.4) |  |
|  | Syndrome of infant of a diabetic mother | P70.1 | 1 (0.1) | 0 (0) | 1 (0.2) | 0 (0) |  |
|  | Intestinal obstruction of newborn, unspecified | P76.9 | 1 (0.1) | 0 (0) | 1 (0.2) | 0 (0) |  |
|  | Necrotizing enterocolitis of fetus and newborn | P77 | 1 (0.1) | 0 (0) | 0 (0) | 1 (0.4) |  |
|  | Hydrops fetalis not due to haemolytic disease | P83.2 | 1 (0.1) | 1 (0.2) | 0 (0) | 0 (0) |  |
|  | Other and unspecified oedema specific to fetus and newborn | P83.3 | 1 (0.1) | 1 (0.2) | 0 (0) | 0 (0) |  |
|  | Acute abdomen | R10.0 | 1 (0.1) | 0 (0) | 0 (0) | 1 (0.4) |  |
|  | Other lack of expected normal physiological development | R62.8 | 1 (0.1) | 0 (0) | 0 (0) | 1 (0.4) |  |
|  | **Low birth weight/prematurity complications N9 (N=404)** |  |  |  |  |  |  |
|  | *Low birth weight* |  |  |  |  |  |  |
|  | Extremely low birth weight | P07.0 | 179 (12.3) | 64 (10.7) | 74 (12.5) | 41 (15.2) |  |
|  | Other low birth weight | P07.1 | 174 (11.9) | 33 (5.5) | 78 (13.2) | 63 (23.4) |  |
|  | *Prematurity* |  |  |  |  |  |  |
|  | Extreme immaturity | P07.2 | 17 (1.2) | 7 (1.2) | 8 (1.3) | 2 (0.7) |  |
|  | Other preterm infants | P07.3 | 34 (2.3) | 12 (2.0) | 18 (3.0) | 4 (1.5) |  |
|  | **Miscellaneous N10 (N=3)** |  |  |  |  |  |  |
|  | Encephalopathy, unspecified | G93.4 | 1 (0.1) | 0 (0) | 1 (0.2) | 0 (0) |  |
|  | Asphyxiation | T71 | 1 (0.1) | 0 (0) | 0 (0) | 1 (0.4) |  |
|  | Accidental suffocation and strangulation in bed | W75 | 1 (0.1) | 0 (0) | 1 (0.2) | 0 (0) |  |
|  | **Unspecified condition N11** |  |  |  |  |  |  |
|  | Other ill-defined and unspecified causes of mortality | R99 | 21 (1.4) | 9 (1.5) | 10 (1.7) | 2 (0.7) |  |
