# Supplementary material for: Causes of death identified in neonates enrolled through Child Health and Mortality Prevention Surveillance (CHAMPS), December 2016 –December 2021
Source: PLOS Glob Public Health. 2023 Mar 20;3(3):e0001612. doi: 10.1371/journal.pgph.0001612 (PMC10027211; doi:10.1371/journal.pgph.0001612)
Supplement: S4 Table — (DOCX) [file pgph.0001612.s005.docx]

Supplemental table 4: Underlying cause of the death, stratified by HIV exposure status

|  | HIV exposed  N=290 | Not HIV exposed  N=1168 |
| --- | --- | --- |
| Congenital malformations, deformations & chromosomal abnormalities N1 | 21 (7.2) | 97 (8.3) |
| Disorders related to fetal growth N2 | 3 (1.0) | 8 (0.7) |
| Birth trauma N3 | 0 (0) | 1 (0.1) |
| Complications of intrapartum events N4 | 63 (21.7) | 383 (32.8) |
| Convulsion and disorder of cerebral status N5 | 1 (0.3) | 15 (1.3) |
| Infections N6 | 56 (19.3) | 198 (17.0) |
| Respiratory and cardiovascular disorders N7 | 26 (9.0) | 133 (11.4) |
| Other neonatal conditions N8 | 4 (1.4) | 21 (1.8) |
| Low birth weight/prematurity complications N9 | 114 (39.3) | 290 (24.8) |
| Miscellaneous N10 | 0 (0) | 3 (0.3) |
| Unspecified condition N11 | 2 (0.7) | 19 (1.6) |
