# Supplementary material for: Causes of death identified in neonates enrolled through Child Health and Mortality Prevention Surveillance (CHAMPS), December 2016 –December 2021
Source: PLOS Glob Public Health. 2023 Mar 20;3(3):e0001612. doi: 10.1371/journal.pgph.0001612 (PMC10027211; doi:10.1371/journal.pgph.0001612)
Supplement: S5 Table — (DOCX) [file pgph.0001612.s006.docx]

| **Supplemental Table 5. Underlying causes of death for newborn deaths enrolled in CHAMPS, by site and WHO ICD 10 Perinatal Mortality (PM) category (2016-2021)** | | | | | | | | | |
| --- | --- | --- | --- | --- | --- | --- | --- | --- | --- |
| Underlying Cause of Death |  | All | Bangladesh | Ethiopia | Kenya | Mali | Mozambique | Sierra Leone | South Africa |
|  |  | (N=1458) | (N=180) | (N=110) | (N=181) | (N=74) | (N=306) | (N=144) | (N=463) |
|  | ICD=10 | n (%) | n (%) | n (%) | n (%) | n (%) | n (%) | n (%) | n (%) |
| **Congenital malformations, deformations & chromosomal abnormalities N1** | **Q00-Q99** | **118 (8.1)** | **3 (1.7)** | **9 (8.2)** | **7 (3.9)** | **18 (24.3)** | **23 (7.5)** | **6 (4.2)** | **52 (11.2)** |
| Congenital malformation of nervous system | Q00-Q07 | 21 (1.4) | 1 (0.6) | 4 (3.6) | 2 (1.1) | 0 (0) | 4 (1.3) | 1 (0.7) | 9 (1.9) |
| Congenital malformations of circulatory system | Q20-Q28 | 12 (0.8) | 0 (0) | 0 (0) | 4 (2.2) | 1 (1.4) | 0 (0) | 0 (0) | 7 (1.5) |
| Congenital malformation of digestive system | Q38-Q45 | 5 (0.3) | 0 (0) | 0 (0) | 0 (0) | 0 (0) | 4 (1.3) | 0 (0) | 1 (0.2) |
| Congenital malformation of urinary system | Q60-Q64 | 7 (0.5) | 1 (0.6) | 1 (0.9) | 0 (0) | 0 (0) | 0 (0) | 1 (0.7) | 4 (0.9) |
| Congenital malformations & deformations of musculoskeletal system | Q65-Q79 | 29 (2.0) | 1 (0.6) | 1 (0.9) | 0 (0) | 4 (5.4) | 9 (2.9) | 1 (0.7) | 13 (2.8) |
| Other congenital malformations | Q18, Q33, Q80-Q89, D48 | 24 (1.6) | 0 (0) | 2 (1.8) | 1 (0.6) | 6 (8.1) | 4 (1.3) | 3 (2.1) | 8 (1.7) |
| Chromosomal abnormalities, not elsewhere classified | Q90-Q99 | 20 (1.4) | 0 (0) | 1 (0.9) | 0 (0) | 7 (9.5) | 2 (0.7) | 0 (0) | 10 (2.2) |
| Congenital myopathies | G71.2 | 1 (0.1) | 0 (0) | 0 (0) | 0 (0) | 0 (0) | 0 (0) | 0 (0) | 1 (0.2) |
| **Disorders related to fetal growth N2** | **P05** | **11 (0.8)** | **2 (1.1)** | **2 (1.8)** | **2 (1.1)** | **2 (2.7)** | **0 (0)** | **0 (0)** | **3 (0.6)** |
| **Birth trauma N3** | **P15** | **1 (0.1)** | **0 (0)** | **0 (0)** | **0 (0)** | **1 (1.4)** | **0 (0)** | **0 (0)** | **0 (0)** |
| **Complications of intrapartum events N4** | **P20, P21, P02, O14.2** | **446 (30.6)** | **75 (41.7)** | **35 (31.8)** | **62 (34.3)** | **11 (14.9)** | **117 (38.2)** | **74 (51.4)** | **72 (15.6)** |
| Intrauterine hypoxia | P20 | 233 (16.0) | 39 (21.7) | 11 (10.0) | 23 (12.7) | 6 (8.1) | 49 (16.0) | 41 (28.5) | 64 (13.8) |
| Birth asphyxia | P21 | 207 (14.2) | 36 (20.0) | 24 (21.8) | 38 (21.0) | 5 (6.8) | 67 (21.9) | 33 (22.9) | 4 (0.9) |
| Complications of placenta, cord and membranes | P02 | 5 (0.3) | 0 (0) | 0 (0) | 1 (0.6) | 0 (0) | 1 (0.3) | 0 (0) | 3 (0.6) |
| Hemolysis, Elevated Liver enzymes and Low Platelets (HELLP) syndrome | O14.2 | 1 (0.1) | 0 (0) | 0 (0) | 0 (0) | 0 (0) | 0 (0) | 0 (0) | 1 (0.2) |
| **Convulsion and disorder of cerebral status N5** | **P90-P96** | 16 (1.1) | 2 (1.1) | 1 (0.9) | 2 (1.1) | 2 (2.7) | 8 (2.6) | 0 (0) | 1 (0.2) |
| **Infections N6** |  | 254 (17.4) | 13 (7.2) | 15 (13.6) | 47 (26.0) | 9 (12.2) | 77 (25.2) | 29 (20.1) | 64 (13.8) |
| Other gastroenteritis and colitis of infectious and unspecified origin | A09 | 2 (0.1) | 0 (0) | 0 (0) | 0 (0) | 0 (0) | 1 (0.3) | 0 (0) | 1 (0.2) |
| Other bacterial diseases | A40, A41 | 6 (0.4) | 0 (0) | 0 (0) | 5 (2.8) | 1 (1.4) | 0 (0) | 0 (0) | 0 (0) |
| Congenital syphilis | A50 | 7 (0.5) | 0 (0) | 0 (0) | 0 (0) | 0 (0) | 5 (1.6) | 0 (0) | 2 (0.4) |
| Viral infection of central nervous system | A86 | 1 (0.1) | 0 (0) | 0 (0) | 1 (0.6) | 0 (0) | 0 (0) | 0 (0) | 0 (0) |
| Viral infections characterized by skin and mucous membrane lesions | B00-B09 | 1 (0.1) | 1 (0.6) | 0 (0) | 0 (0) | 0 (0) | 0 (0) | 0 (0) | 0 (0) |
| Human immunodeficiency virus | B20-B24 | 1 (0.1) | 0 (0) | 0 (0) | 1 (0.6) | 0 (0) | 0 (0) | 0 (0) | 0 (0) |
| Other viral diseases | B33.8 | 1 (0.1) | 0 (0) | 0 (0) | 0 (0) | 0 (0) | 0 (0) | 0 (0) | 1 (0.2) |
| Bacterial meningitis | G00, G03.9 | 5 (0.3) | 0 (0) | 0 (0) | 0 (0) | 0 (0) | 2 (0.7) | 0 (0) | 3 (0.6) |
| Bacterial & viral pneumonia | J12, J15, J16, J18 | 28 (1.9) | 0 (0) | 4 (3.6) | 10 (5.5) | 3 (4.1) | 7 (2.3) | 1 (0.7) | 3 (0.6) |
| Congenital pneumonia | P23 | 33 (2.3) | 0 (0) | 1 (0.9) | 0 (0) | 1 (1.4) | 21 (6.9) | 5 (3.5) | 5 (1.1) |
| Infection related to perinatal period | P35-P39 | 166 (11.4) | 11 (6.1) | 10 (9.1) | 28 (15.5) | 4 (5.4) | 41 (13.4) | 23 (16.0) | 49 (10.6) |
| COVID-19 | U07.1 | 2 (0.1) | 1 (0.6) | 0 (0) | 1 (0.6) | 0 (0) | 0 (0) | 0 (0) | 0 (0) |
| Other infectious or parasitic diseases | P00.2 | 1 (0.1) | 0 (0) | 0 (0) | 1 (0.6) | 0 (0) | 0 (0) | 0 (0) | 0 (0) |
| **Respiratory and cardiovascular disorders N7** | **P20-P29** | **159 (10.9)** | **9 (5.0)** | **28 (25.5)** | **42 (23.2)** | **5 (6.8)** | **61 (19.9)** | **12 (8.3)** | **2 (0.4)** |
| Respiratory distress syndrome of newborn | P22 | 125 (8.6) | 9 (5.0) | 20 (18.2) | 30 (16.6) | 3 (4.1) | 54 (17.6) | 9 (6.2) | 0 (0) |
| Neonatal aspiration syndromes | P24 | 30 (2.1) | 0 (0) | 8 (7.3) | 12 (6.6) | 2 (2.7) | 4 (1.3) | 3 (2.1) | 1 (0.2) |
| Pulmonary haemorrhage originating in the perinatal period | P26 | 3 (0.2) | 0 (0) | 0 (0) | 0 (0) | 0 (0) | 3 (1.0) | 0 (0) | 0 (0) |
| Other respiratory conditions originating in the perinatal period | P28 | 1 (0.1) | 0 (0) | 0 (0) | 0 (0) | 0 (0) | 0 (0) | 0 (0) | 1 (0.2) |
| **Other neonatal conditions N8** | **E41,E87.0, E88.9, K76.9,P03.4,P51.0, P52.4,P55.1, P55.9,P56.9, P57.9,P59.0,P59.2, P70.1,P76.9,P77, P83.2, P83.3,R10,R62.8** | **25 (1.7)** | **1 (0.6)** | **2 (1.8)** | **4 (2.2)** | **6 (8.1)** | **4 (1.3)** | **0 (0)** | **8 (1.7)** |
| **Low birth weight/prematurity complications N9** | **P07** | **404 (27.7)** | **74 (41.1)** | **17 (15.5)** | **11 (6.1)** | **14 (18.9)** | **13 (4.2)** | **16 (11.1)** | **259 (55.9)** |
| Low birth weight | P07.0-P07.1 | 353 (24.2) | 48 (26.7) | 5 (4.5) | 11 (6.1) | 14 (18.9) | 12 (3.9) | 4 (2.8) | 259 (55.9) |
| Prematurity | P07.2-P07.3 | 51 (3.5) | 26 (14.4) | 12 (10.9) | 0 (0) | 0 (0) | 1 (0.3) | 12 (8.3) | 0 (0) |
| **Miscellaneous N10** | **T71, W75, G93.4** | **3 (0.2)** | **0 (0)** | **1 (0.9)** | **0 (0)** | **1 (1.4)** | **0 (0)** | **0 (0)** | **1 (0.2)** |
| **Unspecified condition N11** | **R99** | **21 (1.4)** | **1 (0.6)** | **0 (0)** | **4 (2.2)** | **5 (6.8)** | **3 (1.0)** | **7 (4.9)** | **1 (0.2)** |
