# Supplementary material for: Causes of death identified in neonates enrolled through Child Health and Mortality Prevention Surveillance (CHAMPS), December 2016 –December 2021
Source: PLOS Glob Public Health. 2023 Mar 20;3(3):e0001612. doi: 10.1371/journal.pgph.0001612 (PMC10027211; doi:10.1371/journal.pgph.0001612)
Supplement: S6 Table — b Maternal conditions identified for CHAMPS deaths that occurred in the neonatal period, by WHO ICD 10 PM underlying cause of the death for the newborn, 2016–2021. Bold text is groupings for underlying causes of death in neonates according to WHO ICD PM and Italic text shows the associated maternal conditions found in those deaths. (ZIP) [file pgph.0001612.s007.zip › S6a_Table.docx]

| **Supplemental Table 6a: Main Maternal condition attributed for neonatal deaths** | | | | | |
| --- | --- | --- | --- | --- | --- |
|  | ICD-10 codes | Total (N=1458) | 24 hours death (N=596) | END (N=593) | LND (N=269) |
| **M1: Complications of Placenta, cord and membranes** |  | **157 (10.8)** | **81 (13.6)** | **58 (9.8)** | **18 (6.7)** |
| Placental complications | O44.1, O45.9, P02.0, P02.1, P02.2, P02.3 | 84 (5.8) | 38 (6.4) | 35 (5.9) | 11 (4.1) |
| Chorioamnionitis and membrane complications | P02.7, P02.8 | 48 (3.3) | 26 (4.4) | 16 (2.7) | 6 (2.2) |
| Umbilical cord complications | P02.4, P02.5 | 24 (1.6) | 16 (2.7) | 7 (1.2) | 1 (0.4) |
| Prolapsed cord | O69.0 | 1 (0.1) | 1 (0.2) | 0 (0) | 0 (0) |
| **M2: Maternal complications of pregnacy** |  | **202 (13.9)** | **91 (15.3)** | **78 (13.2)** | **33 (12.3)** |
| Multiple gestation | O30.0, O30.1, P01.5 | 99 (6.8) | 40 (6.7) | 39 (6.6) | 20 (7.4) |
| Premature Rupture of membranes | O42.1, O42.9, P01.1 | 61 (4.2) | 25 (4.2) | 29 (4.9) | 7 (2.6) |
| Cervical insufficiency and pelvic anomalies | P01.0 | 17 (1.2) | 12 (2.0) | 3 (0.5) | 2 (0.7) |
| Other labor and delivery complications | O46.9, O48 | 11 (0.8) | 6 (1.0) | 5 (0.8) | 0 (0) |
| Other Maternal factor | P01.8 | 2 (0.1) | 2 (0.3) | 0 (0) | 0 (0) |
| Uterine fluid disorders | P01.2, P01.3 | 8 (0.5) | 5 (0.8) | 2 (0.3) | 1 (0.4) |
| Chorioamnionitis and membrane complications | O41.1 | 1 (0.1) | 0 (0) | 0 (0) | 1 (0.4) |
| **M3: Other complications of labour and delivery** |  | **236 (16.2)** | **141 (23.7)** | **85 (14.3)** | **10 (3.7)** |
| Other labor and delivery complications | O63, O63.1, O63.9, P03.3, P03.5, P03.6, P03.8, P03.9 | 112 (7.7) | 65 (10.9) | 44 (7.4) | 3 (1.1) |
| Obstructed labor and fetal malpresentation | O64.1, O65.4, P03.0, P03.1 | 67 (4.6) | 42 (7.0) | 24 (4.0) | 1 (0.4) |
| Preterm labor or delivery | O60.1 | 43 (2.9) | 25 (4.2) | 12 (2.0) | 6 (2.2) |
| Other neonatal disorders | P08.2 | 5 (0.3) | 3 (0.5) | 2 (0.3) | 0 (0) |
| Uterine rupture | O71.1 | 4 (0.3) | 2 (0.3) | 2 (0.3) | 0 (0) |
| Obstructed labor | O66, O66.9 | 5 (0.3) | 4 (0.7) | 1 (0.2) | 0 (0) |
| **M4: Maternal medical and surgical conditions** |  | **233 (16.0)** | **89 (14.9)** | **105 (17.7)** | **39 (14.5)** |
| Maternal hypertension | P00.0, O10, O10.0, O10.9, O11, O13, O14, O14.0, O14.1, O14.2, O14.9, O15, O15.0 | 149 (10.2) | 49 (8.2) | 76 (12.8) | 24 (8.9) |
| Other maternal factors | O34.1, P00.1, P00.8, P00.9, Z35.6 | 24 (1.6) | 12 (2.0) | 4 (0.7) | 8 (3.0) |
| Maternal infections | P00.2, O98.0 | 22 (1.5) | 11 (1.8) | 9 (1.5) | 2 (0.7) |
| Other infections | A32.9, B25.9 | 7 (0.5) | 1 (0.2) | 4 (0.7) | 2 (0.7) |
| HIV | B24, O98.7, Z20.6 | 9 (0.6) | 3 (0.5) | 5 (0.8) | 1 (0.4) |
| Maternal diabetes | O24.0, O24.1, O24.9 | 7 (0.5) | 2 (0.3) | 3 (0.5) | 2 (0.7) |
| Syphilis | A51.9, A53.9 | 4 (0.3) | 2 (0.3) | 2 (0.3) | 0 (0) |
| Maternal injury and accident | P00.5 | 1 (0.1) | 1 (0.2) | 0 (0) | 0 (0) |
| Anemias | D64.9 | 1 (0.1) | 1 (0.2) | 0 (0) | 0 (0) |
| Maternal nutritional disorder | P00.4 | 1 (0.1) | 0 (0) | 1 (0.2) | 0 (0) |
| Other nutritional deficiencies | E53.8 | 1 (0.1) | 1 (0.2) | 0 (0) | 0 (0) |
| Sepsis | A41.8 | 1 (0.1) | 1 (0.2) | 0 (0) | 0 (0) |
| Other | Z22.3 | 5 (0.3) | 4 (0.7) | 1 (0.2) | 0 (0) |
| **M5: No maternal condition** |  | **585 (40.1)** | **180 (30.2)** | **245 (41.3)** | **160 (59.5)** |
| **Other** |  | **45 (3.1)** | **14 (2.3)** | **22 (3.7)** | **9 (3.3)** |
