# Supplementary material for: Causes of death identified in neonates enrolled through Child Health and Mortality Prevention Surveillance (CHAMPS), December 2016 –December 2021
Source: PLOS Glob Public Health. 2023 Mar 20;3(3):e0001612. doi: 10.1371/journal.pgph.0001612 (PMC10027211; doi:10.1371/journal.pgph.0001612)
Supplement: S6 Table — b Maternal conditions identified for CHAMPS deaths that occurred in the neonatal period, by WHO ICD 10 PM underlying cause of the death for the newborn, 2016–2021. Bold text is groupings for underlying causes of death in neonates according to WHO ICD PM and Italic text shows the associated maternal conditions found in those deaths. (ZIP) [file pgph.0001612.s007.zip › S6b_Table.docx]

Table 6b: Maternal conditions identified for CHAMPS deaths that occurred in the neonatal period, by WHO ICD 10 PM underlying cause of the death for the newborn, 2016-2021. **Bold text** is groupings for underlying causes of death in neonates according to WHO ICD PM and *Italic text* shows the associated maternal conditions found in those deaths.

|  | ICD 10 | Total  (N=1,458) n (%) | Death in first 24 hours  (N=596)  n (%) | Early Neonate (24 hours - <7 days)  (N=593)  n (%) | Late Neonate (7-27 days)  (N=269)  n (%) |
| --- | --- | --- | --- | --- | --- |
| **N1: Congenital malformation** |  | **118 (8.1)** | **46 (7.7)** | **41 (6.9)** | **31 (11.5)** |
| Anemias | D64.9 | 2 (0.1) | 2 (0.3) | 0 (0) | 0 (0) |
| Cesarean delivery | P03.4 | 1 (0.1) | 1 (0.2) | 0 (0) | 0 (0) |
| Chorioamnionitis and membrane complications | P02.7 | 1 (0.1) | 1 (0.2) | 0 (0) | 0 (0) |
| HIV | B24 | 18 (1.2) | 7 (1.2) | 6 (1.0) | 5 (1.9) |
| HIV exposure | Z20.6 | 1 (0.1) | 1 (0.2) | 0 (0) | 0 (0) |
| Maternal hypertension | O10.0, O13, O15, P00.0 | 4 (0.3) | 2 (0.3) | 1 (0.2) | 1 (0.4) |
| Maternal medication or toxic exposure | P04.1, T65.9 | 2 (0.1) | 1 (0.2) | 0 (0) | 1 (0.4) |
| Multiple gestation | O30.0, P01.5 | 2 (0.1) | 1 (0.2) | 1 (0.2) | 0 (0) |
| Neonatal preterm birth complications | P07.1 | 1 (0.1) | 0 (0) | 1 (0.2) | 0 (0) |
| Obstructed labor | O66.9 | 1 (0.1) | 1 (0.2) | 0 (0) | 0 (0) |
| Obstructed labor and fetal malpresentation | P03.0, P03.1 | 4 (0.3) | 4 (0.7) | 0 (0) | 0 (0) |
| Other labor and delivery complications | O48, O63.9, P03.8 | 4 (0.3) | 2 (0.3) | 2 (0.3) | 0 (0) |
| Other maternal factor | O04.4, P00.8, Z35.3, Z35.4, Z35.5, Z35.6, Z87.5 | 16 (1.1) | 5 (0.8) | 6 (1.0) | 5 (1.9) |
| Other neurological disorders | G40.9 | 1 (0.1) | 0 (0) | 0 (0) | 1 (0.4) |
| Other nutritional deficiencies | E53.8 | 1 (0.1) | 1 (0.2) | 0 (0) | 0 (0) |
| Placental complications | P02.1 | 3 (0.2) | 2 (0.3) | 1 (0.2) | 0 (0) |
| Premature Rupture of membranes | O42.1 | 1 (0.1) | 0 (0) | 1 (0.2) | 0 (0) |
| Preterm labor or delivery | O60.1 | 19 (1.3) | 6 (1.0) | 5 (0.8) | 8 (3.0) |
| Umbilical cord complications | P02.5 | 1 (0.1) | 1 (0.2) | 0 (0) | 0 (0) |
| Uterine fluid disorders | P01.2 | 1 (0.1) | 0 (0) | 1 (0.2) | 0 (0) |
| **N2: Disorders related to fetal growth** |  | **11 (0.8)** | **4 (0.7)** | **4 (0.7)** | **3 (1.1)** |
| Heart Diseases | I67.6 | 1 (0.1) | 0 (0) | 0 (0) | 1 (0.4) |
| HIV | B24, Z21 | 3 (0.2) | 2 (0.3) | 0 (0) | 1 (0.4) |
| Maternal hypertension | O10, O14.2, P00.0 | 4 (0.3) | 1 (0.2) | 2 (0.3) | 1 (0.4) |
| Other labor and delivery complications | P03.8 | 1 (0.1) | 0 (0) | 0 (0) | 1 (0.4) |
| Other maternal factor | P00.8, Z35.3 | 2 (0.1) | 0 (0) | 0 (0) | 2 (0.7) |
| Placental complications | O43.8, P02.2 | 2 (0.1) | 2 (0.3) | 0 (0) | 0 (0) |
| Premature Rupture of membranes | O42.9 | 1 (0.1) | 1 (0.2) | 0 (0) | 0 (0) |
| Preterm labor or delivery | O60.1 | 3 (0.2) | 1 (0.2) | 1 (0.2) | 1 (0.4) |
| Uterine fluid disorders | P01.2 | 1 (0.1) | 1 (0.2) | 0 (0) | 0 (0) |
| **N3: Birth trauma** |  | **1 (0.1)** | **0 (0)** | **1 (0.2)** | **0 (0)** |
| Malpresentation before labor | P01.7 | 1 (0.1) | 0 (0) | 1 (0.2) | 0 (0) |
| Maternal medication or toxic exposure | T65.9 | 1 (0.1) | 0 (0) | 1 (0.2) | 0 (0) |
| Other maternal factor | Z87.5 | 1 (0.1) | 0 (0) | 1 (0.2) | 0 (0) |
| **N4: Complications of intrapartum events** |  | **446 (30.6)** | **252 (42.3)** | **176 (29.7)** | **18 (6.7)** |
| Anemias | D64.9 | 5 (0.3) | 3 (0.5) | 2 (0.3) | 0 (0) |
| Cervical insufficiency and pelvic anomalies | P01.0 | 2 (0.1) | 2 (0.3) | 0 (0) | 0 (0) |
| Cesarean delivery | O82.1, P03.4 | 5 (0.3) | 3 (0.5) | 1 (0.2) | 1 (0.4) |
| Chorioamnionitis and membrane complications | P02.7, P02.8 | 18 (1.2) | 14 (2.3) | 4 (0.7) | 0 (0) |
| Heart Diseases | I33.0, I50.9 | 2 (0.1) | 0 (0) | 2 (0.3) | 0 (0) |
| HIV | B24, O98.7, Z21 | 50 (3.4) | 26 (4.4) | 21 (3.5) | 3 (1.1) |
| Malaria | B50, B50.9 | 2 (0.1) | 1 (0.2) | 1 (0.2) | 0 (0) |
| Malpresentation before labor | P01.7 | 5 (0.3) | 3 (0.5) | 2 (0.3) | 0 (0) |
| Maternal circulatory and respiratory diseases | O99.4, P00.3 | 2 (0.1) | 1 (0.2) | 1 (0.2) | 0 (0) |
| Maternal diabetes | O24.9 | 2 (0.1) | 1 (0.2) | 1 (0.2) | 0 (0) |
| Maternal hypertension | O10, O11, O13, O14, O14.2, O14.9, O15, O16, P00.0 | 73 (5.0) | 36 (6.0) | 35 (5.9) | 2 (0.7) |
| Maternal infection | O23, O23.4, P00.2 | 23 (1.6) | 14 (2.3) | 9 (1.5) | 0 (0) |
| Maternal injury and accident | P00.5 | 1 (0.1) | 1 (0.2) | 0 (0) | 0 (0) |
| Maternal medication or toxic exposure | T65.9 | 3 (0.2) | 2 (0.3) | 1 (0.2) | 0 (0) |
| Maternal nutritional disorders | P00.4 | 4 (0.3) | 3 (0.5) | 1 (0.2) | 0 (0) |
| Multiple gestation | O30.0, P01.5, Z64.1 | 36 (2.5) | 26 (4.4) | 7 (1.2) | 3 (1.1) |
| Obstructed labor | O66.9 | 2 (0.1) | 2 (0.3) | 0 (0) | 0 (0) |
| Obstructed labor and fetal malpresentation | O64.1, O65.4, P03.0, P03.1 | 71 (4.9) | 47 (7.9) | 23 (3.9) | 1 (0.4) |
| Other | F32.9, R50.9, U07.1, Y66 | 4 (0.3) | 2 (0.3) | 2 (0.3) | 0 (0) |
| Other labor and delivery complications | O46.9, O48, O62.0, O63, O63.1, O63.2, O63.9, O75.6, O80.1, P03.2, P03.3, P03.5, P03.6, P03.8, P03.9 | 102 (7.0) | 61 (10.2) | 39 (6.6) | 2 (0.7) |
| Other maternal factor | K04.9, O05, O99.0, P00.1, P00.8, Z35.3, Z35.4, Z35.5, Z35.6, Z35.9, Z38.1, Z64.0, Z84.3, Z87.5 | 56 (3.8) | 41 (6.9) | 14 (2.4) | 1 (0.4) |
| Other neonatal disorders | P08.2 | 5 (0.3) | 3 (0.5) | 2 (0.3) | 0 (0) |
| Placental complications | O45.9, P02.0, P02.1, P02.2 | 32 (2.2) | 22 (3.7) | 10 (1.7) | 0 (0) |
| Premature Rupture of membranes | O42, O42.0, O42.1, O42.9, P01.1 | 24 (1.6) | 17 (2.9) | 6 (1.0) | 1 (0.4) |
| Preterm labor or delivery | O60.1 | 39 (2.7) | 26 (4.4) | 12 (2.0) | 1 (0.4) |
| Prolapsed cord | O69.0 | 1 (0.1) | 1 (0.2) | 0 (0) | 0 (0) |
| Sepsis | A41.9 | 2 (0.1) | 0 (0) | 2 (0.3) | 0 (0) |
| Syphilis | A53.9, O98.1 | 3 (0.2) | 1 (0.2) | 2 (0.3) | 0 (0) |
| Umbilical cord complications | P02.4, P02.5 | 22 (1.5) | 16 (2.7) | 6 (1.0) | 0 (0) |
| Undetermined | R69 | 2 (0.1) | 2 (0.3) | 0 (0) | 0 (0) |
| Uterine fluid disorders | P01.2, P01.3 | 9 (0.6) | 7 (1.2) | 2 (0.3) | 0 (0) |
| Uterine rupture | O71.1 | 3 (0.2) | 1 (0.2) | 2 (0.3) | 0 (0) |
| **N5: Convulsion and disorder of cerebral disorders** |  | **16 (1.1)** | **6 (1.0)** | **8 (1.3)** | **2 (0.7)** |
| HIV | B24 | 1 (0.1) | 1 (0.2) | 0 (0) | 0 (0) |
| Maternal hypertension | O14 | 1 (0.1) | 1 (0.2) | 0 (0) | 0 (0) |
| Maternal medication or toxic exposure | T65.9 | 1 (0.1) | 0 (0) | 1 (0.2) | 0 (0) |
| Multiple gestation | P01.5 | 1 (0.1) | 1 (0.2) | 0 (0) | 0 (0) |
| Obstructed labor and fetal malpresentation | P03.0 | 1 (0.1) | 0 (0) | 1 (0.2) | 0 (0) |
| Other labor and delivery complications | O48, O63.1, P03.8 | 5 (0.3) | 2 (0.3) | 3 (0.5) | 0 (0) |
| Other maternal factor | Z35.6, Z87.5 | 2 (0.1) | 2 (0.3) | 0 (0) | 0 (0) |
| Umbilical cord complications | P02.5 | 2 (0.1) | 2 (0.3) | 0 (0) | 0 (0) |
| **N6: Infection** |  | **254 (17.4)** | **75 (12.6)** | **100 (16.9)** | **79 (29.4)** |
| Anemias | D64.9 | 2 (0.1) | 2 (0.3) | 0 (0) | 0 (0) |
| Cervical insufficiency and pelvic anomalies | P01.0 | 4 (0.3) | 4 (0.7) | 0 (0) | 0 (0) |
| Cesarean delivery | O82.1, O82.8, P03.4 | 3 (0.2) | 2 (0.3) | 1 (0.2) | 0 (0) |
| Chorioamnionitis and membrane complications | O41.1, P02.7 | 29 (2.0) | 17 (2.9) | 9 (1.5) | 3 (1.1) |
| Congenital birth defects | Q42.9 | 1 (0.1) | 0 (0) | 0 (0) | 1 (0.4) |
| HIV | B20, B23.0, B24, B24.0, O98.7, Z21 | 43 (2.9) | 11 (1.8) | 16 (2.7) | 16 (5.9) |
| HIV exposure | Z20.6 | 2 (0.1) | 0 (0) | 0 (0) | 2 (0.7) |
| Maternal circulatory and respiratory diseases | P00.3 | 2 (0.1) | 1 (0.2) | 1 (0.2) | 0 (0) |
| Maternal diabetes | O24.0, O24.1, O24.9 | 4 (0.3) | 3 (0.5) | 1 (0.2) | 0 (0) |
| Maternal hypertension | O10.9, O11, O13, O14.1, O14.9, P00.0 | 18 (1.2) | 10 (1.7) | 7 (1.2) | 1 (0.4) |
| Maternal infection | P00.2, Z22.4 | 16 (1.1) | 5 (0.8) | 8 (1.3) | 3 (1.1) |
| Maternal nutritional disorders | P00.4 | 1 (0.1) | 1 (0.2) | 0 (0) | 0 (0) |
| Multiple gestation | O30.0, O30.1, P01.5 | 21 (1.4) | 6 (1.0) | 9 (1.5) | 6 (2.2) |
| Obesity | E66.9 | 1 (0.1) | 1 (0.2) | 0 (0) | 0 (0) |
| Obstructed labor | O66 | 1 (0.1) | 0 (0) | 1 (0.2) | 0 (0) |
| Obstructed labor and fetal malpresentation | P03.0, P03.1 | 9 (0.6) | 4 (0.7) | 5 (0.8) | 0 (0) |
| Other | Z22.3 | 5 (0.3) | 4 (0.7) | 1 (0.2) | 0 (0) |
| Other infections | A32.9, A97.9, B25.9 | 8 (0.5) | 1 (0.2) | 4 (0.7) | 3 (1.1) |
| Other labor and delivery complications | O48, O63.9, P03.3, P03.8, Z38.4 | 21 (1.4) | 13 (2.2) | 8 (1.3) | 0 (0) |
| Other maternal factor | P00.1, P00.7, P00.8, Z35.3, Z35.5, Z35.6, Z38.1, Z87.5 | 33 (2.3) | 14 (2.3) | 17 (2.9) | 2 (0.7) |
| Other neonatal disorders | P08.2 | 1 (0.1) | 0 (0) | 1 (0.2) | 0 (0) |
| Placental complications | O44.0, O45.9, P02.1 | 6 (0.4) | 2 (0.3) | 4 (0.7) | 0 (0) |
| Premature Rupture of membranes | O42.1, O42.2, O42.9, P01.1 | 25 (1.7) | 8 (1.3) | 15 (2.5) | 2 (0.7) |
| Preterm labor or delivery | O60.1 | 44 (3.0) | 22 (3.7) | 14 (2.4) | 8 (3.0) |
| Sepsis | A41.9 | 2 (0.1) | 2 (0.3) | 0 (0) | 0 (0) |
| Spontaneous abortion | O03 | 1 (0.1) | 1 (0.2) | 0 (0) | 0 (0) |
| Syphilis | A51.9, A53.9 | 5 (0.3) | 3 (0.5) | 2 (0.3) | 0 (0) |
| Umbilical cord complications | P02.4 | 2 (0.1) | 1 (0.2) | 1 (0.2) | 0 (0) |
| Undetermined | R69 | 2 (0.1) | 0 (0) | 2 (0.3) | 0 (0) |
| Uterine fluid disorders | P01.2, P01.3 | 2 (0.1) | 0 (0) | 2 (0.3) | 0 (0) |
| **N7: Respiratory and cardiovascular disorders** |  | **159 (10.9)** | **80 (13.4)** | **66 (11.1)** | **13 (4.8)** |
| Anemias | D64.9, D69.6 | 10 (0.7) | 6 (1.0) | 4 (0.7) | 0 (0) |
| Cervical insufficiency and pelvic anomalies | P01.0 | 1 (0.1) | 1 (0.2) | 0 (0) | 0 (0) |
| Cesarean delivery | P03.4 | 1 (0.1) | 0 (0) | 0 (0) | 1 (0.4) |
| Diabetes | E10.1 | 1 (0.1) | 1 (0.2) | 0 (0) | 0 (0) |
| Heart Diseases | I50 | 1 (0.1) | 0 (0) | 1 (0.2) | 0 (0) |
| HIV | B24, O98.7, Z21 | 21 (1.4) | 7 (1.2) | 11 (1.9) | 3 (1.1) |
| Liver disease | K74, K76.6 | 2 (0.1) | 2 (0.3) | 0 (0) | 0 (0) |
| Malaria | B50.8 | 1 (0.1) | 0 (0) | 1 (0.2) | 0 (0) |
| Maternal diabetes | O24.1, O24.9 | 2 (0.1) | 2 (0.3) | 0 (0) | 0 (0) |
| Maternal hypertension | O14.0, O15.0, O16, P00.0 | 16 (1.1) | 8 (1.3) | 8 (1.3) | 0 (0) |
| Maternal infection | P00.2 | 5 (0.3) | 3 (0.5) | 2 (0.3) | 0 (0) |
| Maternal medication or toxic exposure | T65.9 | 3 (0.2) | 2 (0.3) | 1 (0.2) | 0 (0) |
| Multiple gestation | O30.0, O30.1, O30.9, P01.5 | 44 (3.0) | 25 (4.2) | 16 (2.7) | 3 (1.1) |
| Neonatal preterm birth complications | P07.0, P07.1, P22.0 | 3 (0.2) | 3 (0.5) | 0 (0) | 0 (0) |
| Obstructed labor | O66.9 | 1 (0.1) | 1 (0.2) | 0 (0) | 0 (0) |
| Obstructed labor and fetal malpresentation | P03.0, P03.1 | 6 (0.4) | 3 (0.5) | 2 (0.3) | 1 (0.4) |
| Other | T74.2, Z53.2 | 2 (0.1) | 2 (0.3) | 0 (0) | 0 (0) |
| Other labor and delivery complications | O48, O61.9, O63.1, O63.9, O75.6, P03.3, P03.6, P03.8 | 30 (2.1) | 25 (4.2) | 4 (0.7) | 1 (0.4) |
| Other maternal factor | N96, P00.1, P00.8, P00.9, Z35.3, Z35.6, Z35.9, Z38.1 | 20 (1.4) | 12 (2.0) | 7 (1.2) | 1 (0.4) |
| Other neonatal disorders | P08.2 | 1 (0.1) | 0 (0) | 1 (0.2) | 0 (0) |
| Placental complications | O44.1, P02.1, P02.2 | 14 (1.0) | 7 (1.2) | 5 (0.8) | 2 (0.7) |
| Premature Rupture of membranes | O42.1, O42.9, P01.1 | 18 (1.2) | 10 (1.7) | 7 (1.2) | 1 (0.4) |
| Preterm labor or delivery | O60.1 | 11 (0.8) | 8 (1.3) | 3 (0.5) | 0 (0) |
| Umbilical cord complications | P02.5 | 2 (0.1) | 1 (0.2) | 1 (0.2) | 0 (0) |
| Uterine fluid disorders | P01.3 | 1 (0.1) | 1 (0.2) | 0 (0) | 0 (0) |
| **N8: Other neonatal conditions** |  | **25 (1.7)** | **8 (1.3)** | **7 (1.2)** | **10 (3.7)** |
| Cesarean delivery | O82.8, P03.4 | 2 (0.1) | 0 (0) | 2 (0.3) | 0 (0) |
| Chorioamnionitis and membrane complications | P02.7 | 1 (0.1) | 1 (0.2) | 0 (0) | 0 (0) |
| HIV | B24 | 4 (0.3) | 2 (0.3) | 0 (0) | 2 (0.7) |
| Maternal hypertension | O10, O13, O14.9 | 5 (0.3) | 1 (0.2) | 2 (0.3) | 2 (0.7) |
| Maternal infection | O24.4 | 1 (0.1) | 0 (0) | 1 (0.2) | 0 (0) |
| Maternal medication or toxic exposure | P04.2, P04.3, T65.9 | 3 (0.2) | 0 (0) | 0 (0) | 3 (1.1) |
| Multiple gestation | P01.5, Z64.1 | 2 (0.1) | 0 (0) | 2 (0.3) | 0 (0) |
| Other labor and delivery complications | O48 | 1 (0.1) | 0 (0) | 1 (0.2) | 0 (0) |
| Other maternal factor | O04, Z35.5, Z35.6, Z87.5 | 5 (0.3) | 2 (0.3) | 2 (0.3) | 1 (0.4) |
| Premature Rupture of membranes | P01.1 | 1 (0.1) | 1 (0.2) | 0 (0) | 0 (0) |
| Preterm labor or delivery | O60.1 | 5 (0.3) | 3 (0.5) | 1 (0.2) | 1 (0.4) |
| Uterine fluid disorders | P01.2, P01.3 | 2 (0.1) | 2 (0.3) | 0 (0) | 0 (0) |
| **N9: Low birth weight/prematurity complication** |  | **404 (27.7)** | **116 (19.5)** | **178 (30.0)** | **110 (40.9)** |
| Anemias | D50, D50.9, D62, D64.9 | 4 (0.3) | 1 (0.2) | 2 (0.3) | 1 (0.4) |
| Cervical insufficiency and pelvic anomalies | O34.3, P01.0 | 12 (0.8) | 6 (1.0) | 3 (0.5) | 3 (1.1) |
| Cesarean delivery | O82.1, O82.8, P03.4 | 4 (0.3) | 0 (0) | 2 (0.3) | 2 (0.7) |
| Chorioamnionitis and membrane complications | P02.7 | 21 (1.4) | 6 (1.0) | 10 (1.7) | 5 (1.9) |
| Congenital birth defects | Q24.9 | 1 (0.1) | 0 (0) | 1 (0.2) | 0 (0) |
| HIV | B20.8, B22.2, B24 | 110 (7.5) | 23 (3.9) | 50 (8.4) | 37 (13.8) |
| Kidney Disease | N17.9, N18.9 | 2 (0.1) | 1 (0.2) | 0 (0) | 1 (0.4) |
| Lower respiratory infections | J18.9 | 1 (0.1) | 0 (0) | 1 (0.2) | 0 (0) |
| Malpresentation before labor | P01.7 | 2 (0.1) | 1 (0.2) | 1 (0.2) | 0 (0) |
| Maternal circulatory and respiratory diseases | P00.3 | 2 (0.1) | 1 (0.2) | 1 (0.2) | 0 (0) |
| Maternal diabetes | O24.0, O24.1, O24.9 | 8 (0.5) | 1 (0.2) | 4 (0.7) | 3 (1.1) |
| Maternal hypertension | O10, O10.1, O10.9, O11, O13, O14, O14.1, O14.2, O14.9, O15, O16, P00.0 | 89 (6.1) | 17 (2.9) | 47 (7.9) | 25 (9.3) |
| Maternal infection | O24.4, O98.0, O98.8, P00.2 | 19 (1.3) | 6 (1.0) | 8 (1.3) | 5 (1.9) |
| Maternal medication or toxic exposure | P04.2, P04.3, P04.4, T65.9 | 7 (0.5) | 2 (0.3) | 2 (0.3) | 3 (1.1) |
| Maternal nutritional disorders | O26.1, P00.4 | 2 (0.1) | 1 (0.2) | 1 (0.2) | 0 (0) |
| Multiple gestation | O30.0, O30.1, P01.5, Z37.3 | 84 (5.8) | 26 (4.4) | 32 (5.4) | 26 (9.7) |
| Obesity | E66.9 | 11 (0.8) | 2 (0.3) | 6 (1.0) | 3 (1.1) |
| Obstructed labor and fetal malpresentation | P03.0 | 10 (0.7) | 2 (0.3) | 5 (0.8) | 3 (1.1) |
| Other | F99, N10, N19, P96.4, R45.6, T74.2, U07.1, Z22.3 | 8 (0.5) | 3 (0.5) | 5 (0.8) | 0 (0) |
| Other endocrine, metabolic, blood, and immune disorders | E03.9 | 1 (0.1) | 0 (0) | 0 (0) | 1 (0.4) |
| Other infections | A63.0, B37.8, N39.0 | 5 (0.3) | 3 (0.5) | 2 (0.3) | 0 (0) |
| Other labor and delivery complications | O75.6, P03.5, P03.8, Z38.4 | 13 (0.9) | 5 (0.8) | 6 (1.0) | 2 (0.7) |
| Other maternal factor | N93.9, O04, O05, O07.4, O26.8, O34.1, O87.1, O90.0, O99.0, P00.1, P00.8, P01.8, Q51.3, Z35.1, Z35.2, Z35.3, Z35.4, Z35.5, Z35.6, Z38.1, Z64.0, Z87.5, Z91.5 | 132 (9.1) | 34 (5.7) | 62 (10.5) | 36 (13.4) |
| Other neonatal disorders | P52.4 | 1 (0.1) | 0 (0) | 1 (0.2) | 0 (0) |
| Other neurological disorders | R56.8 | 1 (0.1) | 1 (0.2) | 0 (0) | 0 (0) |
| Other respiratory disease | I27.2, J45.9 | 2 (0.1) | 0 (0) | 1 (0.2) | 1 (0.4) |
| Placental complications | P02.0, P02.1, P02.2, P02.3 | 56 (3.8) | 18 (3.0) | 25 (4.2) | 13 (4.8) |
| Premature Rupture of membranes | O42.0, O42.1, P01.1 | 40 (2.7) | 14 (2.3) | 16 (2.7) | 10 (3.7) |
| Preterm labor or delivery | O60.1 | 252 (17.3) | 62 (10.4) | 109 (18.4) | 81 (30.1) |
| Sepsis | A41.8 | 3 (0.2) | 2 (0.3) | 1 (0.2) | 0 (0) |
| Syphilis |  | 3 (0.2) | 1 (0.2) | 0 (0) | 2 (0.7) |
| Umbilical cord complications | P02.4, P02.5 | 5 (0.3) | 1 (0.2) | 2 (0.3) | 2 (0.7) |
| Undetermined | R69 | 1 (0.1) | 1 (0.2) | 0 (0) | 0 (0) |
| Uterine fluid disorders | P01.2, P01.3 | 8 (0.5) | 5 (0.8) | 0 (0) | 3 (1.1) |
| Uterine rupture | O71.1 | 1 (0.1) | 1 (0.2) | 0 (0) | 0 (0) |
| **N10: Miscellaneous** |  | **3 (0.2)** | **0 (0)** | **2 (0.3)** | **1 (0.4)** |
| **N11: Unspecified** |  | **21 (1.4)** | **9 (1.5)** | **10 (1.7)** | **2 (0.7)** |
| Maternal hypertension | P00.0 | 2 (0.1) | 1 (0.2) | 1 (0.2) | 0 (0) |
| Other labor and delivery complications | 63.9 | 1 (0.1) | 1 (0.2) | 0 (0) | 0 (0) |
| Other maternal factor | Z35.6 | 1 (0.1) | 0 (0) | 1 (0.2) | 0 (0) |
| Placental complications | P02.1 | 1 (0.1) | 0 (0) | 1 (0.2) | 0 (0) |
