# Supplementary material for: Causes of death identified in neonates enrolled through Child Health and Mortality Prevention Surveillance (CHAMPS), December 2016 –December 2021
Source: PLOS Glob Public Health. 2023 Mar 20;3(3):e0001612. doi: 10.1371/journal.pgph.0001612 (PMC10027211; doi:10.1371/journal.pgph.0001612)
Supplement: S7 Table — (DOCX) [file pgph.0001612.s008.docx]

| **Supplemental Table 7: Main Maternal condition attributed for neonatal deaths by age group** | | | |  |
| --- | --- | --- | --- | --- |
|  | **Total (N=1458)** | **24 hours death (N=596)** | **END (N=593)** | **LND (N=269)** |
|  | n (%) | n (%) | n (%) | n (%) |
| NULL | 551 (37.8) | 165 (27.7) | 234 (39.5) | 152 (56.5) |
| Maternal hypertension | 149 (10.2) | 49 (8.2) | 76 (12.8) | 24 (8.9) |
| Other labor and delivery complications | 126 (8.6) | 73 (12.2) | 49 (8.3) | 4 (1.5) |
| Multiple gestation | 103 (7.1) | 42 (7.0) | 39 (6.6) | 22 (8.2) |
| Placental complications | 85 (5.8) | 39 (6.5) | 35 (5.9) | 11 (4.1) |
| Other maternal factor | 74 (5.1) | 28 (4.7) | 28 (4.7) | 18 (6.7) |
| Obstructed labor and fetal malpresentation | 67 (4.6) | 42 (7.0) | 24 (4.0) | 1 (0.4) |
| Premature Rupture of membranes | 62 (4.3) | 25 (4.2) | 30 (5.1) | 7 (2.6) |
| Chorioamnionitis and membrane complications | 49 (3.4) | 26 (4.4) | 16 (2.7) | 7 (2.6) |
| Preterm labor or delivery | 43 (2.9) | 25 (4.2) | 12 (2.0) | 6 (2.2) |
| Umbilical cord complications | 24 (1.6) | 16 (2.7) | 7 (1.2) | 1 (0.4) |
| Maternal infection | 22 (1.5) | 11 (1.8) | 9 (1.5) | 2 (0.7) |
| Cervical insufficiency and pelvic anomalies | 17 (1.2) | 12 (2.0) | 3 (0.5) | 2 (0.7) |
| HIV | 10 (0.7) | 3 (0.5) | 5 (0.8) | 2 (0.7) |
| Maternal diabetes | 10 (0.7) | 3 (0.5) | 4 (0.7) | 3 (1.1) |
| Other infections | 9 (0.6) | 1 (0.2) | 5 (0.8) | 3 (1.1) |
| Uterine fluid disorders | 8 (0.5) | 5 (0.8) | 2 (0.3) | 1 (0.4) |
| Other | 7 (0.5) | 6 (1.0) | 1 (0.2) | 0 (0) |
| Other neonatal disorders | 6 (0.4) | 3 (0.5) | 3 (0.5) | 0 (0) |
| Obstructed labor | 5 (0.3) | 4 (0.7) | 1 (0.2) | 0 (0) |
| Undetermined | 5 (0.3) | 3 (0.5) | 2 (0.3) | 0 (0) |
| Syphilis | 4 (0.3) | 2 (0.3) | 2 (0.3) | 0 (0) |
| Uterine rupture | 4 (0.3) | 2 (0.3) | 2 (0.3) | 0 (0) |
| Neonatal preterm birth complications | 2 (0.1) | 2 (0.3) | 0 (0) | 0 (0) |
| Sepsis | 2 (0.1) | 1 (0.2) | 1 (0.2) | 0 (0) |
| Anemias | 1 (0.1) | 1 (0.2) | 0 (0) | 0 (0) |
| Cesarean delivery | 1 (0.1) | 0 (0) | 0 (0) | 1 (0.4) |
| Congenital birth defects | 1 (0.1) | 0 (0) | 0 (0) | 1 (0.4) |
| HIV exposure | 1 (0.1) | 1 (0.2) | 0 (0) | 0 (0) |
| Liver disease | 1 (0.1) | 1 (0.2) | 0 (0) | 0 (0) |
| Malaria | 1 (0.1) | 1 (0.2) | 0 (0) | 0 (0) |
| Malpresentation before labor | 1 (0.1) | 0 (0) | 1 (0.2) | 0 (0) |
| Maternal injury and accident | 1 (0.1) | 1 (0.2) | 0 (0) | 0 (0) |
| Maternal medication or toxic exposure | 1 (0.1) | 0 (0) | 0 (0) | 1 (0.4) |
| Maternal nutritional disorders | 1 (0.1) | 0 (0) | 1 (0.2) | 0 (0) |
| Other | 1 (0.1) | 0 (0) | 1 (0.2) | 0 (0) |
| Other Labor and Delivery Complications | 1 (0.1) | 1 (0.2) | 0 (0) | 0 (0) |
| Other nutritional deficiencies | 1 (0.1) | 1 (0.2) | 0 (0) | 0 (0) |
| Prolapsed cord | 1 (0.1) | 1 (0.2) | 0 (0) | 0 (0) |
