# Supplementary material for: Causes of death identified in neonates enrolled through Child Health and Mortality Prevention Surveillance (CHAMPS), December 2016 –December 2021
Source: PLOS Glob Public Health. 2023 Mar 20;3(3):e0001612. doi: 10.1371/journal.pgph.0001612 (PMC10027211; doi:10.1371/journal.pgph.0001612)
Supplement: S8 Table — (DOCX) [file pgph.0001612.s009.docx]

| Supplemental table 8: Neonatal deaths who had low birth weight in the causal pathway, by age at death | | | |  |
| --- | --- | --- | --- | --- |
|  | All | Death in first 24 hours | Early neonatal death (1-6 days) | Late neonatal death (7-27 days) |
|  | N=1458 | N=596 | N=593 | N=269 |
| Low birth weight in causal pathway | 473 (32.4) | 150 (25.2) | 205 (34.6) | 118 (43.9) |
| Low birth weight not in causal pathway | 985 (67.6) | 446 (74.8) | 388 (65.4) | 151 (56.1) |
