# Supplementary material for: Causes of death identified in neonates enrolled through Child Health and Mortality Prevention Surveillance (CHAMPS), December 2016 –December 2021
Source: PLOS Glob Public Health. 2023 Mar 20;3(3):e0001612. doi: 10.1371/journal.pgph.0001612 (PMC10027211; doi:10.1371/journal.pgph.0001612)
Supplement: S9 Table — (DOCX) [file pgph.0001612.s010.docx]

| **Supplemental Table 9: WHO ICD10 PM underlying cause of death and specific immediate or antecedent causes of death, overall and by age group** | | | | | |
| --- | --- | --- | --- | --- | --- |
| **Underlying causes of death (in bold text) and associated immediate or antecedent causes of death (regular text)** | Number (percent) of deaths with the condition as either an immediate and antecedent cause of death | | | | |
|  | All | Death in first 24 hours | Early neonatal death (1-6 days) | Late neonatal death (7-27 days) |  |
|  | N=1458 | N=596 | N=593 | N=269 |  |
| **Congenital malformation N1** | **118 (8.1)** | **46 (7.7)** | **41 (6.9)** | **31 (11.5)** |  |
| Sepsis | 35 (2.4) | 4 (0.7) | 11 (1.9) | 20 (7.4) |  |
| Lower respiratory infections | 25 (1.7) | 2 (0.3) | 5 (0.8) | 18 (6.7) |  |
| Congenital birth defects | 19 (1.3) | 5 (0.8) | 11 (1.9) | 3 (1.1) |  |
| Perinatal asphyxia/hypoxia | 18 (1.2) | 12 (2.0) | 6 (1.0) | 0 (0) |  |
| Neonatal preterm birth complications | 14 (1.0) | 8 (1.3) | 4 (0.7) | 2 (0.7) |  |
| Meningitis/Encephalitis | 9 (0.6) | 1 (0.2) | 1 (0.2) | 7 (2.6) |  |
| Other | 7 (0.5) | 0 (0) | 2 (0.3) | 5 (1.9) |  |
| Other neonatal disorders | 5 (0.3) | 0 (0) | 3 (0.5) | 2 (0.7) |  |
| Kidney Disease | 4 (0.3) | 0 (0) | 1 (0.2) | 3 (1.1) |  |
| Liver disease | 2 (0.1) | 0 (0) | 0 (0) | 2 (0.7) |  |
| Neonatal aspiration syndromes | 2 (0.1) | 0 (0) | 2 (0.3) | 0 (0) |  |
| Other respiratory disease | 2 (0.1) | 0 (0) | 0 (0) | 2 (0.7) |  |
| Epilepsy | 1 (0.1) | 0 (0) | 1 (0.2) | 0 (0) |  |
| Injury | 1 (0.1) | 0 (0) | 1 (0.2) | 0 (0) |  |
| Neonatal encephalopathy | 1 (0.1) | 1 (0.2) | 0 (0) | 0 (0) |  |
| Other infections | 1 (0.1) | 0 (0) | 0 (0) | 1 (0.4) |  |
| **Disorders related to fetal growth N2** | **11 (0.8)** | **4 (0.7)** | **4 (0.7)** | **3 (1.1)** |  |
| Sepsis | 5 (0.3) | 0 (0) | 2 (0.3) | 3 (1.1) |  |
| Neonatal preterm birth complications | 4 (0.3) | 2 (0.3) | 1 (0.2) | 1 (0.4) |  |
| Congenital birth defects | 2 (0.1) | 1 (0.2) | 0 (0) | 1 (0.4) |  |
| Lower respiratory infections | 2 (0.1) | 0 (0) | 1 (0.2) | 1 (0.4) |  |
| Neonatal encephalopathy | 2 (0.1) | 1 (0.2) | 1 (0.2) | 0 (0) |  |
| Perinatal asphyxia/hypoxia | 2 (0.1) | 2 (0.3) | 0 (0) | 0 (0) |  |
| Meningitis/Encephalitis | 1 (0.1) | 0 (0) | 0 (0) | 1 (0.4) |  |
| Neonatal aspiration syndromes | 1 (0.1) | 1 (0.2) | 0 (0) | 0 (0) |  |
| Other neonatal disorders | 1 (0.1) | 1 (0.2) | 0 (0) | 0 (0) |  |
| **Birth trauma N3** | **1 (0.1)** | **0 (0)** | **1 (0.2)** | **0 (0)** |  |
| Lower respiratory infections | 1 (0.1) | 0 (0) | 1 (0.2) | 0 (0) |  |
| Sepsis | 1 (0.1) | 0 (0) | 1 (0.2) | 0 (0) |  |
| **Complications of intrapartum events N4** | **446 (30.6)** | **252 (42.3)** | **176 (29.7)** | **18 (6.7)** |  |
| Neonatal encephalopathy | 56 (3.8) | 15 (2.5) | 39 (6.6) | 2 (0.7) |  |
| Neonatal preterm birth complications | 40 (2.7) | 25 (4.2) | 13 (2.2) | 2 (0.7) |  |
| Lower respiratory infections | 39 (2.7) | 9 (1.5) | 21 (3.5) | 9 (3.3) |  |
| Neonatal aspiration syndromes | 29 (2.0) | 15 (2.5) | 12 (2.0) | 2 (0.7) |  |
| Other neonatal disorders | 28 (1.9) | 14 (2.3) | 13 (2.2) | 1 (0.4) |  |
| Perinatal asphyxia/hypoxia | 22 (1.5) | 21 (3.5) | 1 (0.2) | 0 (0) |  |
| Meningitis/Encephalitis | 13 (0.9) | 1 (0.2) | 8 (1.3) | 4 (1.5) |  |
| Congenital birth defects | 9 (0.6) | 7 (1.2) | 2 (0.3) | 0 (0) |  |
| Other neurological disorders | 8 (0.5) | 4 (0.7) | 4 (0.7) | 0 (0) |  |
| Anemias | 5 (0.3) | 1 (0.2) | 3 (0.5) | 1 (0.4) |  |
| Other respiratory disease | 5 (0.3) | 3 (0.5) | 1 (0.2) | 1 (0.4) |  |
| Birth trauma | 3 (0.2) | 0 (0) | 3 (0.5) | 0 (0) |  |
| Other | 1 (0.1) | 0 (0) | 1 (0.2) | 0 (0) |  |
| **Convulsion and disorder of cerebral disorders N5** | **16 (1.1)** | **6 (1.0)** | **8 (1.3)** | **2 (0.7)** |  |
| Neonatal aspiration syndromes | 3 (0.2) | 1 (0.2) | 2 (0.3) | 0 (0) |  |
| Sepsis | 2 (0.1) | 0 (0) | 2 (0.3) | 0 (0) |  |
| Neonatal preterm birth complications | 1 (0.1) | 1 (0.2) | 0 (0) | 0 (0) |  |
| **Infections N6** | **254 (17.4)** | **75 (12.6)** | **100 (16.9)** | **79 (29.4)** |  |
| Sepsis | 56 (3.8) | 17 (2.9) | 17 (2.9) | 22 (8.2) |  |
| Lower respiratory infections | 40 (2.7) | 8 (1.3) | 21 (3.5) | 11 (4.1) |  |
| Neonatal preterm birth complications | 34 (2.3) | 13 (2.2) | 17 (2.9) | 4 (1.5) |  |
| Meningitis/Encephalitis | 33 (2.3) | 6 (1.0) | 11 (1.9) | 16 (5.9) |  |
| Perinatal asphyxia/hypoxia | 21 (1.4) | 9 (1.5) | 10 (1.7) | 2 (0.7) |  |
| Other neonatal disorders | 15 (1.0) | 3 (0.5) | 6 (1.0) | 6 (2.2) |  |
| Neonatal aspiration syndromes | 5 (0.3) | 1 (0.2) | 3 (0.5) | 1 (0.4) |  |
| Congenital birth defects | 4 (0.3) | 1 (0.2) | 2 (0.3) | 1 (0.4) |  |
| Anemias | 3 (0.2) | 0 (0) | 2 (0.3) | 1 (0.4) |  |
| Neonatal encephalopathy | 3 (0.2) | 1 (0.2) | 2 (0.3) | 0 (0) |  |
| Other | 3 (0.2) | 1 (0.2) | 2 (0.3) | 0 (0) |  |
| Other neurological disorders | 3 (0.2) | 1 (0.2) | 0 (0) | 2 (0.7) |  |
| Other respiratory disease | 3 (0.2) | 0 (0) | 0 (0) | 3 (1.1) |  |
| Other infections | 2 (0.1) | 0 (0) | 1 (0.2) | 1 (0.4) |  |
| Congenital infection | 1 (0.1) | 1 (0.2) | 0 (0) | 0 (0) |  |
| Diarrheal Diseases | 1 (0.1) | 0 (0) | 1 (0.2) | 0 (0) |  |
| Other disorders of fluid, electrolyte and acid-base balance | 1 (0.1) | 0 (0) | 0 (0) | 1 (0.4) |  |
| Paralytic ileus and intestinal obstruction | 1 (0.1) | 0 (0) | 0 (0) | 1 (0.4) |  |
| **Respiratory and cardiovascular disorders N7** | **159 (10.9)** | **80 (13.4)** | **66 (11.1)** | **13 (4.8)** |  |
| Sepsis | 32 (2.2) | 11 (1.8) | 13 (2.2) | 8 (3.0) |  |
| Neonatal preterm birth complications | 14 (1.0) | 8 (1.3) | 4 (0.7) | 2 (0.7) |  |
| Lower respiratory infections | 10 (0.7) | 5 (0.8) | 3 (0.5) | 2 (0.7) |  |
| Meningitis/Encephalitis | 9 (0.6) | 4 (0.7) | 4 (0.7) | 1 (0.4) |  |
| Other neonatal disorders | 9 (0.6) | 6 (1.0) | 2 (0.3) | 1 (0.4) |  |
| Perinatal asphyxia/hypoxia | 5 (0.3) | 4 (0.7) | 0 (0) | 1 (0.4) |  |
| Neonatal aspiration syndromes | 3 (0.2) | 1 (0.2) | 1 (0.2) | 1 (0.4) |  |
| HIV | 1 (0.1) | 1 (0.2) | 0 (0) | 0 (0) |  |
| Other | 1 (0.1) | 0 (0) | 1 (0.2) | 0 (0) |  |
| **Other neonatal conditions N8** | **25 (1.7)** | **8 (1.3)** | **7 (1.2)** | **10 (3.7)** |  |
| Other neonatal disorders | 7 (0.5) | 2 (0.3) | 2 (0.3) | 3 (1.1) |  |
| Sepsis | 7 (0.5) | 0 (0) | 1 (0.2) | 6 (2.2) |  |
| Neonatal aspiration syndromes | 2 (0.1) | 0 (0) | 2 (0.3) | 0 (0) |  |
| Other respiratory disease | 2 (0.1) | 0 (0) | 1 (0.2) | 1 (0.4) |  |
| Perinatal asphyxia/hypoxia | 2 (0.1) | 2 (0.3) | 0 (0) | 0 (0) |  |
| Congenital birth defects | 1 (0.1) | 0 (0) | 0 (0) | 1 (0.4) |  |
| Lower respiratory infections | 1 (0.1) | 0 (0) | 0 (0) | 1 (0.4) |  |
| Malnutrition | 1 (0.1) | 0 (0) | 0 (0) | 1 (0.4) |  |
| Other | 1 (0.1) | 0 (0) | 0 (0) | 1 (0.4) |  |
| Other infections | 1 (0.1) | 0 (0) | 0 (0) | 1 (0.4) |  |
| **Low birth weight/prematurity complications N9** | **404 (27.7)** | **116 (19.5)** | **178 (30.0)** | **110 (40.9)** |  |
| Neonatal preterm birth complications | 272 (18.7) | 85 (14.3) | 118 (19.9) | 69 (25.7) |  |
| Sepsis | 211 (14.5) | 11 (1.8) | 104 (17.5) | 96 (35.7) |  |
| Lower respiratory infections | 118 (8.1) | 5 (0.8) | 63 (10.6) | 50 (18.6) |  |
| Other neonatal disorders | 90 (6.2) | 6 (1.0) | 42 (7.1) | 42 (15.6) |  |
| Meningitis/Encephalitis | 82 (5.6) | 2 (0.3) | 52 (8.8) | 28 (10.4) |  |
| Perinatal asphyxia/hypoxia | 38 (2.6) | 21 (3.5) | 13 (2.2) | 4 (1.5) |  |
| Other | 11 (0.8) | 0 (0) | 9 (1.5) | 2 (0.7) |  |
| Anemias | 5 (0.3) | 0 (0) | 1 (0.2) | 4 (1.5) |  |
| Congenital birth defects | 5 (0.3) | 1 (0.2) | 3 (0.5) | 1 (0.4) |  |
| Kidney Disease | 5 (0.3) | 0 (0) | 0 (0) | 5 (1.9) |  |
| Other endocrine, metabolic, blood, and immune disorders | 5 (0.3) | 0 (0) | 5 (0.8) | 0 (0) |  |
| Other infections | 5 (0.3) | 0 (0) | 0 (0) | 5 (1.9) |  |
| Other respiratory disease | 4 (0.3) | 0 (0) | 0 (0) | 4 (1.5) |  |
| Neonatal aspiration syndromes | 3 (0.2) | 0 (0) | 3 (0.5) | 0 (0) |  |
| Congenital infection | 2 (0.1) | 1 (0.2) | 1 (0.2) | 0 (0) |  |
| Liver disease | 2 (0.1) | 0 (0) | 1 (0.2) | 1 (0.4) |  |
| Birth trauma | 1 (0.1) | 1 (0.2) | 0 (0) | 0 (0) |  |
| Neonatal encephalopathy | 1 (0.1) | 0 (0) | 1 (0.2) | 0 (0) |  |
| Other disorders of fluid, electrolyte and acid-base balance | 1 (0.1) | 0 (0) | 1 (0.2) | 0 (0) |  |
| Paralytic ileus and intestinal obstruction | 1 (0.1) | 0 (0) | 1 (0.2) | 0 (0) |  |
| Placental complications | 1 (0.1) | 0 (0) | 0 (0) | 1 (0.4) |  |
