# Supplementary material for: Causes of death identified in neonates enrolled through Child Health and Mortality Prevention Surveillance (CHAMPS), December 2016 –December 2021
Source: PLOS Glob Public Health. 2023 Mar 20;3(3):e0001612. doi: 10.1371/journal.pgph.0001612 (PMC10027211; doi:10.1371/journal.pgph.0001612)
Supplement: S10 Table — (DOCX) [file pgph.0001612.s011.docx]

| Supplemental table 10: Pathogens identified in neonatal deaths in which infection was determined in the causal pathway, by age at death | | | | |
| --- | --- | --- | --- | --- |
| **Pathogen** | All | Death in first 24 hours | Early neonate deaths | Late neonate deaths |
|  | N=590 | N=104 | N=272 | N=214 |
| **Bacteria** |  |  |  |  |
| **Gram Negative Bacteria** | **492 (83.4)** | **62 (59.6)** | **245 (90.1)** | **185 (86.4)** |
| *Acinetobacter baumannii* | 213 (36.1) | 5 (4.8) | 110 (40.4) | 98 (45.8) |
| *Acinetobacter spp.* | 2 (0.3) | 1 (1.0) | 0 (0) | 1 (0.5) |
| *Bordetella spp.* | 1 (0.2) | 0 (0) | 1 (0.4) | 0 (0) |
| *Burkholderia cepacia* | 1 (0.2) | 0 (0) | 0 (0) | 1 (0.5) |
| *Chlamydia trachomatis* | 1 (0.2) | 1 (1.0) | 0 (0) | 0 (0) |
| *Citrobacter freundii* | 2 (0.3) | 0 (0) | 0 (0) | 2 (0.9) |
| *Citrobacter sedlakii* | 1 (0.2) | 0 (0) | 0 (0) | 1 (0.5) |
| *Enterobacter cloacae* | 9 (1.5) | 1 (1.0) | 6 (2.2) | 2 (0.9) |
| *Enterobacter spp.* | 2 (0.3) | 0 (0) | 1 (0.4) | 1 (0.5) |
| *Escherichia coli* | 75 (12.7) | 22 (21.2) | 29 (10.7) | 24 (11.2) |
| *Escherichia coli/Shigella spp.* | 5 (0.8) | 1 (1.0) | 3 (1.1) | 1 (0.5) |
| *Haemophilus influenzae* | 6 (1.0) | 2 (1.9) | 0 (0) | 4 (1.9) |
| *Haemophilus influenzae Type A* | 4 (0.7) | 0 (0) | 1 (0.4) | 3 (1.4) |
| *Haemophilus spp.* | 1 (0.2) | 0 (0) | 0 (0) | 1 (0.5) |
| *Klebsiella oxytoca* | 3 (0.5) | 0 (0) | 1 (0.4) | 2 (0.9) |
| *Klebsiella pneumoniae* | 268 (45.4) | 32 (30.8) | 137 (50.4) | 99 (46.3) |
| *Klebsiella spp.* | 1 (0.2) | 0 (0) | 1 (0.4) | 0 (0) |
| *Klebsiella terragina* | 1 (0.2) | 0 (0) | 1 (0.4) | 0 (0) |
| *Moraxella catarrhalis* | 3 (0.5) | 0 (0) | 0 (0) | 3 (1.4) |
| *Morganella morganii* | 2 (0.3) | 1 (1.0) | 1 (0.4) | 0 (0) |
| *Myroides spp.* | 1 (0.2) | 0 (0) | 0 (0) | 1 (0.5) |
| *Neisseria meningitidis* | 1 (0.2) | 0 (0) | 0 (0) | 1 (0.5) |
| *Pantoea agglomerans* | 1 (0.2) | 1 (1.0) | 0 (0) | 0 (0) |
| *Pantoea spp.* | 1 (0.2) | 0 (0) | 1 (0.4) | 0 (0) |
| *Proteus mirabilis* | 2 (0.3) | 0 (0) | 1 (0.4) | 1 (0.5) |
| *Proteus vulgaris* | 1 (0.2) | 0 (0) | 0 (0) | 1 (0.5) |
| *Providencia stuartii* | 1 (0.2) | 0 (0) | 0 (0) | 1 (0.5) |
| *Pseudomonas aeruginosa* | 31 (5.3) | 2 (1.9) | 15 (5.5) | 14 (6.5) |
| *Pseudomonas spp.* | 1 (0.2) | 0 (0) | 1 (0.4) | 0 (0) |
| *Salmonella spp.* | 8 (1.4) | 0 (0) | 6 (2.2) | 2 (0.9) |
| *Serratia marcescens* | 3 (0.5) | 0 (0) | 1 (0.4) | 2 (0.9) |
| *Shewanalla putrefaciens* | 1 (0.2) | 1 (1.0) | 0 (0) | 0 (0) |
| *Shigella spp.* | 1 (0.2) | 0 (0) | 0 (0) | 1 (0.5) |
| *Stenotrophomonas maltophilia* | 1 (0.2) | 0 (0) | 1 (0.4) | 0 (0) |
| *Treponema pallidum* | 7 (1.2) | 3 (2.9) | 4 (1.5) | 0 (0) |
| *Ureaplasma spp.* | 7 (1.2) | 3 (2.9) | 4 (1.5) | 0 (0) |
| **Gram Positive bacteria** | **170 (28.8)** | **47 (45.2)** | **58 (21.3)** | **65 (30.4)** |
| *Coagulase negative Staphylococcus* | 3 (0.5) | 1 (1.0) | 2 (0.7) | 0 (0) |
| *Enterococcus faecalis* | 27 (4.6) | 6 (5.8) | 10 (3.7) | 11 (5.1) |
| *Enterococcus faecium* | 22 (3.7) | 1 (1.0) | 7 (2.6) | 14 (6.5) |
| *Enterococcus spp.* | 4 (0.7) | 0 (0) | 2 (0.7) | 2 (0.9) |
| *Listeria monocytogenes* | 6 (1.0) | 1 (1.0) | 3 (1.1) | 2 (0.9) |
| *Staphylococcus aureus* | 32 (5.4) | 0 (0) | 13 (4.8) | 19 (8.9) |
| *Staphylococcus spp.* | 2 (0.3) | 1 (1.0) | 0 (0) | 1 (0.5) |
| *Streptococcus agalactiae* | 47 (8.0) | 28 (26.9) | 11 (4.0) | 8 (3.7) |
| *Streptococcus Group G* | 1 (0.2) | 0 (0) | 0 (0) | 1 (0.5) |
| *Streptococcus pneumoniae* | 19 (3.2) | 4 (3.8) | 9 (3.3) | 6 (2.8) |
| *Streptococcus pyogenes* | 2 (0.3) | 0 (0) | 2 (0.7) | 0 (0) |
| *Streptococcus spp.* | 22 (3.7) | 7 (6.7) | 7 (2.6) | 8 (3.7) |
| *Streptococcus viridans* | 1 (0.2) | 0 (0) | 1 (0.4) | 0 (0) |
| **Virus** | **33 (5.6)** | **5 (4.8)** | **9 (3.3)** | **19 (8.9)** |
| Cytomegalovirus | 11 (1.9) | 3 (2.9) | 7 (2.6) | 1 (0.5) |
| Dengue | 1 (0.2) | 0 (0) | 0 (0) | 1 (0.5) |
| Enterovirus | 2 (0.3) | 0 (0) | 1 (0.4) | 1 (0.5) |
| Herpes simplex virus 2 | 1 (0.2) | 0 (0) | 0 (0) | 1 (0.5) |
| Human coronavirus HKU1 | 1 (0.2) | 1 (1.0) | 0 (0) | 0 (0) |
| Human Immunodeficiency Virus | 2 (0.3) | 1 (1.0) | 1 (0.4) | 0 (0) |
| Influenza B | 1 (0.2) | 0 (0) | 0 (0) | 1 (0.5) |
| Measles | 1 (0.2) | 0 (0) | 0 (0) | 1 (0.5) |
| Parainfluenza virus type 3 | 2 (0.3) | 0 (0) | 0 (0) | 2 (0.9) |
| Parechovirus | 1 (0.2) | 0 (0) | 0 (0) | 1 (0.5) |
| Respiratory syncytial virus | 6 (1.0) | 0 (0) | 0 (0) | 6 (2.8) |
| Rhinovirus | 1 (0.2) | 0 (0) | 0 (0) | 1 (0.5) |
| SARS-CoV-2 | 3 (0.5) | 0 (0) | 0 (0) | 3 (1.4) |
| **Fungi** | **43 (7.3)** | **2 (1.9)** | **6 (2.2)** | **35 (16.4)** |
| *Candida albicans* | 17 (2.9) | 1 (1.0) | 3 (1.1) | 13 (6.1) |
| *Candida auris* | 4 (0.7) | 0 (0) | 0 (0) | 4 (1.9) |
| *Candida glabrata* | 7 (1.2) | 0 (0) | 2 (0.7) | 5 (2.3) |
| *Candida parapsilosis* | 6 (1.0) | 0 (0) | 0 (0) | 6 (2.8) |
| *Candida spp.* | 8 (1.4) | 2 (1.9) | 1 (0.4) | 5 (2.3) |
| *Candida tropicalis* | 1 (0.2) | 0 (0) | 0 (0) | 1 (0.5) |
| *Cryptococcus laurentii* | 1 (0.2) | 0 (0) | 0 (0) | 1 (0.5) |
| *Kodamaea ohmeri* | 1 (0.2) | 0 (0) | 0 (0) | 1 (0.5) |
| **Parasite** | **2 (0.3)** | **1 (1.0)** | **0 (0)** | **1 (0.5)** |
| *Toxoplasma gondii* | 2 (0.3) | 1 (1.0) | 0 (0) | 1 (0.5) |
