# Supplementary material for: Causes of death identified in neonates enrolled through Child Health and Mortality Prevention Surveillance (CHAMPS), December 2016 –December 2021
Source: PLOS Glob Public Health. 2023 Mar 20;3(3):e0001612. doi: 10.1371/journal.pgph.0001612 (PMC10027211; doi:10.1371/journal.pgph.0001612)
Supplement: S11 Table — a. Expert (DeCoDe) panel determination if neonatal deaths were preventable, overall and by age group. b. Recommended improvements that could prevent preventable deaths, overall and by age group. (ZIP) [file pgph.0001612.s012.zip › S11a_Table.docx]

| Supplemental Table 11a: Expert (DeCoDe) panel determination if neonatal deaths were preventable, overall and by age group | | | | |
| --- | --- | --- | --- | --- |
|  | Total | Death in first 24 hours | Early Neonate death | Late Neonate death |
| Preventable | 1025 (70.3) | 405 (68.0) | 410 (69.1) | 210 (78.1) |
| Preventable under certain conditions | 75 (5.1) | 23 (3.9) | 37 (6.2) | 15 (5.6) |
| Not preventable | 339 (23.3) | 157 (26.3) | 140 (23.6) | 42 (15.6) |
| Not reported | 19 (1.3) | 11 (1.8) | 6 (1.0) | 2 (0.7) |
