# Supplementary material for: Causes of death identified in neonates enrolled through Child Health and Mortality Prevention Surveillance (CHAMPS), December 2016 –December 2021
Source: PLOS Glob Public Health. 2023 Mar 20;3(3):e0001612. doi: 10.1371/journal.pgph.0001612 (PMC10027211; doi:10.1371/journal.pgph.0001612)
Supplement: S11 Table — a. Expert (DeCoDe) panel determination if neonatal deaths were preventable, overall and by age group. b. Recommended improvements that could prevent preventable deaths, overall and by age group. (ZIP) [file pgph.0001612.s012.zip › S11b_Table.docx]

| Supplemental Table 11b: Recommended improvements that could prevent preventable deaths, overall and by age group | | | | |
| --- | --- | --- | --- | --- |
|  | Total (N=1458) | 24 hours death (N=596) | END (N=593) | LND (N=269) |
| Improved infection prevention and control | 300 (20.6) | 28 (4.7) | 145 (24.5) | 127 (47.2) |
| Improved HIV prevention and control | 19 (1.3) | 3 (0.5) | 8 (1.3) | 8 (3.0) |
| Improved ANC and obstetric care and management | 538 (36.9) | 269 (45.1) | 217 (36.6) | 52 (19.3) |
| Improved family planning | 44 (3.0) | 25 (4.2) | 13 (2.2) | 6 (2.2) |
| Improved clinical management and quality of care | 537 (36.8) | 226 (37.9) | 231 (39.0) | 80 (29.7) |
| Improved health education | 56 (3.8) | 14 (2.3) | 19 (3.2) | 23 (8.6) |
| Improved health-seeking behavior | 161 (11.0) | 75 (12.6) | 46 (7.8) | 40 (14.9) |
| Improved nutritional support | 19 (1.3) | 4 (0.7) | 6 (1.0) | 9 (3.3) |
| Improved transport system | 30 (2.1) | 17 (2.9) | 10 (1.7) | 3 (1.1) |
