# Supplementary material for: Causes of death identified in neonates enrolled through Child Health and Mortality Prevention Surveillance (CHAMPS), December 2016 –December 2021
Source: PLOS Glob Public Health. 2023 Mar 20;3(3):e0001612. doi: 10.1371/journal.pgph.0001612 (PMC10027211; doi:10.1371/journal.pgph.0001612)

Supplementary Figure 1: Main maternal condition attributed to neonatal death, by age at death (first 24 hours, early neonatal death [1-6 days], late neonatal death [7-27 days]).


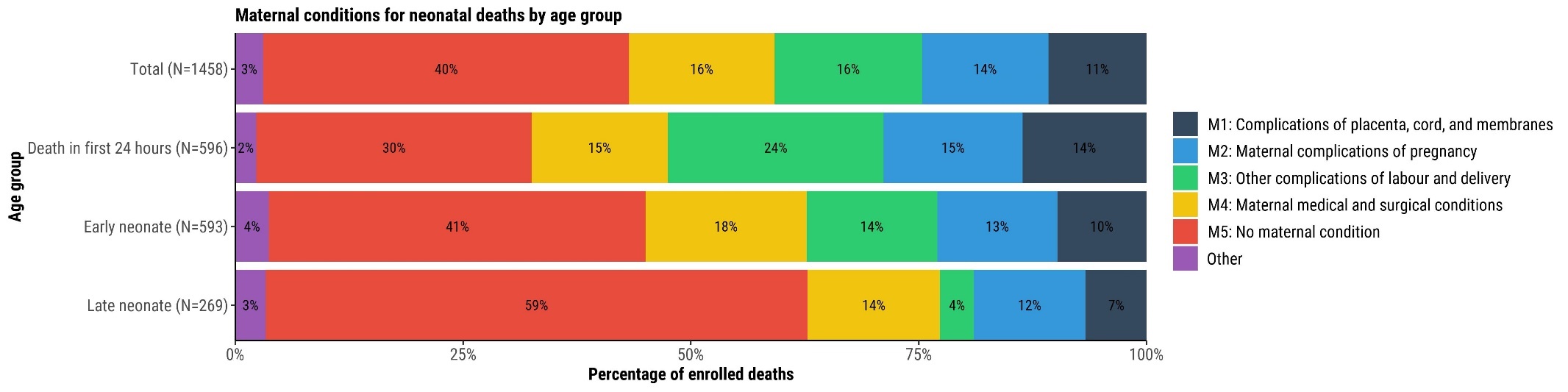

Supplement: S1 Fig — (DOCX) [file pgph.0001612.s013.docx]
