# Supplementary material for: Causes of death identified in neonates enrolled through Child Health and Mortality Prevention Surveillance (CHAMPS), December 2016 –December 2021
Source: PLOS Glob Public Health. 2023 Mar 20;3(3):e0001612. doi: 10.1371/journal.pgph.0001612 (PMC10027211; doi:10.1371/journal.pgph.0001612)

Supplementary figure 2: Number of other conditions in the causal chain for CHAMPS deaths that occurred in the neonatal period, by age group


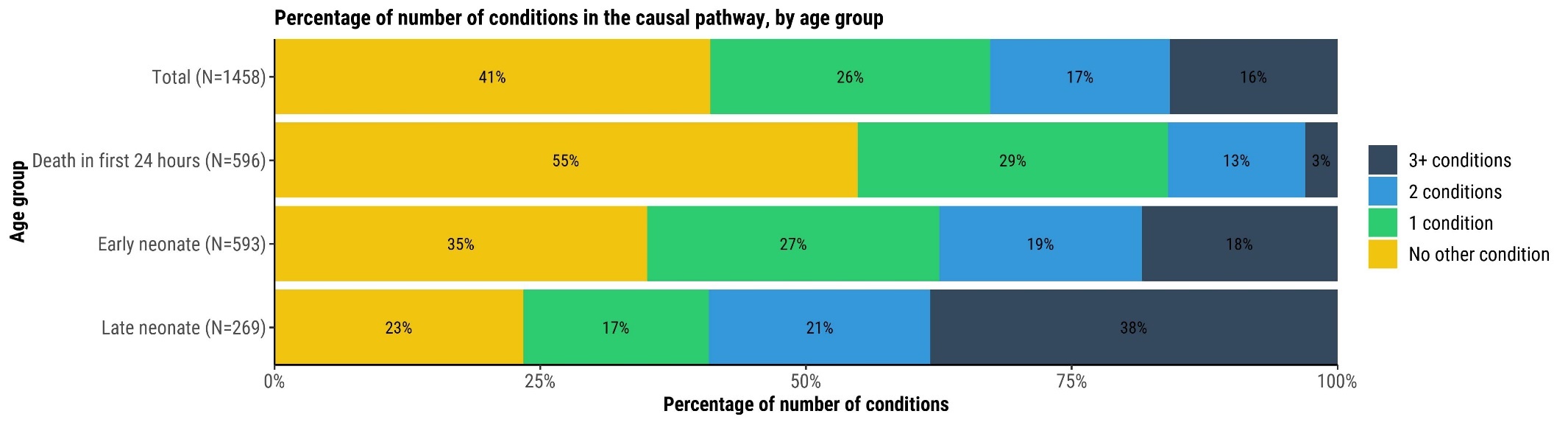

Supplement: S2 Fig — (DOCX) [file pgph.0001612.s014.docx]
