# Supplementary figures and images for: Causes of death identified in neonates enrolled through Child Health and Mortality Prevention Surveillance (CHAMPS), December 2016 –December 2021
Source: PLOS Glob Public Health. 2023 Mar 20;3(3):e0001612. doi: 10.1371/journal.pgph.0001612 (PMC10027211; doi:10.1371/journal.pgph.0001612)

Supplemental figure 3: Comparison of MITS vs non- MITS cause of deaths


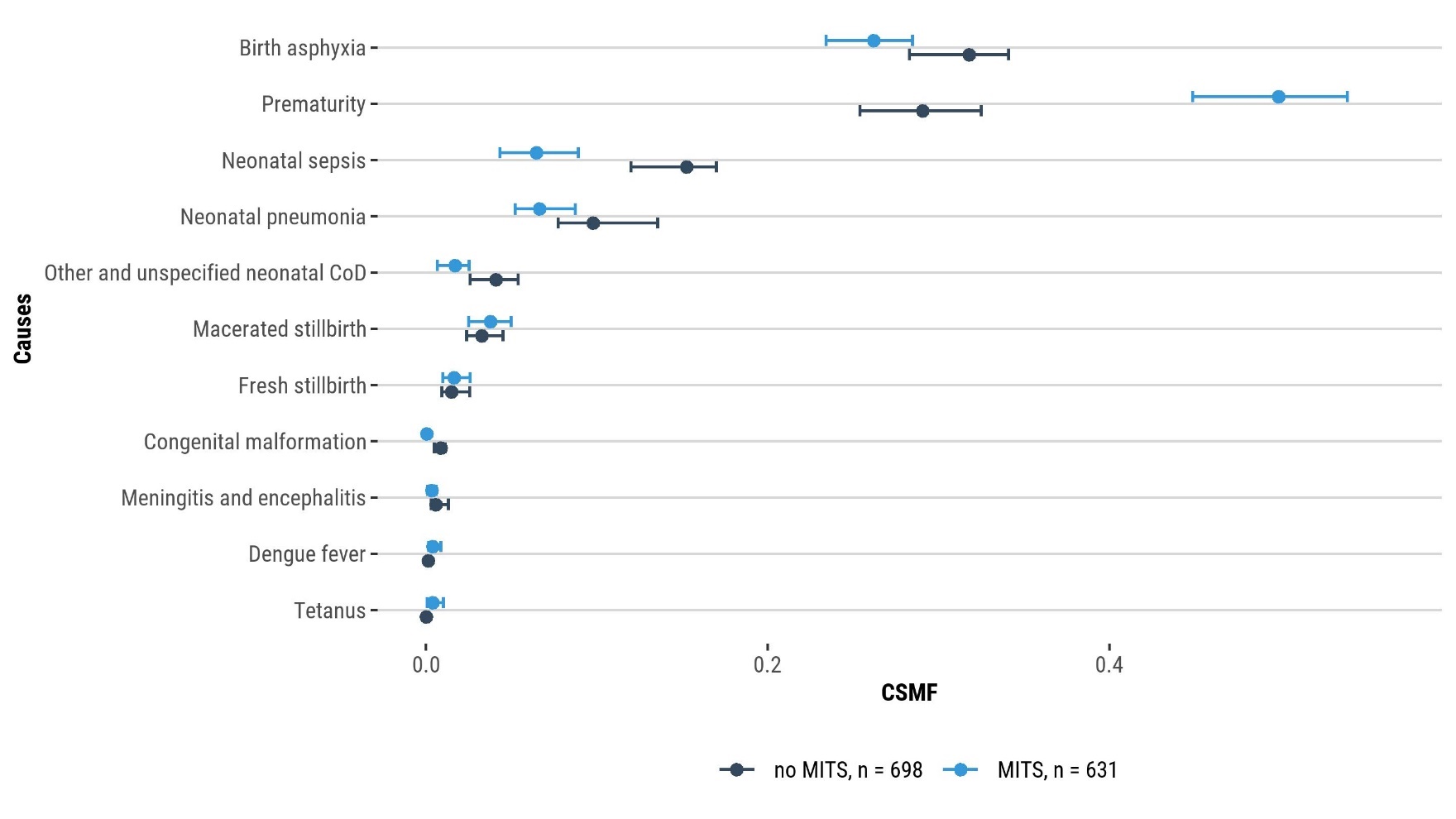

Supplement: S3 Fig — (DOCX) [file pgph.0001612.s015.docx]
